# Mitochondrial glutamine transporter SLC1A5_var, a potential target to suppress astrocyte reactivity in Parkinson’s Disease

**DOI:** 10.1038/s41419-022-05399-z

**Published:** 2022-11-09

**Authors:** Yang Liu, Lei Cao, Yuting Song, Zhengwei Kang, Ting Liu, Jianhua Ding, Gang Hu, Ming Lu

**Affiliations:** 1 Department of Pharmacology, School of Medicine and Holistic Integrative Medicine, Nanjing University of Chinese Medicine, Nanjing, 210023 China; 2 Jiangsu Key Laboratory of Neurodegeneration, Department of Pharmacology, Nanjing Medical University, Nanjing, 211116 China; 3 Neuroprotective Drug Discovery Key Laboratory, Department of Pharmacology, Nanjing Medical University, Nanjing, 211166 China

**Keywords:** Microglia, Parkinson's disease, Transporters in the nervous system

## Abstract

SLC1A5 variant (SLC1A5_var) is identified as a mitochondrial glutamine transporter in cancer cells recently. However, the role of SLC1A5_var in Parkinson’s disease (PD) is completely unknown. Here, we found the significant downregulation of SLC1A5_var in astrocytes and midbrain of mice treated with MPTP/MPP^+^ and LPS. Importantly, overexpression of SLC1A5_var ameliorated but knockdown of SLC1A5_var exacerbated MPTP/MPP^+^- and LPS-induced mitochondrial dysfunction. Consequently, SLC1A5_var provided beneficial effects on PD pathology including improvement of PD-like motor symptoms and rescue of dopaminergic (DA) neuron degeneration through maintaining mitochondrial energy metabolism. Moreover, SLC1A5_var reduced astrocyte reactivity via inhibition of A1 astrocyte conversion. Further investigation demonstrated that SLC1A5_var restrained the secretion of astrocytic pro-inflammatory cytokines by blunting TLR4-mediated downstream pathways. This is the first study to prove that astrocytic SLC1A5_var inhibits neuroinflammation, and rescues the loss of DA neurons and motor symptoms involved in PD progression, which provides a novel target for PD treatment.

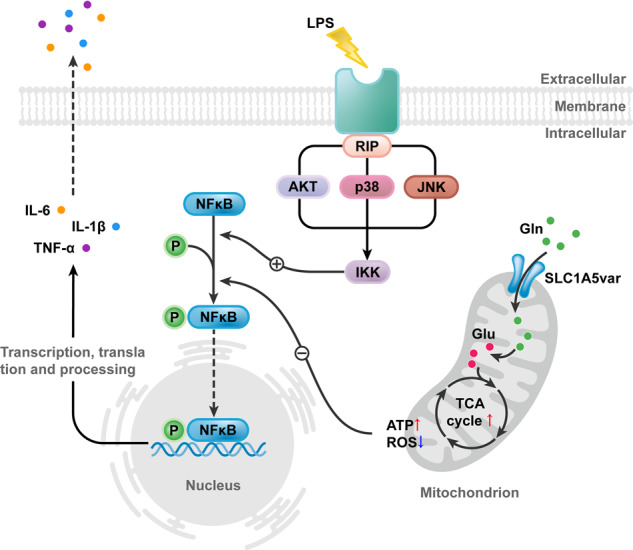

## Introduction

Parkinson’s disease (PD) is a neurodegenerative disorder characterized by motor and non-motor symptoms. The pathological feature of PD is the progressive loss of dopaminergic (DA) neurons in the substantia nigra pars compacta (SNpc), accompanied by the formation of Lewy bodies and the activation of glial cells [1, 2]. Studies of genetics and genomics in PD have demonstrated that oxidative stress and mitochondrial dysfunction play vital roles in the progression of PD [3]. For this reason, theoretically, drugs targeting mitochondria are promising for PD treatment. However, MitoQ as a powerful mitochondrial antioxidant failed to slow the progression of PD in clinical trial [4], indicating a complicated mechanism underlying the mitochondrial dysfunction in PD pathogenesis. Further investigation is warranted to elucidate the sophisticated regulation involved in mitochondrial dysfunction.

Astrocytes are the most abundant cells responsible for maintaining the overall homeostasis in the brain. Our previous studies have proved that dramatic increase in astrocyte reactivity triggers neuroinflammation and accelerates PD progression, while inhibition of astrocyte-derived inflammation is able to improve PD symptoms [5–7], suggesting that retaining astrocyte physiological properties could be a potential strategy for PD therapy. Numerous evidence has affirmed mitochondrial dysfunction as a major contributor to astrocyte transformation from resting state to neurotoxic A1 state [8, 9]. Although mitochondrial dysfunction is commonly observed in astrocytes during the development of PD [10], the molecular events taking place within the pathogenic process are poorly documented. Depicting the molecular cascade in astrocyte activated parallel to PD pathogenesis could provide comprehensive understanding of mitochondrial dysfunction and facilitate mitochondria-targeting strategies for PD therapy.

In this study, RNA sequencing was performed to analyze the transcriptome of astrocytes treated with MPP^+^. Beyond confirming mitochondrial dysfunction and oxidative stress as key pathogenesis of PD, we also found that SLC1A5_variant (SLC1A5_var) located in the mitochondrion is required for mitochondrial energy metabolism. SLC1A5_var is the variant form of SLC1A5 which is a sodium-dependent transporter of neutral amino acids. Unlike SLC1A5 localized in the plasma membrane, SLC1A5_var harbors N-terminal targeting signal for mitochondrial localization that enables it to regulate mitochondrial function [11]. Our study first identified that SLC1A5_var is a crucial regulator of mitochondrial energy metabolism in astrocytes. Meanwhile, overexpression of SLC1A5_var preserves astrocyte physiological properties against neuroinflammation and PD symptoms. Together, these findings broaden our knowledge of the mechanism underlying mitochondrial dysfunction and shed light on mitochondria-targeting therapeutics for PD.

## Materials and methods

### Experimental animals

C57BL/6J mice (male, 3-month old) used in this experiment were purchased from Nanjing Medical University. The mice used in the experiment were housed with temperatures of 22-25 °C and humidity of 50-60%. Mice were housed at groups of 5 per cage with water and food *ad libitum* on a 12:12 h light cycle (lights on at 7 a.m.). All animal procedures were performed in accordance with the guideline of the Institutional Animal Care and Use Committee of Nanjing Medical University.

### Stereotaxic surgery

According to previous literature [12], mice were pretreated with sodium pentobarbital anesthesia (40 mg.kg^−1^, sigma). The Lentivirus (LV) overexpressing SLC1A5_var (NCBI Reference Sequence: NM-001145145.1) and the corresponding empty vector were injected into the mice’s midbrain. The three-dimensional coordinates of the mice’s midbrain are as follows: A/P −3.0 mm, R/L ±1.3 mm, and D/V −4.5 mm. The mice were microinjected with either a SLC1A5_var vector lentivirus (SLC1A5_var vec) or SLC1A5_var overexpression lentivirus (SLC1A5_var OE) (1.5 μL of 2 × 10^9^ viral genome/uL, Hanbio).

### Preparation of animal models

For MPTP model, mice were divided into four groups 2 weeks after LV microinjection: SLC1A5_var vec +Saline, SLC1A5_var overexpression (OE) + Saline, SLC1A5_var vec +MPTP, SLC1A5_var OE +MPTP. Mice were weighed and injected subcutaneously with MPTP at 20 mg/kg for 5 consecutive days. 3 days after the completion of modeling, behavioral tests were conducted and then mice were sacrificed for bioassay.

For LPS model, mice were divided into four groups 2 weeks after LV microinjection: SLC1A5_var vec +Saline, SLC1A5_var overexpression (OE) +Saline, SLC1A5_var vec +LPS, SLC1A5_var OE +LPS. Injection of LPS into mice’s midbrain was carried out as per the method of the lentivirus injection. LPS was dissolved in saline at a concentration of 2.5 mg/ml, and 1 μL was injected on both sides. 7 days later, behavioral tests were conducted and then mice were sacrificed for bioassay.

### Behavioral test

Open field test is mainly used to observe the ability of voluntary movement. The mice were firstly acclimatized in the room for 2 h. Then the overall distance traveled (mm) and speed (cm/s) which represent the spontaneous activities were recorded in 5 min by the software (Top Scan Version 2.0, American CleverSys Inc).

Pole test is widely used to assess basal ganglia related movement disorders in mice. Time expenditure for climbing from top to bottom of a pole (landing on both front claws) was recorded, and mice with motor coordination defect consume more time. Firstly, the mice were placed on a smooth pole (diameter of 1 cm and height of 50 cm). Then the mice were trained 3 times and the total time consumed from top to bottom was recorded.

Rotarod test is performed to assess motor coordination and balance of mice. Firstly, the mice were trained on an accelerating rotarod rod in a separate compartment. Then, the mice were tested at 20 rpm for 180 s. The latency to fall off the rod was recorded by the JLBehv-RRTG-5 system.

### Cell culture and transfection

Astrocytes and microglia were isolated as previously [7]. Briefly, primary astrocytes were isolated from the brain of neonatal mice (3 days after birth) and cultured in DMEM/F12 medium (#10565018, GIBCO) supplied with 10% FBS and 100 unit/mL penicillin/streptomycin. Change the medium every 3 days and culture for 14 days. Primary microglia were isolated from the brain tissues of neonatal mice (3 days after birth). Microglia were seeded on the T75 precoated with poly-lysine and the culture medium is the same as astrocytes. After being cultured for 14 days, microglia were collected by shaking at 200 rpm for 15 min. Then microglia-conditioned medium (MCM) was collected and centrifuged at 12,000 × *g* for 10 min at 4 °C and the supernatant was collected to stimulate the A1-type astrocytes. Complete (100%) microglia medium was added to the astrocytes, and the volume was 1.0 ml for each well of 6-well plate or 0.5 ml for each well of 12-well plate.

For the cell lines, C8-D1A (No. CRL-2541, ATCC) and HEK-293T cells were cultured in DMEM medium (#11965092, GIBCO) supplied with 10% FBS and 100 unit/mL penicillin/streptomycin.

For transfection, SLC1A5_var siRNA was purchased from GenePharma (Shanghai, China). Astrocytes were cultured for 14 days in vitro and transfection was performed on Day 14 using Lipofectamine 3000 (Invitrogen, L3000015) according to the manufacturer’s protocol. Then, experiments were carried out 48 hours after transfection. The sequence of SLC1A5_var siRNA was listed in Supplementary Table S1. For overexpression of SLC1A5_var, *Slc1a5_var* cDNA and its truncations were amplified and subcloned into pcDNA3.1 (PPL, Nanjing, China). Then the plasmid was transfected into the astrocytes and HEK-293T cells.

### RNA extraction and library construction

Total RNA was isolated from primary astrocytes (*N* = 3) with or without MPP^+^ (500 μM, 24 h) treatment. Then RNA was purified using TRIzol reagent (Invitrogen, Carlsbad, CA, USA) following the manufacturer’s procedure. The RNA amount and purity of each sample was quantified using NanoDrop ND-1000 (NanoDrop, Wilmington, DE, USA). The RNA integrity was assessed by Bioanalyzer 2100 (Agilent, CA, USA) with RIN number >7.0, and confirmed by electrophoresis with denaturing agarose gel. Poly (A) RNA is purified from 1 μg total RNA using Dynabeads Oligo (dT)25-61005 (Thermo Fisher, CA, USA) using two rounds of purification. Then the poly(A) RNA was fragmented into small pieces using Magnesium RNA Fragmentation Module (NEB, cat. e6150, USA) under 94 °C 5–7 min. Then the cleaved RNA fragments were reverse-transcribed to create the cDNA by SuperScript™ II Reverse Transcriptase (Invitrogen, cat. 1896649, USA), which were next used to synthesize U-labeled second-stranded DNAs with E. coli DNA polymerase I (NEB, cat.m0209, USA), RNase H (NEB, cat.m0297, USA) and dUTP Solution (Thermo Fisher, cat. R0133, USA). An A-base is then added to the blunt ends of each strand, preparing them for ligation to the indexed adapters. Each adapter contains a T-base overhang for ligating the adapter to the A-tailed fragmented DNA. Single- or dual-index adapters are ligated to the fragments, and size selection was performed with AMPureXP beads. After the heat-labile UDG enzyme (NEB, cat.m0280, USA) treatment of the U-labeled second-stranded DNAs, the ligated products are amplified with PCR by the following conditions: initial denaturation at 95 °C for 3 min; 8 cycles of denaturation at 98 °C for 15 s, annealing at 60 °C for 15 s, and extension at 72 °C for 30 s; and then final extension at 72 °C for 5 min. The average insert size for the final cDNA library was 300 ± 50 bp. At last, we performed the 2 × 150 bp paired-end sequencing (PE150) on an illumina Novaseq™ 6000 (LC-Bio Technology CO., Ltd., Hangzhou, China) following the recommended protocol.

### Bioinformatics analysis of RNA-seq

Fastp software (https://github.com/OpenGene/fastp) was used to remove the reads that contained adaptor contamination, low-quality bases, and undetermined bases with default parameter. Then sequence quality was also verified using fastp. We used HISAT2 (https://ccb.jhu.edu/software/hisat2) to map reads to the reference genome of *Mus musculus* (GRCm38). The mapped reads of each sample were assembled using StringTie (https://ccb.jhu.edu/software/stringtie) with default parameters. Then, all transcriptomes from all samples were merged to reconstruct a comprehensive transcriptome using gffcompare (https://github.com/gpertea/gffcompare/). After the final transcriptome was generated, StringTie and was used to estimate the expression levels of all transcripts. StringTie was used to perform expression level for mRNAs by calculating FPKM (FPKM = [total_exon_fragments/mapped_reads(millions) × exon_length(kB)]). The differentially expressed mRNAs were selected with fold change > 2 or fold change < 0.5 and with parametric F-test comparing nested linear models (*p*-value < 0.05, *q* value was used for the *p*-value correction) by R package edgeR (https://bioconductor.org/packages/release/bioc/html/edgeR.html). The differentially expressed genes associated with mitochondrial function and oxidative stress, two major causes of PD, were selected further and enriched using Gene Ontology (http://geneontology.org/). Genes were further validated by qRT-PCR experiments.

### Measurement of mitochondrial oxygen consumption rate (OCR)

The oxygen consumption rate of primary astrocytes was measured using the Seahorse XF96 (Agilent, CA, USA). Astrocytes were seeded on the plates (20,000 cells/per well) and grouped into four: SLC1A5_var vec, SLC1A5_var OE, SLC1A5_var vec +LPS, and SLC1A5_var OE +LPS. Oxygen consumption rates were measured after injection of oligomycin (1 μM), FCCP (1 μM), and antimycin A (Mitochondrial Complex III inhibitor, 0.5 μM) plus rotenone (Mitochondrial Complex I inhibitor, 0.5 μM). The non-mitochondrial respiration was not subtracted for the quantification.

### Immunofluorescence and immunohistochemistry

Immunofluorescence and immunohistochemistry were performed as described in our previous study [13]. Cells or brain slices (frozen sections, 25 μm) were fixed in 4% paraformaldehyde and blocked with 5% BSA in PBST (0.3% Triton X-100), and then they were incubated with the primary antibody at 4 °C overnight. After that, the corresponding second antibodies were incubated for 1 h at room temperature. The fluorescence intensity was quantified by Image J software (v.1.8.0). Briefly, same color threshold was applied for all images, corrected fluorescence was obtained using “analyze particles” function by subtracting out background signal. Stereology using the optical fractionator (Stereo Investigator 7, MBF Bioscience, Williston, VT) was performed for cell counting. Every sixth coronal frozen section was collected for quantification. Briefly, the regions of SNpc in the midbrain sections were outlined at low magnification (40×). The counting frame size was 50 μm × 50 μm and the sampling grid size was 100 μm × 100 μm. All stereological analyses were performed under the 200× magnification of an Olympus BX52 microscope (Olympus America Inc., Melville, NY).

The antibodies used for immunofluorescent and immunohistochemistry staining were shown in Supplementary Table S2. Hoechst (Invitrogen, #33258) was added and incubated within brain slices or cell coverslips for 5 min. Then the dye was Removed and rinsed three times in PBS. Mount the coverslips in Microscope Slides and capture by CarlZeiss LSM710 confocal microscope.

### Mitochondria isolation

Astrocytes and HEK-293T cells were transfected with SLC1A5_var and mitochondria were isolated using Cell Mitochondrial Extraction Kit (C3601, Beyotime) as per the manufactory’s protocol. Briefly, cells were harvested and the mitochondrial isolation reagent provided by the kit was added and incubated for 15 min on the ice. Then, the cells were homogenized and centrifuged for 10 min at 4 °C, 1000 × *g*. The supernatant was collected and centrifuged for 10 min at 4 °C, 3500 × *g*. After that, the pellet was collected as mitochondria and lysed using the mitochondrial lysis buffer provided by the kit. BCA assay was performed to determine the concentration of mitochondrial protein.

### Mitotracker

Cells were transfected with overexpression of SLC1A5_var and then incubated with Mitotracker Red solution (Concentration: 1 μM, Ex/Em =581 nm/664 nm) at 37 °C for 30 min. Then colocalization of Mitotracker (Thermo, M22425) and SLC1A5_var was captured by CarlZeiss LSM710 confocal microscope.

### Detection of ATP

The ATP content in astrocytes was detected using the Enhanced ATP Assay Kit (Beyotime Biotechnology, S0027) following manufacturer protocol.

### Mitochondrial membrane potential (MMP)

MMP was measured by the JC-1 probe (Invitrogen) to determine the mitochondrial membrane potential. Astrocytes were stained with JC-1 dye (Concentration: 5 μM; Red: Ex/Em = 535/590 nm; Green: Ex/Em = 485/530 nm) for 30 min at 37 °C. Then they were analyzed by flow cytometer (FACS Calibur, BD).

### Mitochondrial reactive oxygen species (ROS)

For mitochondrial ROS measurements, cells were incubated with Mitosox Red (Concentration: 5 μM, Ex/Em = 510/580 nm, Invitrogen) or H2DCFDA (Concentration: 5 μM, Ex/Em = 488/525 nm, Beyotime) for 30 min.

Cells were analyzed by confocal microscopy and the level of Mitochondrial ROS was evaluated by a flow cytometer (FACS Calibur, BD).

### Transmission electron microscopy (TEM) analysis

The procedure was described in our previous study [13]. Primary astrocytes are perfused with 2.5% glutaraldehyde. Specimens were fixed in 1% osmium tetroxide, stained in aqueous uranyl acetate, and then dehydrated and embedded in epoxy resin. Ultrathin sections were stained using lead citrate and examined with the transmission electron microscope. Healthy mitochondria have a clear structure of the membrane. The inner membrane was folded to form normal mitochondrial cristae including lamellar, tubular, and vesicular cristae. Damaged mitochondria harboring morphologies including the disappeared double membrane structure and mitochondrial cristae, and the blurred edges. Each group randomly selected 4-5 visual fields to be photographed, then the percentage of damaged mitochondria was calculated.

### Annexin V/PI analysis

Cells were performed by Annexin V/PI apoptosis detection kit (Vazyme, A211-02) according to the manufacturer’s manual. The apoptosis rate was measured by flow cytometer (FACS Calibur, BD).

### Western blot

The method was described as previously [6]. Cell and tissue lysates were subjected to electrophoresis through SDS-PAGE and transferred to PVDF membranes, and then blotted with antibodies. Then it was visualized by enhanced chemiluminescence Western Blotting System (Bio-Rad Laboratories, Shanghai, China). Quantification was performed by ImageJ software. The antibodies used were shown in Supplementary Table S2.

### Peptide-N-glycosidase F (PNGase F) treatment

The whole lysed fraction of astrocytes was carefully handled on ice. It was critical that the samples containing SLC1A5_var were neither boiled (or heated above 40 °C) or frozen prior to resolution by immunoblotting except incubation with PNGase F (NEB, Cat#P0704L) at 37 °C for 4 h with nondenatured conditions. Then protein concentration was measured by BCA assay. PNGase F is the most effective enzymatic method for removing almost all N-linked oligosaccharides from glycoproteins [14]. SLC1A5_var were detected with anti-SLC1A5 antibody that can recognize the SLC1A5_var after peptide-N-glycosidase F (PNGase F) treatment.

### Quantitative real-time PCR

Astrocytes were plated in 6-well plates and transfected with either a SLC1A5_var vector lentivirus (SLC1A5_var vec) or SLC1A5_var overexpression lentivirus (SLC1A5_var OE). And microglia were incubated in the serum-free medium and stimulated with LPS for 12 h. Then microglia-conditioned medium (MCM) was collected and centrifuged at 12,000 × *g* for 10 min at 4 °C and the supernatant was collected to stimulate the A1-type astrocytes. Total RNA of astrocytes was extracted as previously [13]. Briefly, astrocytes were extracted using TRIzol reagent (Invitrogen, Carlsbad, CA, USA) Then, Prime Script RT Master Mix (Vazyme, China) were used to get cDNA. Then it was followed by ChamQ SYBR qPCR Master Mix (Vazyme, China), according to the manufacturer’s instructions. β-Actin was used as the internal control for each sample. The sequences of primers for RT-PCR were listed in Supplementary Table S1.

### ELISA

The concentration of IL-1*β*, IL-6, and TNF-*α* in the serum and cell culture were detected by mouse IL-1*β*, IL-6, and TNF-*α* ELISA Kit (Excell, China) according to the manufacturer’s instructions. The concentration of IL-18 was detected by the mouse IL-18 ELISA Kit (R&D, DY7625-05).

### In situ hybridizations (ISH)

Tissue samples were fixed in neutral buffered formalin (NBF 10%) and sections were hybridized with mouse SLC1A5_var probes. It was performed using the RNA scope Duplex Reagent Kit (322430, Advanced Cell Diagnostics).

### Detection of glutamine by high-performance liquid chromatography (HPLC)

Take the whole brain of mice and separate the brain regions of the cortex, striatum, hippocampus, and midbrain. The samples were homogenized with 0.1 M perchloric acid (1 mg tissue in 100 μL perchloric acid), treated by ultrasonic and centrifuged at 20,000 rpm for 30 min at 4 °C. The supernatant was collected for testing. The detection system is HPLC-EDC (Thermo Fisher Scientific. USA). The mobile phase is Na_2_HPO_4_·12H_2_O (40 mM) and methyl alcohol with overall flow rate of 1 ml min^−1^. Samples were monitored by fluorescence detector at 328 nm excitation wavelength and 425 nm emission wavelength.

### Statistical analysis

Statistical analyses were performed using SPSS version 22.0 or GraphPad Prism 8.0 software, and all data were presented as mean ± S.E.M. Statistical significance was assessed with two-way ANOVA followed by Tukey’s post hoc test. *p* < 0.05 was predetermined as the threshold for statistical significance.

## Results

### Glutamine transporter SLC1A5_var is located in mitochondria of astrocytes and significantly downregulated in MPTP and LPS mouse models

**Fig. 1:**
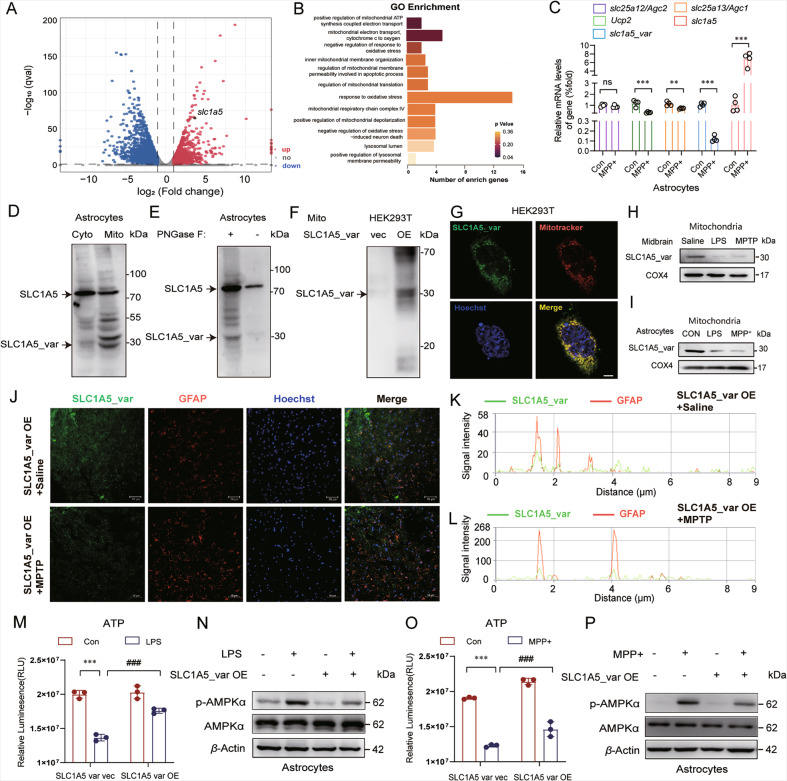
Mitochondria glutamine transporter SLC1A5_var is located in astrocytes and is closely related to energy metabolism. **A** The volcano map of differential genes was shown while astrocytes were stimulated with MPP^+^ (*n* = 3). **B** Gene enrichment analysis by Gene Ontology showing the enriched pathways in astrocytes treated with MPP^+^. **C** Validation of enriched differential genes by qRT-PCR (*n* = 4). **D** Expression of SLC1A5 and SLC1A5_var in mitochondria and cytoplasm of astrocytes was detected by immunoblotting. **E** Expression of SLC1A5_var was detected while treated with PNGase F in astrocytes. **F** Mitochondria were separated to detect the expression of SLC1A5_var in HEK-293T cells. **G** Immunofluorescence of SLC1A5_var (green) and Mitotracker (red) in HEK-293T cells. Scale bar, 50 μm. The protein levels of SLC1A5_var in the mitochondria of the midbrain in the MPTP /LPS mice model were detected by immunoblotting (**H**). The levels of SLC1A5_var in the mitochondria of astrocytes treated with MPP^+^ /LPS were detected by immunoblotting (**I**). **J** Colocalization and (**K**, **L**) statistics of SLC1A5_var and TH in the midbrain. Scale bar represents 50 μm. **M**, **O** Effect of SLC1A5_var on ATP generation in the presence or absence of LPS or MPP^+^(*n* = 3). **N**, **P** The level of p-AMPK while overexpression of SLC1A5_var in astrocytes stimulated with LPS (100 ng/ml) for 6 h or MPP^+^ (500 μM) for 24 h (*n* = 3). Data are shown as the mean ± S.E.M. ns, *P* > 0.05, **P < 0.01 and ***P < 0.001 using the Student’s *t* test (**C**, **J**–**M**). **P < 0.01 and ***P < 0.001 vs control, ^#^*P* < 0.05, ^###^*P* < 0.001 vs LPS group, two-way ANOVA followed by Tukey’s post hoc test (**N**, **P**, **Q**).

RNA sequencing was performed in astrocytes stimulated with or without MPP^+^ to gain PD-related genes. From the volcano plot, 1858 upregulated and 2561 downregulated genes were obtained in the context of MPP^+^-stimulation (Fig. 1A). In the volcano plot, we found the gene slc1a5 was significantly upregulated and it has been demonstrated closely correlated with the progression of PD in our previous study. Moreover, the differentially expressed genes associated with mitochondrial function and oxidative stress, two major causes of PD, were selected further and enriched using Gene Ontology (http://geneontology.org/). GO analysis enriched pathways was shown in Fig. 1B and validated by qRT-qPCR. Interestingly, we found slc1a5 was significantly increased consistently with RNA sequencing while the mitochondrial variant SLC1A5_var was significantly decreased (Fig. 1C). Therefore, the gene SLC1A5_var has aroused our interest and it was selected for further study. Recent evidence has shown that SLC1A5_var is a mitochondrial transporter of glutamine in cancer cells [11], while the function of SLC1A5_var in astrocytes and PD is completely unknown. To this end, we explored the expression pattern of SLC1A5 in the context of PD. Firstly, RNA scope showed the existence of SLC1A5_var in the midbrain of mice (Supplementary Fig. 1A). Then, the SLC1A5_var was found in the mitochondrial fractions of astrocytes (Fig. 1D) which is consistent with the finding in cancer cells [11]. Moreover, after treatment with peptide-N-glycosidase F (PNGase F), endogenous SLC1A5_var were detectable in the whole cell lysates of astrocytes using an anti-SLC1A5 antibody (Fig. 1E). To give a visualization of cellular location of SLC1A5_var, HEK-293T cells (much higher transfection efficiency than astrocytes) were used to transfect with Slc1a5_var (Supplementary Fig. S1B). Overexpression of SLC1A5_var was easily detected in the cell lysates (Fig. 1F) and clearly observed in the mitochondria of HEK-293T cells (Fig. 1G). Therefore, we confirmed that SLC1A5_var is located in the mitochondrion. More importantly, we found that SLC1A5_var was decreased significantly in both midbrain-and astrocytes-derived mitochondria upon LPS or MPTP/MPP^+^ exposure (Fig. 1H, I and Supplementary Fig. S1C–F), suggesting a potential role of SLC1A5_var in PD. Intriguingly, although overexpression of SLC1A5_var was observed in both astrocytes (marked by GFAP in Fig. 1J) and DA neurons (marked by TH in Supplementary Fig. S1G), dramatic decline of SLC1A5_var was mostly found in astrocytes (Fig. 1K, L) but not in DA neurons (Supplementary Fig. S1H–I), indicating astrocytic SLC1A5_var may play crucial roles in PD progression. Thus, we investigated the function of astrocytic SLC1A5_var in the following study.

Astrocytes transfected with SLC1A5_var plasmid exhibited elevated mRNA level of *SLC1A5_var* (Supplementary Fig. S2A) and increased protein level of mitochondrial SLC1A5_var (Supplementary Fig. S2B). Importantly, overexpression of SLC1A5_var rescued LPS-induced ATP depletion (Fig. 1M), and reversed AMPK activation reflected by decreased phosphorylation of AMPKα (Fig. 1N and Supplementary Fig. S2C) which acts as a sensory signal of cellular energy [15]. Similarly, the effect of SLC1A5_var overexpression on energy metabolism was replicable in MPP^+^-stimulated astrocytes (Fig. 1O, P and Supplementary Fig. S2D). These results suggest that the mitochondrial glutamine transporter SLC1A5_var is a key regulator of energy metabolism in astrocytes, and loss of SLC1A5_var may contribute to PD progression.

### SLC1A5_var ameliorates disrupted mitochondrial energy metabolism in LPS-treated astrocytes

**Fig. 2:**
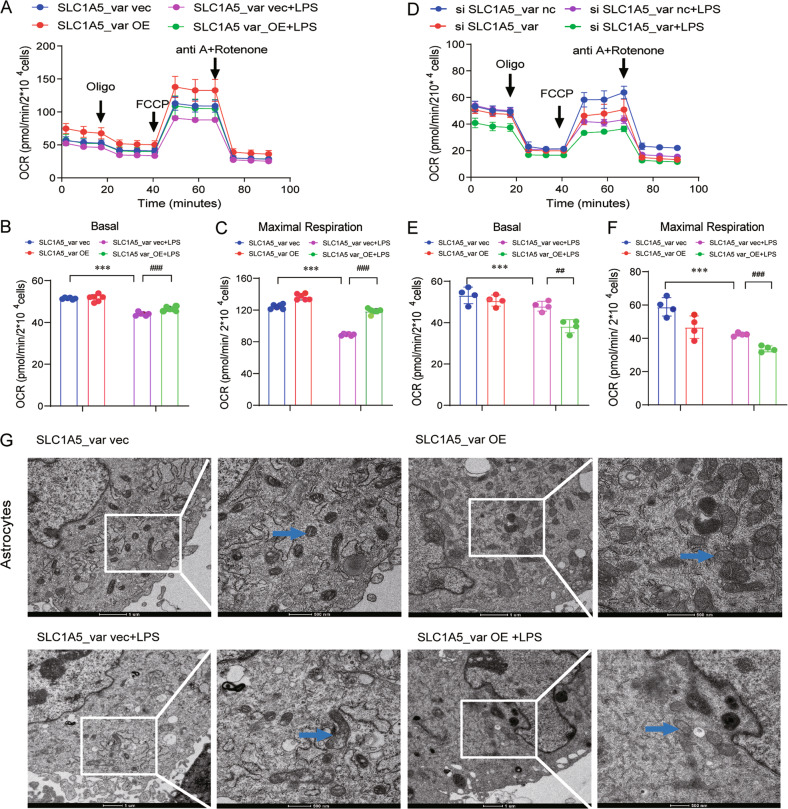
SLC1A5_var ameliorates LPS-induced dysfunction of mitochondrial in astrocytes. Astrocytes were transfected with SLC1A5_var for 48 h and stimulated with LPS (100 ng/ml) for 6 h. Effect of SLC1A5_var overexpression (**A**) (*n* = 6 from 6 mice/group) or knockdown (**D**) (*n* = 4 from 4 mice/group) on the oxygen consumption rate in astrocytes stimulated with LPS. Effect of SLC1A5_var overexpression (**B**) or knockdown (**E**) on basal respiration of astrocytes. Effect of SLC1A5_var overexpression (**C**) or knockdown (**F**) on maximum respiration from oxidative phosphorylation in astrocytes. The non-mitochondrial respiration was not subtracted for the quantification. Each data point represents a separate well of the seahorse plate, and the astrocytes in each well were isolated from different mice. *P < 0.05, **P < 0.01 and ***P < 0.001 vs control, ^##^*P* < 0.01 and ^###^*P* < 0.001 vs LPS group, two-way ANOVA followed by Tukey’s post hoc test. **G** The morphology of mitochondria is observed in transmission electron microscopic (TEM). Blue arrows: mitochondria. Scale bar, 1 μm (left), 500 nm (Enlarged vision).

To investigate the role of SLC1A5_var in mitochondrial energy metabolism, oxygen consumption rate (OCR) of astrocytes was traced by Seahorse XF analyzer. Overexpression of SLC1A5_var reversed LPS-induced reduction in basal oxygen consumption and maximal respiration (Fig. 2A–C). Meanwhile, reduced ATP production and proton leak were improved by SLC1A5_var overexpression in LPS-stimulated astrocytes (Supplementary Fig. S3A–C). To validate the effect of SLC1A5_var on mitochondrial energy metabolism, astrocytes were transfected with si*Slc1a5_var*. Transfection of si*Slc1a5_var* decreased the mRNA level of *Slc1a5_var* but not *Slc1a5* in astrocytes (Supplementary Fig. S3D, E), and the protein level of mitochondrial SLC1A5_var was reduced as well in si*Slc1a5_var*-transfected astrocytes (Supplementary Fig. S3F). Consistent with the findings in SLC1A5_var overexpressed astrocytes, knockdown of SLC1A5_var aggravated LPS-induced reduction in basal oxygen consumption and maximal respiration (Fig. 2D–F). Although no change in ATP production and non-mitochondrial oxygen consumption were observed, knockdown of SLC1A5_var further reduced the proton leak in astrocytes with LPS treatment (Supplementary Fig. S3G–I). These results highlight the importance of SLC1A5_var in mitochondrial energy metabolism.

Besides energy metabolism, we also found the beneficial effect of SLC1A5_var on mitochondrial integrity. The mitochondrial morphology was observed in astrocytes by transmission electron microscope (TEM). As shown in Fig. 2G and Supplementary Fig. S4A, LPS treatment led to the accumulation of damaged mitochondria with disappeared double membrane structure and mitochondrial cristae, and the blurred edges. Intriguingly, overexpression of SLC1A5_var reduced the amount of abnormal mitochondria in LPS-treated astrocytes. In contrast, knockdown of SLC1A5_var aggravated LPS-induced accumulation of injured mitochondria (Supplementary Fig. S4B, C). The above results reveal a potential role of SLC1A5_var in maintaining mitochondrial integrity which is critical for mitochondrial function.

### SLC1A5_var reduces overproduction of mitochondrial ROS and maintains mitochondrial membrane potential in the LPS model

**Fig. 3:**
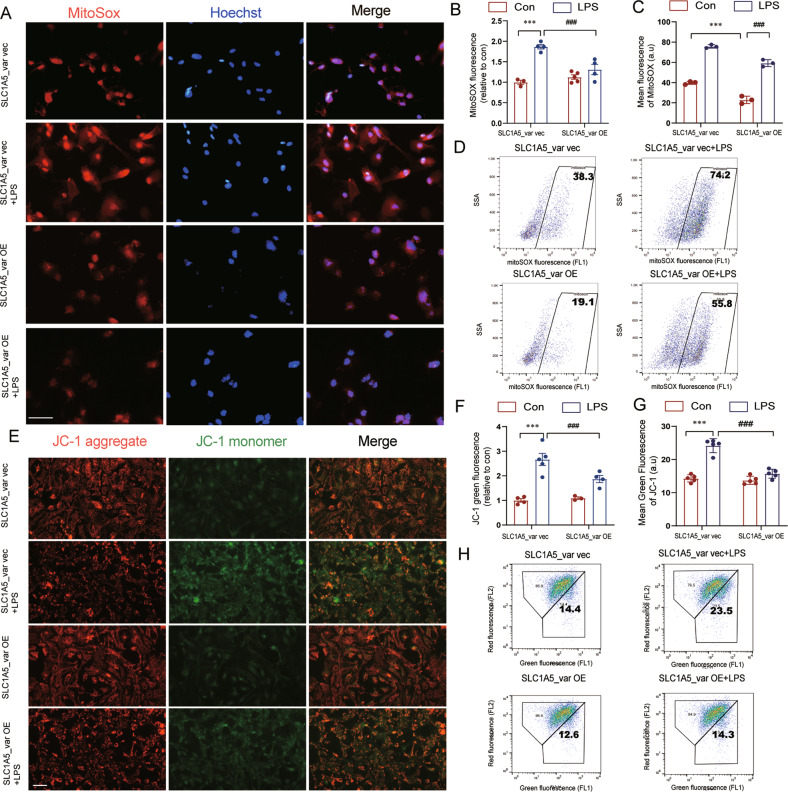
SLC1A5_var attenuates the level of the mitochondrial ROS and membrane potential induced by LPS. Mitochondrial ROS accumulation was measured by the MitoSOX assay and monitored by fluorescence microscopy (**A**, **B**) (*n* = 3–5) and flow cytometry (**C**, **D**) (*n* = 3). Scale bar: 40 μm. Mitochondrial membrane potential (MMP) was measured by the JC-1 assay (**E**, **F**) (*n* = 3–5) and monitored by flow cytometry (**H**) (*n* = 5). Scale bar: 50 μm. **G** The intensity of green fluorescence cells was analyzed. Data are shown as the mean ± S.E.M. ***P < 0.001 vs control, ^###^*P* < 0.001 vs LPS group, two-way ANOVA followed by Tukey’s post hoc test.

During the last decade, accumulating evidence suggested that mitochondrial dysfunction, ROS levels, and aberrations in mitochondrial morphology are interconnected [16]. To determine whether SLC1A5_var attenuates LPS-induced ROS production, the mitoSOX assay was performed. As shown in Fig. 3A, B, mitochondrial ROS (mito-ROS) was significantly increased upon LPS exposure in astrocytes, while overexpression of SLC1A5_var markedly suppressed LPS-induced mito-ROS production. From the flow cytometry data shown in Fig. 3C, D, LPS caused a significant increase (92.25 ± 1.78% relative to the SCL1A5_var vec group) in mitoSOX-positive astrocytes, and overexpression of SLC1A5_var reduced the amount of mitoSOX-positive astrocytes (22.65 ± 2.25% relative to the SCL1A5_var vec+LPS group). Meanwhile, overexpression of SLC1A5_var promoted the scavenging of intracellular ROS indicated by DCFH-DA probe in the LPS-treated astrocytes (Supplementary Fig. S5A–C). On the contrary, the knockdown of SLC1A5_var aggravated ROS production (Supplementary Fig. S5D, E). As LPS treatment and overexpression of SLC1A5_var showed no effects on apoptosis of astrocytes and C8-D1A cells (Supplementary Fig. S6A, B), the effect of SLC1A5_var on ROS scavenging should not be attributed to cell death-caused ROS reduction. Besides, the markedly reversed mitochondrial membrane potential (MMP) induced by LPS (80.23 ± 1.54% relative to the SCL1A5_var vec group) was visualized by the JC-1 green fluorescence, and it was significantly decreased (40.18 ± 1.10% relative to the SCL1A5_var vec +LPS group) after overexpression of SLC1A5_var (Fig. 3E–H). Together, these data demonstrate that SLC1A5_var attenuates LPS-induced mitochondrial ROS accumulation and low MMP.

### SLC1A5_var inhibits activation of NF-κB signaling in LPS-stimulated astrocytes

**Fig. 4:**
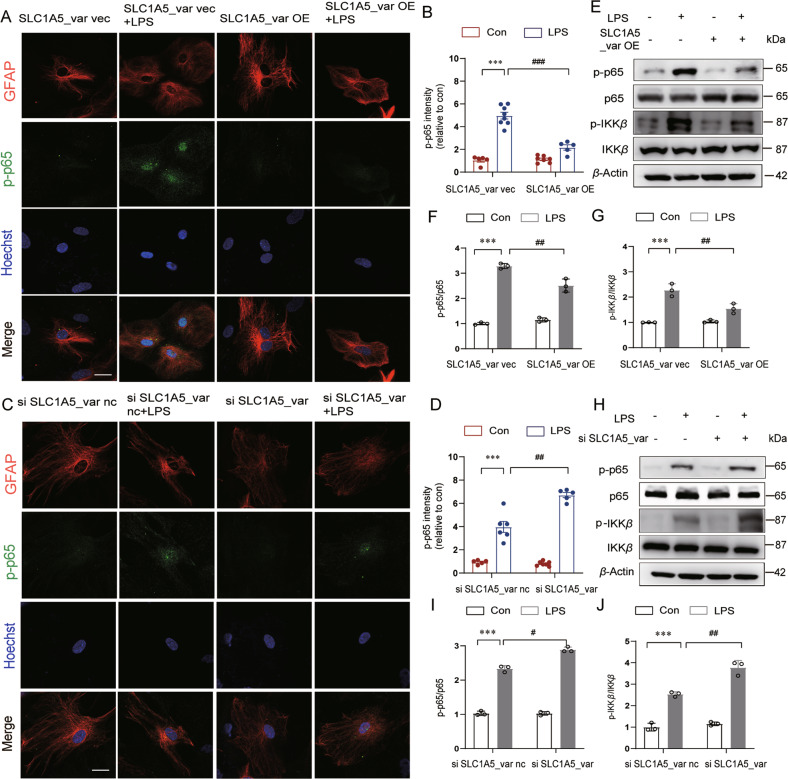
SLC1A5_var significantly blocks the nuclear translocation of p-p65 in LPS-stimulated astrocytes. Astrocytes were transfected with SLC1A5_var OE (**A**, **B**) or SLC1A5_var siRNA (**C**, **D**) for 48 h and then stimulated with LPS for 6 h. Phosphorylation of nuclear translocation of NF-κB (p65) was assessed by fluorescence microscopy (*n* = 5–8). Scale bar represents 20 μm. Phosphorylation of IKK*β* and p65 was determined by western blot analysis (**E**, **H**) and quantified by densitometry (**F**, **G** and **I**, **J**). Data are shown as the mean ± S.E.M. ***P < 0.001 vs control, ^#^*P* < 0.05, ^##^*P* < 0.01 and ^###^*P* < 0.001 vs LPS group, two-way ANOVA followed by Tukey’s post hoc test.

Next, we explored the downstream effects of SLC1A5_var-mediated mitochondrial function in LPS-treated astrocytes. Accumulation of mitochondrial ROS triggers activation of NF-κB signaling which is a critical regulatory mechanism involved in neuroinflammation [17, 18]. We then examined the phosphorylation and nuclear translocation of p65 in astrocytes with LPS treatment. Our data showed that overexpression of SLC1A5_var reduced (Fig. 4A, B) but knockdown of SLC1A5_var aggravated (Fig. 4C, D) LPS-induced phosphorylation and nuclear translocation of p65 in astrocytes. Thereafter, changes of NF-κB signaling molecules including p65/phospho-p65 (p-p65) and IKKβ/ phospho-IKKβ (p-IKKβ) were measured by Western blots. LPS stimulation elevated phosphorylation levels of both p65 and IKKβ, activating NF-κB signaling, while overexpression of of SLC1A5_var attenuated this signaling by reducing p-p65 and p-IKKβ levels (Fig. 4E–G). Consistently, knockdown of SLC1A5_var enhanced activation of NF-κB signaling (Fig. 4H–J). Therefore, SLC1A5_var acts as negative regulator of NF-κB activation.

### SLC1A5_var reduces A1 astrocyte reactivity in the LPS model

**Fig. 5:**
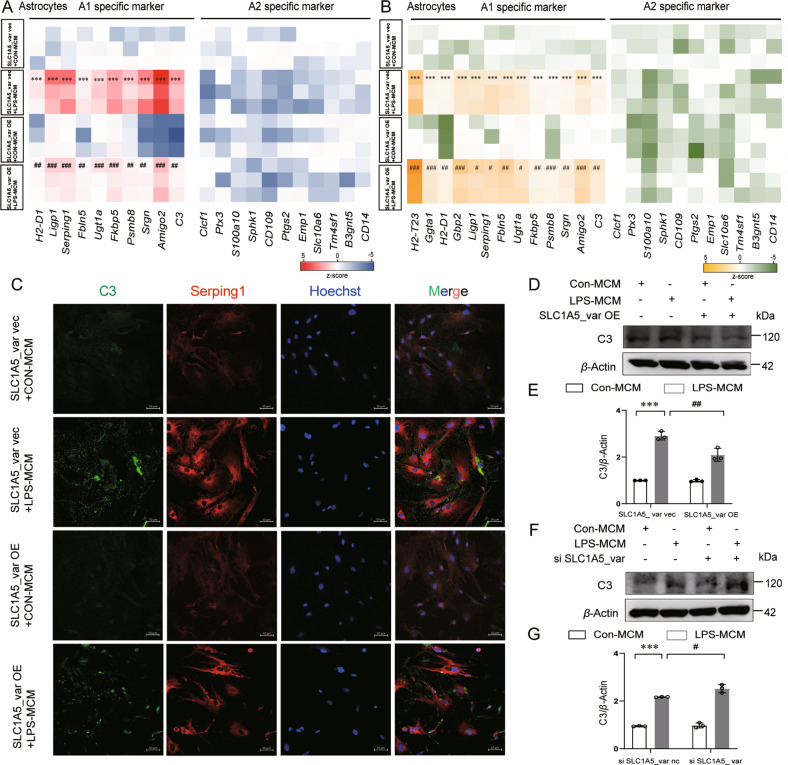
SLC1A5_var decreases A1 astrocyte reactivity in the LPS-stimulated model. Astrocytes were transfected with SLC1A5_var OE or siRNA for 48 h and then replaced with the conditioned medium of microglia stimulated with LPS for 6 h. Then cell lysate was analyzed by QT-PCR. Heatmap of A1-specific and A2-specific markers in astrocytes while SLC1A5var was overexpression (**A**) (*n* = 3) or knockdown (**B**) (*n* = 3). **C** Colocalization of C3 (green) and Serping1(red) while SLC1A5_var was overexpressed. Scale bar, 50 μm. Expression of C3 was determined by Western blot while SLC1A5_var was overexpression (**D**) or knockdown (**F**) and quantified by densitometry (**E**, **G**) (*n* = 3). Data are shown as the mean ± S.E.M. ***P < 0.001 vs control, ^#^*P* < 0.05, ^##^*P* < 0.01 vs LPS group^,^ two-way ANOVA followed by Tukey’s post hoc test.

As is known, increased intracellular ROS production activates microglia to secrete pro-inflammatory cytokines and exacerbate inflammation [19]. A1 reactive astrocytes can be induced by neuroinflammatory microglia [20]. Since we had demonstrated that SLC1A5_var attenuates the level of ROS stimulated by LPS, Next, we determined whether SLC1A5_var had an effect on the astrocyte reactivity by assessing mRNA levels of A1- and A2-specific markers. The expression of A1-specific markers (*H2-D1*, *Ligp1*, *Serping1*, C3, *Fbln5*, *Fkbp5*, *Psmb8*, *Amigo2*, and *Ugt1a*) were increased by LPS stimulation, which was reversed by overexpression of SLC1A5_var (Fig. 5A). However, LPS stimulation failed to affect the levels of A2-specific markers (*Clcf1*, *Ptx3*, *S100a10*, *Sphk1*, *Emp1*, *Slc10a6*, *Tm4sf1*, *Cd109*, *Ptgs2*, *B3gnt5* and *Cd14*) in astrocytes (Fig. 5A, *P* > 0.05). Meanwhile, knockdown of SLC1A5_var significantly enhanced LPS-stimulated upregulation of A1- but not A2-specific markers (Fig. 5B). As C3 and Serping1 are the characteristic markers of A1 astrocytes, we observed that overexpression of SLC1A5_var decreased the levels of C3 and Serping1 elevated by LPS treatment using immunofluorescence staining (Fig. 5C) and Western blot (Fig. 5D, E). In contrast, knockdown of SLC1A5_var boosted expression of C3 and Serping1 in LPS-treated astrocytes (Fig. 5F, G and Supplementary Fig. S7A). These results uncover the beneficial effect of SLC1A5_var on astrocyte phenotype polarization.

### SLC1A5_var inhibits the release of astrocytic pro-inflammatory cytokines through blunting TLR4-mediated signaling in the LPS model

**Fig. 6:**
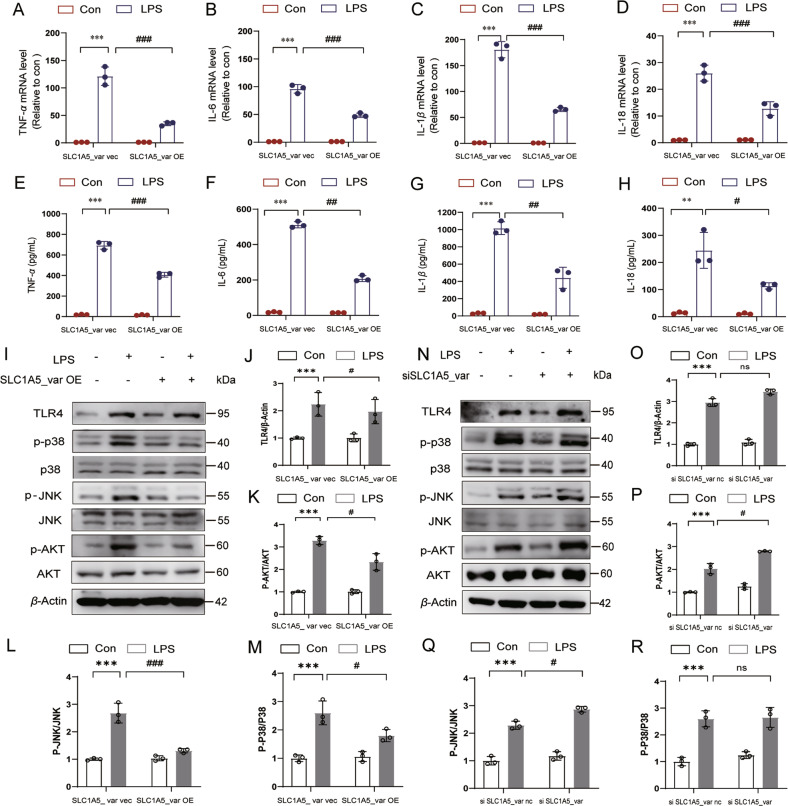
SLC1A5_var inhibited LPS-induced astrocytic inflammatory cytokines and down-regulates TLR4 signal pathway. Astrocytes were transfected with SLC1A5_var (OE) or with SLC1A5_var siRNA for 48 h and stimulated with LPS (100 ng/ml) for 6 h. The mRNA expression levels of *Tnf-α* (**A**), *Il-6* (**B**), *Il-1β* (**C**), and *Il-18* (**D**) stimulated with LPS were detected by qRT-qPCR(*n* = 3). Secretion of TNF-*α* (**E**), IL-6 (**F**), IL-1*β* (**G**) and IL-18 (**H**) in astrocytes stimulated with LPS was detected by ELISA (*n* = 3). The protein level of TLR4, p-p38, p-JNK and p-AKT was determined by western blot (**C**, **F**) while SLC1A5_var was overexpression (**I**) or knockdown (**N**) and quantified by densitometry (**J**–**M** and **O**–**R**) (*n* = 3). Data are shown as the mean ± S.E.M. **P < 0.01, ***P < 0.001 vs control, ns, *P* > 0.05, ^#^*P* < 0.05, ^##^*P* < 0.01 and ^###^*P* < 0.001 vs LPS group, two-way ANOVA followed by Tukey’s post hoc test.

As A1 is the pro-inflammatory astrocyte phenotype, we next examined levels of pro-inflammatory cytokines in LPS-stimulated astrocytes. Pro-inflammatory cytokines including TNF-*α*, IL-6, IL-1*β*, and IL-18 were significantly increased in mRNA levels (Fig. 6A–D) and proteins levels (Fig. 6E–H) upon LPS exposure, while overexpression of SLC1A5_var reduced upregulation of these pro-inflammatory cytokines. Then, we sought to explore the mechanism underlying SLC1A5_var-mediated inhibition of inflammation. Toll-like receptor 4 (TLR4) recognizes LPS and activates NF-κB signaling to enhance inflammatory cytokine production [21]. Our data showed that overexpression of SLC1A5_var inhibited LPS-induced upregulation of TLR4, p-p38, p-JNK, and p-AKT (Fig. 6I–M). Besides, knockdown of SLC1A5_var increased the expression of TLR4, p-p38, p-JNK, and p-AKT (Fig. 6N–R). Therefore, these data suggest that the anti-inflammatory activity of SLC1A5_var is attributed to the inhibition of TLR4-mediated signal transduction.

### SLC1A5_var alleviates neuroinflammation and attenuates dopaminergic neurons loss in the MPTP and LPS mouse models

**Fig. 7:**
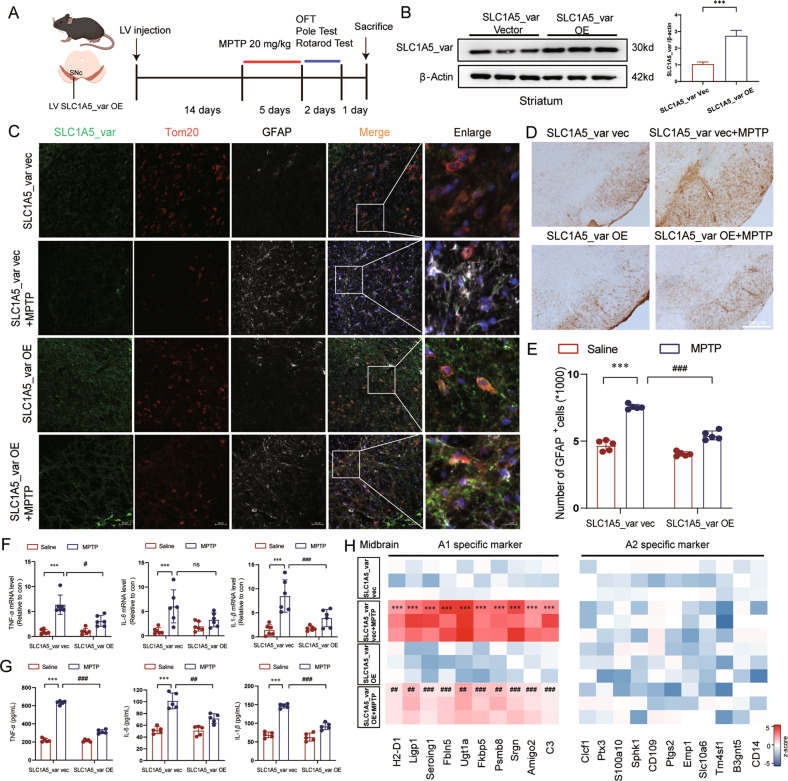
SLC1A5_var alleviates the neuroinflammation in the MPTP mice model. **A** Schematic diagram of the experimental procedure in the sub-MPTP mouse model. **B** The efficiency of SLC1A5_var after LV injection in the midbrain was examined by immunoblotting (*n* = 3). Data are shown as the mean ± S.E.M. ***P < 0.001 using the Student’s *t* test. **C** Immunofluorescence staining of SLC1A5_var (green) and Tom20 (red) and GFAP (white) in the midbrain. Scale bars, 50 μm. Immunohistochemical images of GFAP-positive astrocytes in SNpc (**D**) and quantification (**E**) (*n* = 5 mice/group). Scale bar, 200 μm. **F** Effects of overexpression of SLC1A5_var on the mRNA level of *Tnf-α, Il-1β*, and *Il-6* in the midbrain by qPCR (*n* = 6). **G** Effects of overexpression of SLC1A5_var on the secretion of TNF-*α*, IL-6, and IL-1*β* in the midbrain by ELISA (*n* = 5). **H** Heatmap of genes represents A1-specific and A2-specific markers in the midbrain after SLC1A5_var LV injection analyzed by qPCR (*n* = 5). Data are shown as the mean ± S.E.M. ***P < 0.001 vs control, ns, *P* > 0.05, ^#^*P* < 0.05, ^##^*P* < 0.01 and ^###^*P* < 0.001 vs MPTP group, two-way ANOVA followed by Tukey’s post hoc test.

Both loss of dopaminergic neurons in the SNpc and enhanced neuroinflammation are involved in the progression of PD [22, 23]. To evaluate the neuroprotective role of SLC1A5_var in PD, we conducted the MPTP mouse model. Briefly, C57BL/6 mice were microinjected with SLC1A5_var LV in the SNpc and received MPTP treatment (20 mg/kg) for 5 days afterward the LV injection for 2 weeks (Fig. 7A). Successful overexpression of SLC1A5_var was validated by Western blot using striatum samples (Fig. 7B). Overexpression of SLC1A5_var mainly distributed in mitochondria of astrocytes, and inhibited astrocyte activation and proliferation in the midbrain of MPTP-treated mice (Fig. 7C–E). Importantly, overproduction of pro-inflammatory cytokines including TNF-*α*, IL-6, IL-1*β*, and IL-18 were significantly reduced by SLC1A5_var overexpression (Fig. 7F, G), which was attributed to the inhibition of astrocyte phenotype polarization to A1 reactive astrocytes (Fig. 7H). Together, these results strengthen the anti-inflammatory effect of SLC1A5_var.

**Fig. 8:**
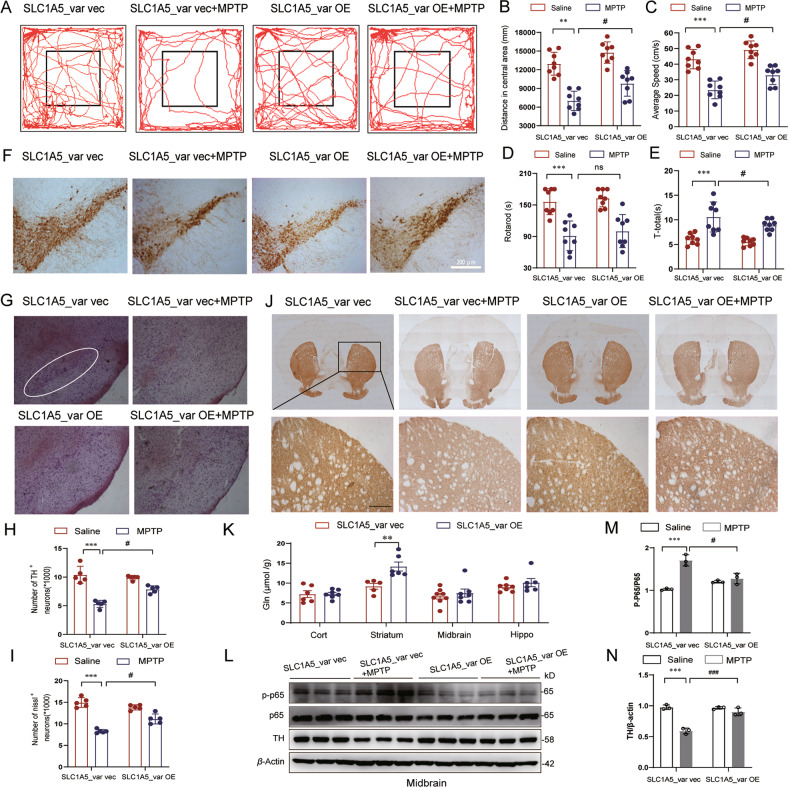
SLC1A5_var exhibited neuroprotective effects in the MPTP-induced PD mice model. Moving routes (**A**), distance (**B**), and speed (**C**) of mice in the open field in 5 min. **D** Total climbing time in the pole test. **E** Latency to fall in the rotarod test within 180 s. **A**–**E** (*n* = 8 mice/group). Immunohistochemical images of TH^+^ neurons (**F**) and nissl^+^ neurons (**G**) in SNpc with quantification (**H**, **I**) (*n* = 5 mice/group). Scale bars, 200 μm. **J** Immunohistochemical images of TH^+^ neurons in the striatum. Scale bars, 80 μm. **K** The content of Gln in different brain regions of mice was analyzed by HPLC (*n* = 6 mice/group). Data are shown as the mean ± S.E.M. **P < 0.01 using the Student’s *t* test. **L**–**N** Protein levels of TH, p-p65, and p65 in the midbrain were analyzed by western blot (**L**) and quantified (**M**, **N**) (*n* = 3). Data are shown as the mean ± S.E.M. **P < 0.01, ***P < 0.001 vs control, ns, *P* > 0.05, ^#^*P* < 0.05, ^##^*P* < 0.01 and ^###^*P* < 0.001 vs MPT*P* group, two-way ANOVA followed by Tukey’s post hoc test.

To evaluate the therapeutic effect of SLC1A5_var on PD, we performed behavioral analyses of PD-like motor symptoms. In the open field test, MPTP-treated mice exhibited shorter travel distance in central area and slower speed relative to saline-treated mice, while overexpression of SLC1A5_var improved the locomotor activity (Fig. 8A–C). Motor coordination was evaluated by rotarod test and pole test. Overexpression of SLC1A5_var had no effect on rotarod test but improved the performance in pole test (Fig. 8D, E). Overall, SLC1A5_var facilitates the improvement of motor symptoms in PD mice. More importantly, loss of dopaminergic neurons in the SNpc and striatum were rescued by SLC1A5_var overexpression (Fig. 8F–J). As SLC1A5_var is a mitochondrial glutamine transporter, we measured the glutamine content by HPLC. Glutamine was increased significantly in the striatum of mice with SLC1A5_var overexpression (Fig. 8K). The expression of p65/p-p65 and TH was examined by Western blot. Upregulation of p-p65 and downregulation of TH in the midbrain of MPTP-treated mice were reversed by SLC1A5_var overexpression (Fig. 8L–N). Hence, SLC1A5_var exerts a neuroprotective effect in the MPTP mouse model.

Next, we confirmed the therapeutic effect of SLC1A5_var on PD using LPS-induced neuroinflammation model which is also widely used to replicate PD phenotype [24]. In the LPS mouse model, overexpression of SLC1A5_var failed to improve the locomotor activity evaluated by open filed test (Supplementary Fig. S8A, B), and rotarod test was not sensitive enough to examine motor coordination in this context (Supplementary Fig. S8C). However, the defect of motor coordination of LPS-treated mice was observed in pole test, and attenuated by SLC1A5_var overexpression (Supplementary Fig. S8D). Meanwhile, from the HPLC analysis, the reduced dopamine level was reversed by SLC1A5_var overexpression (Supplementary Fig. S8E). Consistent with the findings in the MPTP model, overexpression of SLC1A5_var also rescued loss of dopaminergic neurons in the LPS model (Supplementary Fig. S8F, G). Overall, these data suggest that SLC1A5_var elicits the neuroprotective effect via alleviating the neuroinflammation in the LPS-induced PD model.

## Discussion

Mitochondrial dysfunction and oxidative stress have been recognized as major contributors to the death of DA neurons in PD. Although it has been increasingly clear that mitochondrial dysfunction in astrocytes triggers neuroinflammation underlying the development of PD [25, 26], sophisticated regulation of mitochondrial function within astrocytes remains largely unknown. In this study, by replicating mitochondrial dysfunction and oxidative stress in astrocytes, we identified that SLC1A5_var is required for mitochondrial energy metabolism. More importantly, genetic manipulation of SLC1A5_var further validated its beneficial effects on PD pathology.

SLC1A5 has been widely studied as a neutral amino acid transporter located in the plasma membrane of most mammalian cells [27], while intriguingly a novel variant of SLC1A5 (SLC1A5_var) was discovered recently [11]. The SLC1A5_var contains an N-terminal targeting signal for mitochondrial localization by which SLC1A5_var mediates mitochondrial glutamine metabolism in cancer [11]. Consistently, SLC1A5_var also acts as a mitochondrial glutamine transporter in astrocytes demonstrated by our findings. To investigate the role of SLC1A5_var in PD progression, both MPTP/MPP^+^ and LPS models were performed by in vivo and in vitro experiments. The expression of SLC1A5_var was decreased significantly in mitochondria of astrocytes reflected by both of the two PD models. Thereafter, we studied the effect of SLC1A5_var on mitochondrial energy metabolism by overexpression and knockdown of SLC1A5_var in astrocytes. Our data prove that SLC1A5_var preserves mitochondrial oxidative phosphorylation and reduces ROS production thus maintaining mitochondrial morphology and function in astrocytes treated with LPS. Simultaneously, we observed that genetic manipulation of SLC1A5_var expression showed no impact on the baseline parameters. The phenomenon that overexpression or knockdown of certain gene does not affect baseline parameters exists not only in our current study but also in other literatures [28, 29], which may indicate a disease-associated function of this gene.

Mitochondrial dysfunction-driven neuroinflammation has been testified as the major accelerator of the development of PD [25, 30]. To explore the regulatory role of SLC1A5_var in astrocyte-derived neuroinflammation, LPS model was performed to examine astrocyte reactivity and neuroinflammation-associated signaling pathway in the context of overexpression and knockdown of SLC1A5_var. Intriguingly, we found that SLC1A5_var inhibits astrocyte polarization from resting state into pro-inflammatory A1 phenotype but not anti-inflammatory A2 phenotype. Mechanistically, SLC1A5_var inactivates TLR4-mediated p38/JNK/AKT signaling pathway thus reducing the production and release of pro-inflammatory cytokines in response to LPS treatment.

Owing to the powerful enhancement of mitochondrial function and inhibition of neuroinflammation, we explored the effect of SLC1A5_var on PD pathology replicated by both MPTP and LPS mouse models. As expected, overexpression of SLC1A5_var significantly improves PD motor symptoms and rescues the loss of DA neurons in the midbrain and striatum regions, suggesting potential therapeutic effect of SLC1A5_var on PD. Further experiments are warranted to gain a comprehensive view of SLC1A5_var-driven mitochondrial transport of other neutral amino acids excluding glutamine. Additionally, cell type-specific effect of SLC1A5_var should be explored in future experiments. Nevertheless, this is the first study of the function of SLC1A5_var in PD which makes substantial noteworthy contribution to mitochondria-targeting strategy for PD therapy.

## Supplementary information

authorship form

Supplement material

aj-checklist

SLC1A5 var WB gel

## Data Availability

The raw data supporting the conclusions of this article will be made available by the authors.
