# Supplementary material for: Mitochondrial glutamine transporter SLC1A5_var, a potential target to suppress astrocyte reactivity in Parkinson’s Disease
Source: Cell Death Dis. 2022 Nov 9;13(11):946. doi: 10.1038/s41419-022-05399-z (PMC9646772; doi:10.1038/s41419-022-05399-z)
Supplement: Supplementary file 1 — authorship form [file 41419_2022_5399_MOESM1_ESM.pdf]

SPRINGER NATURE

Change of authorship request form - Journals (pre-acceptance)

Important information. Please read.

- This form should be used by authors to request any change in authorship (adding/deleting authors) including changes in corresponding authors. This form should not be used for name changes. Please fully complete all sections. Use black ink and block capitals and provide each author's full name with the given name first followed by the family name.

- By signing this declaration, all authors guarantee that the order of the authors are in accordance with their scientific contribution, if applicable as different conventions apply per discipline, and that only authors have been added who made a meaningful contribution to the work.

- Please note, in author collaborations where there is formal agreement for representing the collaboration, it is sufficient for the representative or legal guarantor (usually the corresponding author) to complete and sign the Authorship Change Form on behalf of all authors, **next to the added/removed author(s)**. **(Complete Section 3, followed by Section 6.)**
  In author collaborations where there is no formal agreement for representing the collaboration and there **are more than 10 authors**, one may sign for all, provided the signer appends correspondence that attests that each of the authors have agreed to the change **and** the **added/removed authors sign** the form. **(Complete Section 3, followed by Section 6.)**

- Please note, we cannot investigate or mediate any authorship disputes. If you are unable to obtain agreement from all authors (including those who you wish to be removed) you must refer the matter to your institution(s) for investigation. Please inform us if you need to do this.

- If you are not able to return a fully completed form within **30 days** of the date that it was sent to the author requesting the change, we may have to withdraw your manuscript. We cannot publish manuscripts where authorship has not been agreed by all authors (including those who have been removed).

- Incomplete forms will be rejected.

- Please return/upload this form, fully completed, to the Journals Editorial Office. The Journal and/or Publisher will consider the information you have provided to decide whether to approve the proposed change in authorship. We may decide to contact your institution for more information or undertake a further investigation, if appropriate, before making a final decision.

SPRINGER NATURE Springer Nature is one of the world's leading global research, educational and professional publishers, created in May 2015 through the combination of Nature Publishing Group, Palgrave Macmillan, Macmillan Education and Springer Science+Business Media.

SPRINGER NATURE

Change of authorship request form - Journals (pre-acceptance)

Section 1: Please provide the current title of manuscript

Manuscript ID no.: CDDIS-22-2151RRR

Title: Mitochondrial glutamine transporter SLC1A5_var, a potential target to suppress astrocyte reactivity in Parkinson's Disease

Section 2: Please provide the previous authorship, in the order shown on the manuscript before the changes were introduced. Please indicate the corresponding author by adding (CA) behind the name.

	First name(s)	Family name	ORCID or SCOPUS id, if available
1st author	YANG	LIU	
2nd author	YU TING	SONG	
3rd author	TING	LIU	
4th author	JIAN HUA	DING	
5th author	GANG	HU	
6th author	MING	LU	0000-0001-8018-8389

Please use an additional sheet if there are more than 10 authors.

SPRINGER NATURE Springer Nature is one of the world's leading global research, educational and professional publishers, created in May 2015 through the combination of Nature Publishing Group, Palgrave Macmillan, Macmillan Education and Springer Science+Business Media.

SPRINGER NATURE

Change of authorship request form - Journals (pre-acceptance)

Section 3: Please provide a justification for change. Please use this section to explain your reasons for changing the authorship of your manuscript, e.g. what necessitated the change in authorship? Please refer to the (journal) policy pages for more information about authorship. Please explain why omitted authors were not originally included and/or why authors were removed on the submitted manuscript.

In the three round of revision (CDDIS-22-2151R, CDDIS-22-2151RR,CDDIS-22-2151RRR), Lei Cao and Zhengwei Kang contributed to the work. Especially Lei Cao revised the manuscript and performed the additional experiments suggested from three refree, which greatly improved the quality of our paper. Zhengwei Kang involved in the primary cell culture and further immnofluorescence and immunohistochemistry detection. All authors agreed to add Lei Cao and Zhengwei Kang as co-author.

Section 4: Proposed new authorship. Please provide your new authorship list in the order you would like it to appear on the manuscript. Please indicate the corresponding author by adding (CA) behind the name. <u>If the Corresponding Author has changed, please indicate the reason under section 3</u>.

	First name(s)	Family name (this name will appear in full on the final publication and will be searchable in various abstract and indexing databases)	Affiliated institute	E-mail address
1st author	YANG	LIU	Nanjing University of Chinese Medicine	liuyang@njucm.edu.cn
2nd author	LEI	CAO	Nanjing Medical University	leicao@njmu.edu.cn
3rd author	YU TING	SONG	Nanjing University of Chinese Medicine	380225314@qq.com
4th author	ZHENG WEI	KANG	Nanjing Medical University	1724596679@qq.com
5th author	TING	LIU	Nanjing Medical University	njmu18601579691@163.com
6th author	JIAN HUA	DING	Nanjing Medical University	l15366506646@163.com
7th author	GANG	HU	Nanjing University of Chinese Medicine	neuropha@njmu.edu.cn
8th author	MING	LU	Nanjing Medical University	lum@njmu.edu.cn

Please use an additional sheet if there are more than 10 authors.

SPRINGER NATURE Springer Nature is one of the world's leading global research, educational and professional publishers, created in May 2015 through the combination of Nature Publishing Group, Palgrave Macmillan, Macmillan Education and Springer Science+Business Media.

SPRINGER NATURE

Change of authorship request form - Journals (pre-acceptance)

Section 5: Author contribution, Acknowledgement and Disclosures. Please use this section to provide a new disclosure statement and, if appropriate, acknowledge any contributors who have been removed as authors and ensure you state what contribution any new authors made (if applicable per the journal or book (series) policy). Please ensure these are updated in your manuscript - after approval of the change(s) - as our production department will not transfer the information in this form to your manuscript.

New acknowledgements:

Not applicable

New Disclosures (financial and non-financial interests, funding):

Not applicable

New Author Contributions statement (if applicable per the journal policy):

All authors read and approved the final manuscript. Yang Liu and Lei Cao designed and performed the experiments, analyzed the data, and wrote the manuscript. Yuting Song, Zhengwei Kang and Ting Liu performed experiments and analyzed the data. Jianhua Ding provides technical support. Gang Hu and Ming Lu conceived the study concept and designed the experiments. Lei Cao revised the manuscript. All authors discussed and contributed to the final manuscript.

State 'Not applicable' if there are no new authors.

SPRINGER NATURE

Change of authorship request form - Journals (pre-acceptance)

Section 6: Declaration of agreement. *All* authors, unchanged, new and removed *must* sign this declaration.
(NB: Please print the form, (docu)-sign and return/upload a scanned copy. Please note that signatures that have been inserted as an image file are acceptable as long as it is handwritten. Typed names in the signature box are unacceptable.) *** Please delete as appropriate. Delete all of the bold if you were on the original authorship list and are remaining as an author.**

	First name	Family name		Signature	Date
1st author	YANG	LIU	I agree to the proposed new authorship shown in section 4 and the proposed change in corresponding author	Yang Liu	2022-11-02
2nd author	LEI	CAO	I agree to the proposed new authorship shown in section 4 /and **the addition of my name to the authorship list** and the proposed change in corresponding author	Lei Cao	2022-11-02
3rd author	YU TING	SONG	I agree to the proposed new authorship shown in section 4 and the proposed change in corresponding author	Yuting Song	2022-11-02
4th authors	ZHENG WEI	KANG	I agree to the proposed new authorship shown in section 4 **/and the addition of my name to the authorship list** and the proposed change in corresponding author	Zheng Wei Kang	2022-11-02
5th author	TING	LIU	I agree to the proposed new authorship shown in section 4 and the proposed change in corresponding author	Ting Liu	2022-11-02
6th author	JIAN HUA	DING	I agree to the proposed new authorship shown in section 4 and the proposed change in corresponding author	Jianhua Ding	2022-11-02
7th author	GANG	HU	I agree to the proposed new authorship shown in section 4 and the proposed change in corresponding author	Gang Hu	2022.11.2

SPRINGER NATURE Springer Nature is one of the world's leading global research, educational and professional publishers, created in May 2015 through the combination of Nature Publishing Group, Palgrave Macmillan, Macmillan Education and Springer Science+Business Media.

SPRINGER NATURE

Change of authorship request form - Journals (pre-acceptance)

	First name	Family name		Signature	Date
8th author	MING	LU	I agree to the proposed new authorship shown in section 4 and the proposed change in corresponding author	[signature]	Nov. 2, 2022

In case of author collaborations with formal agreement:

	Name of consortium/consortia	First name	Family name		Signature	Date
Representative/legal guarantor				I agree to the proposed new authorship shown in section 4 /**and the addition/removal*of my name to the authorship list** /and the proposed change in corresponding author		

Both added/removed authors should complete the information in the first table under Section 6.

---- End of form ----

SPRINGER NATURE Springer Nature is one of the world's leading global research, educational and professional publishers, created in May 2015 through the combination of Nature Publishing Group, Palgrave Macmillan, Macmillan Education and Springer Science+Business Media.
