# Supplementary material for: Mitochondrial glutamine transporter SLC1A5_var, a potential target to suppress astrocyte reactivity in Parkinson’s Disease
Source: Cell Death Dis. 2022 Nov 9;13(11):946. doi: 10.1038/s41419-022-05399-z (PMC9646772; doi:10.1038/s41419-022-05399-z)
Supplement: Supplementary file 2 — Supplement material [file 41419_2022_5399_MOESM2_ESM.docx]

**Mitochondrial glutamine transporter SLC1A5_var, a potential target to suppress astrocyte reactivity in Parkinson’s Disease**

**Supplementary figures**


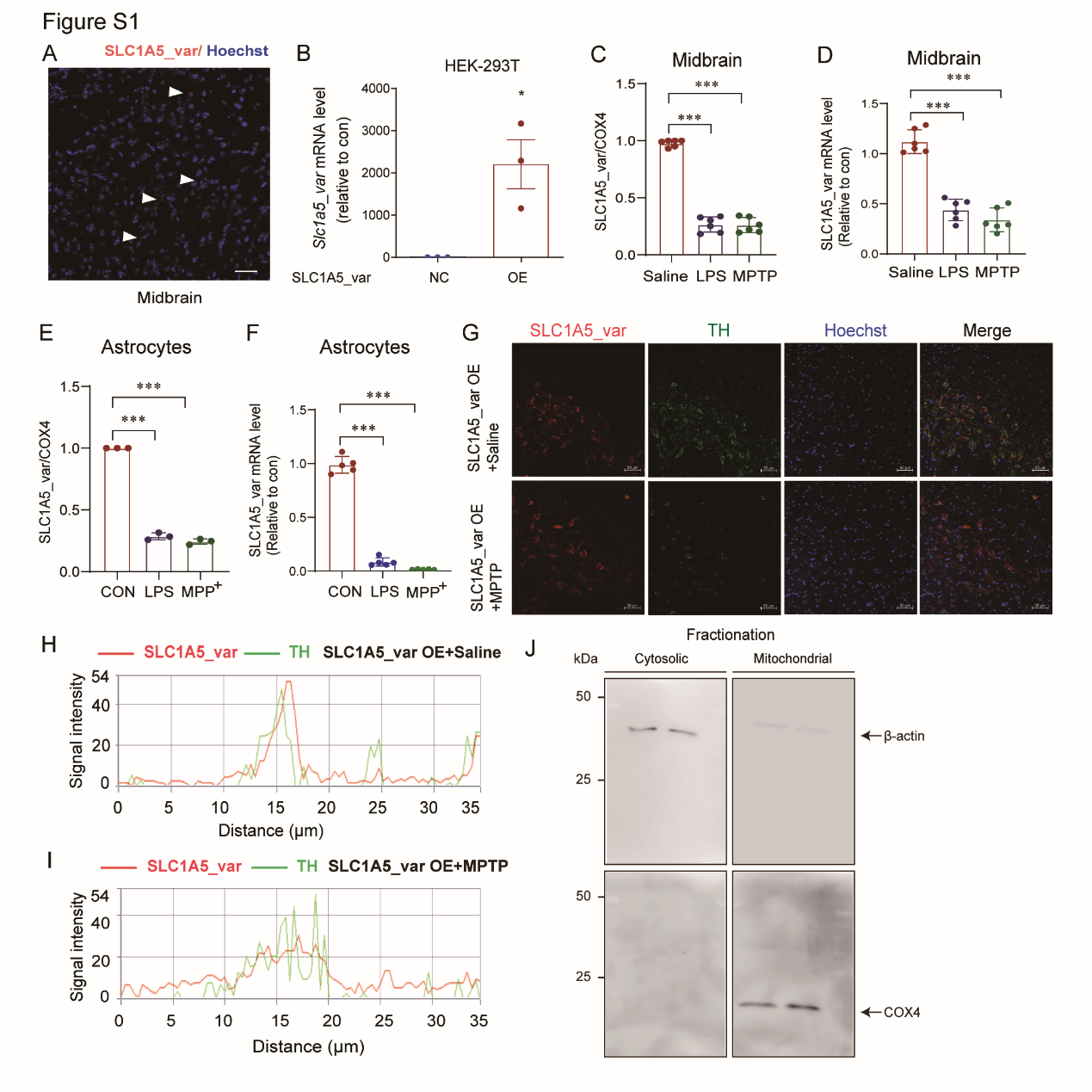


**Fig.S1** **SLC1A5_var was downregulated in MPTP and LPS-induced mice model.** (A) RNA scope of SLC1A5_var (red spot) in the midbrain. (B) HEK-293T cells were transfected with SLC1A5_var overexpression LV for 48 hours and then detected efficiency by RT-qPCR. Quantitative analysis and mRNA level of SLC1A5_var in the midbrain of MPTP and LPS mice model (C-D). Quantitative analysis and mRNA level of astrocytes stimulated by LPS and MPP^+^ (E-F). (G) Colocalization and (H-I) statistics of SLC1A5_var and TH in the midbrain. Scale bar represents 50 μm. (J) Cytosolic and mitochondrial were isolated in astrocytes. β-actin and COX-4 were detected to validate the isolation. Data were presented as mean ± S.E.M. **P* < 0.05, ***P* < 0.01, ****P* < 0.001 vs control group.


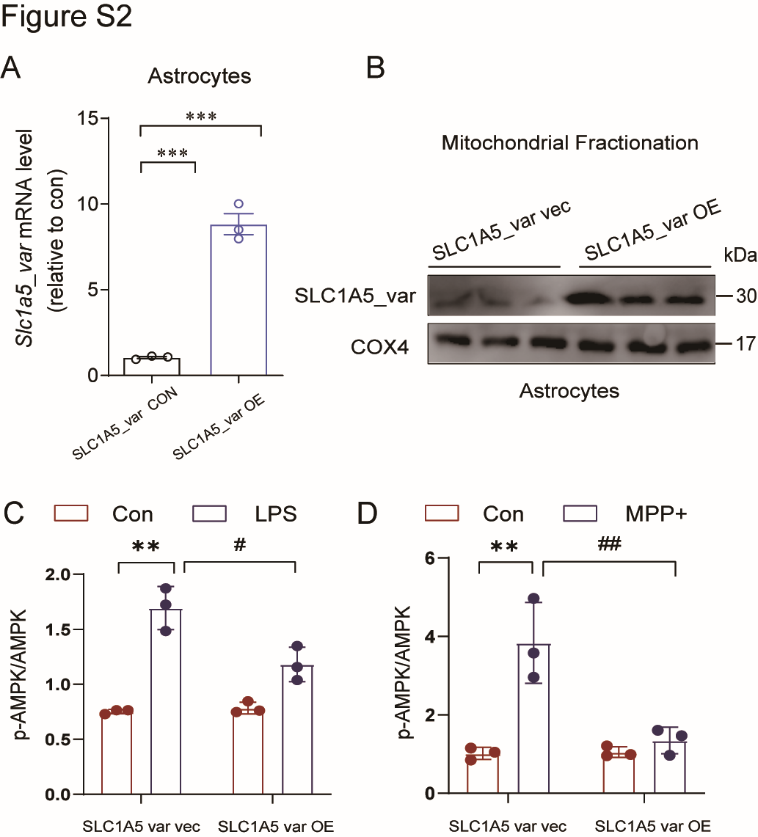


**Fig.S2 Efficiency of overexpression of SLC1A5_var in astrocytes.** Astrocytes were transfected with SLC1A5_var overexpression LV for 48 hours and then detected efficiency by RT-qPCR (A). (B) Mitochondrial fractionation was extracted to detect the expression of SLC1A5_var after astrocytes were transfected with SLC1A5_var overexpression LV. Phosphorylation of AMPK was quantified by densitometry while stimulated with LPS (C) and MPP^+^ (D). Data were presented as mean ± S.E.M. ***P* < 0.01, ****P* < 0.001 vs control group. ^#^*P* < 0.05, ^##^*P* < 0.01, ^###^*P* < 0.001 vs LPS/ MPP^+^ group Two-way ANOVA with Tukey’s post hoc test.


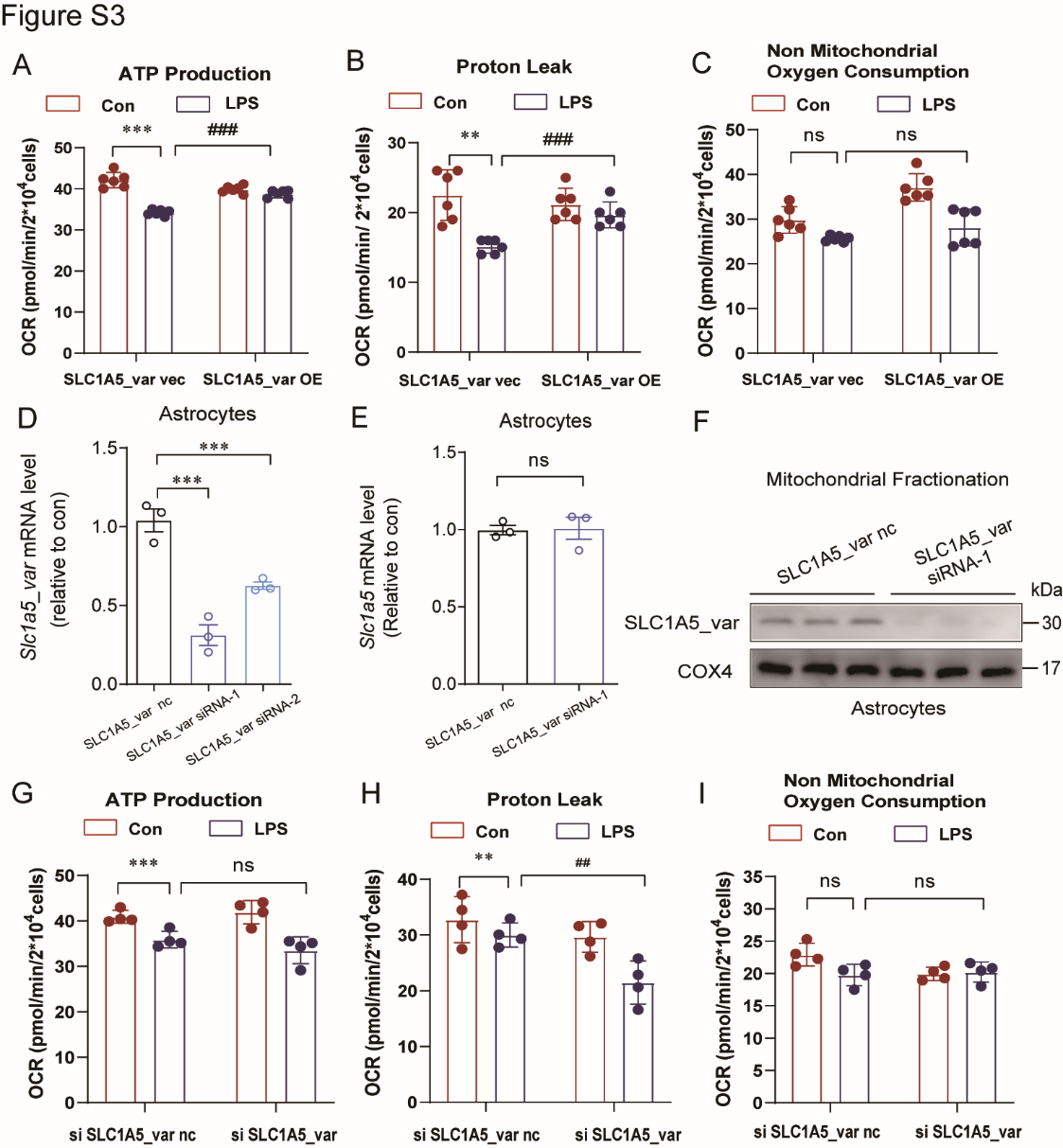


**Fig.S3 Effect of SLC1A5_var overexpression or knockdown on the oxygen consumption of astrocytes stimulated with LPS.** ATP production rate (A), proton leak (B) and non-mitochondria oxygen consumption (C) were calculated after SLC1A5_var was overexpression in astrocytes. mRNA level of SLC1A5_var (D) and SLC1A5 (E) was detected after SLC1A5_var was knockdown in astrocytes. (F) Mitochondrial fractionation was extracted to detect the expression of SLC1A5_var after SLC1A5_var was knockdown. ATP production rate (G), proton leak (H) and non-mitochondria oxygen consumption (I) were calculated after SLC1A5_var was knockdown in astrocytes. Data were presented as mean ± S.E.M. **P* < 0.05 using the Student’s t-test (B-E). Data were presented as mean ± S.E.M. ***P* < 0.01, ****P* < 0.001 vs control group. ^#^*P* < 0.05, ^##^*P* < 0.01, ^###^*P* < 0.001 vs LPS group Two-way ANOVA with Tukey’s post hoc test.


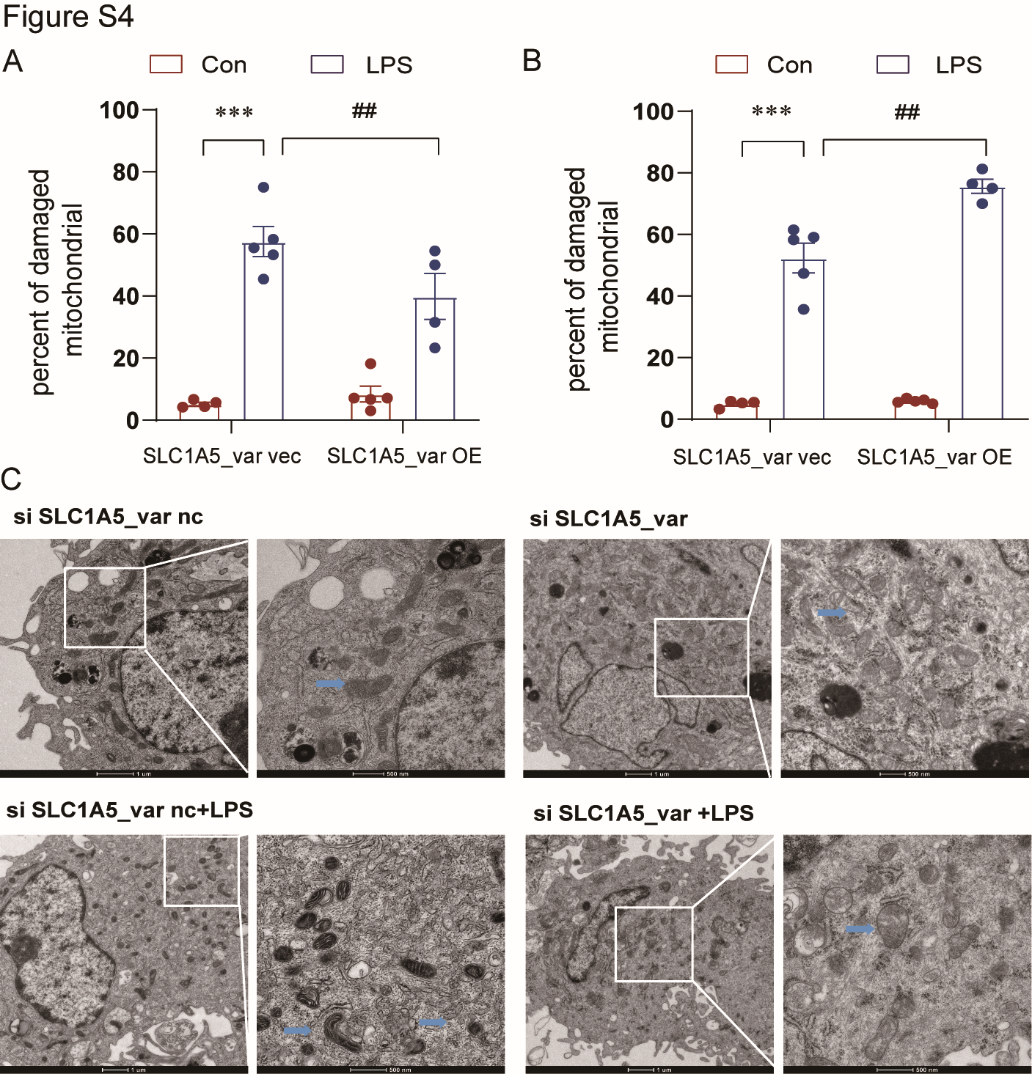


**Fig.S4 SLC1A5_var ameliorates LPS-induced dysfunction of mitochondrial in astrocytes.** The percentage of damaged mitochondria was shown while SLC1A5_var was overexpression (A) or knockdown (B). N=4-5 vision. (C) Morphology of mitochondria was shown in TEM. Blue arrows: mitochondria. Scale bar, 1 μm (left), 500 nm (enlarged vision). Data were presented as mean ± S.E.M. ***P* < 0.01, ****P* < 0.001 vs control group. ^#^*P* < 0.05, ^##^*P* < 0.01, ^###^*P* < 0.001 vs LPS group Two-way ANOVA with Tukey’s post hoc test.


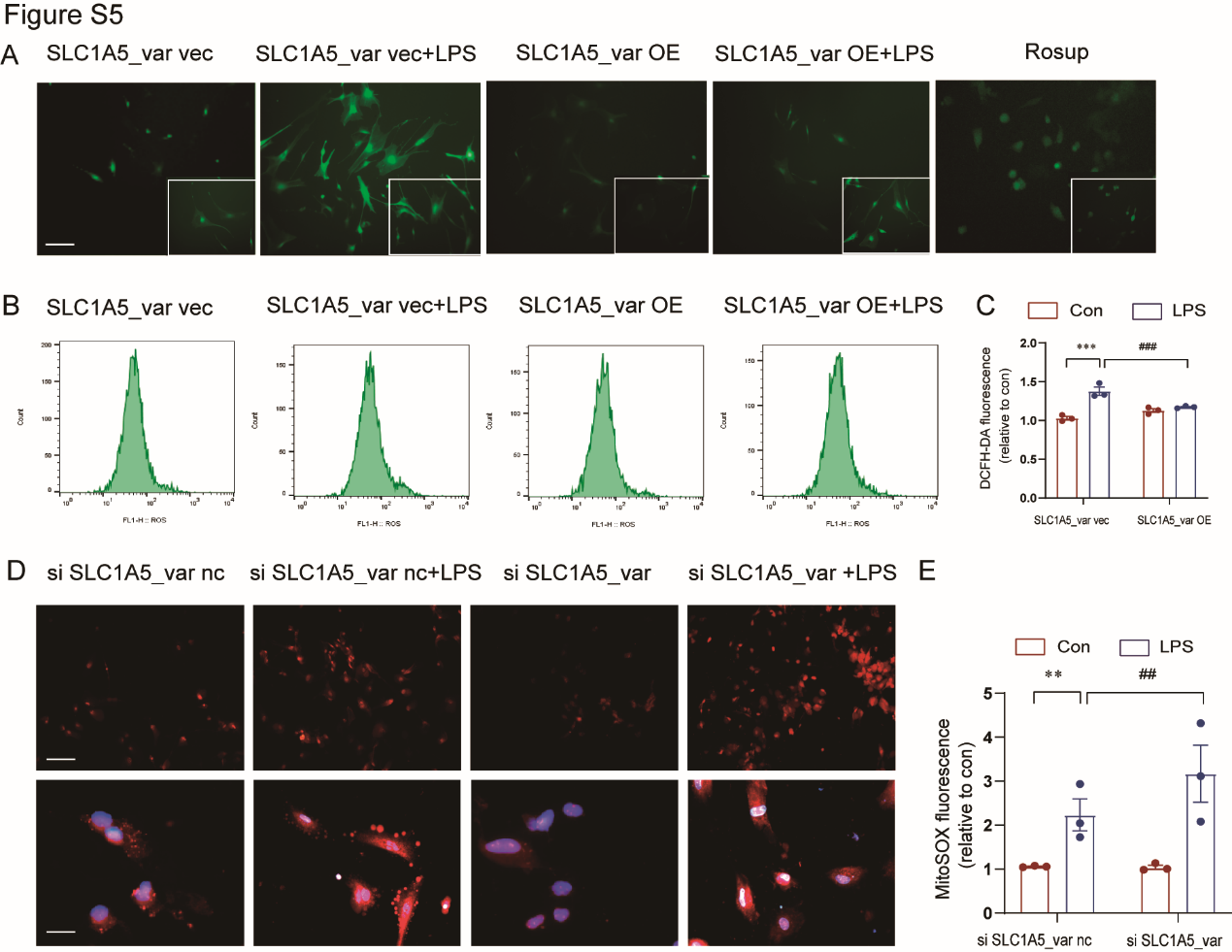


**Fig.S5 SLC1A5_var attenuates the level of the mitochondrial and intracellular reactive oxygen species (ROS) stimulated with LPS.** Intracellular reactive oxygen species (ROS) were measured by the DCFH-DA probe and monitored by fluorescence microscopy (A) and flow cytometry (B-C) while SLC1A5_var was overexpressed and then stimulated with LPS. Mitochondrial reactive oxygen species (ROS) accumulation was measured by the MitoSOX assay (D) and monitored by fluorescence microscopy (E) while SLC1A5_var was knockdown and then stimulated with LPS. Scale bar represents 50 μm (upper) and 20 μm (enlarged vision). Data were presented as mean ± S.E.M. ***P* < 0.01, ****P* < 0.001 vs control group. ^#^*P* < 0.05, ^###^*P* < 0.001 vs LPS group. Two-way ANOVA with Tukey’s post hoc test.


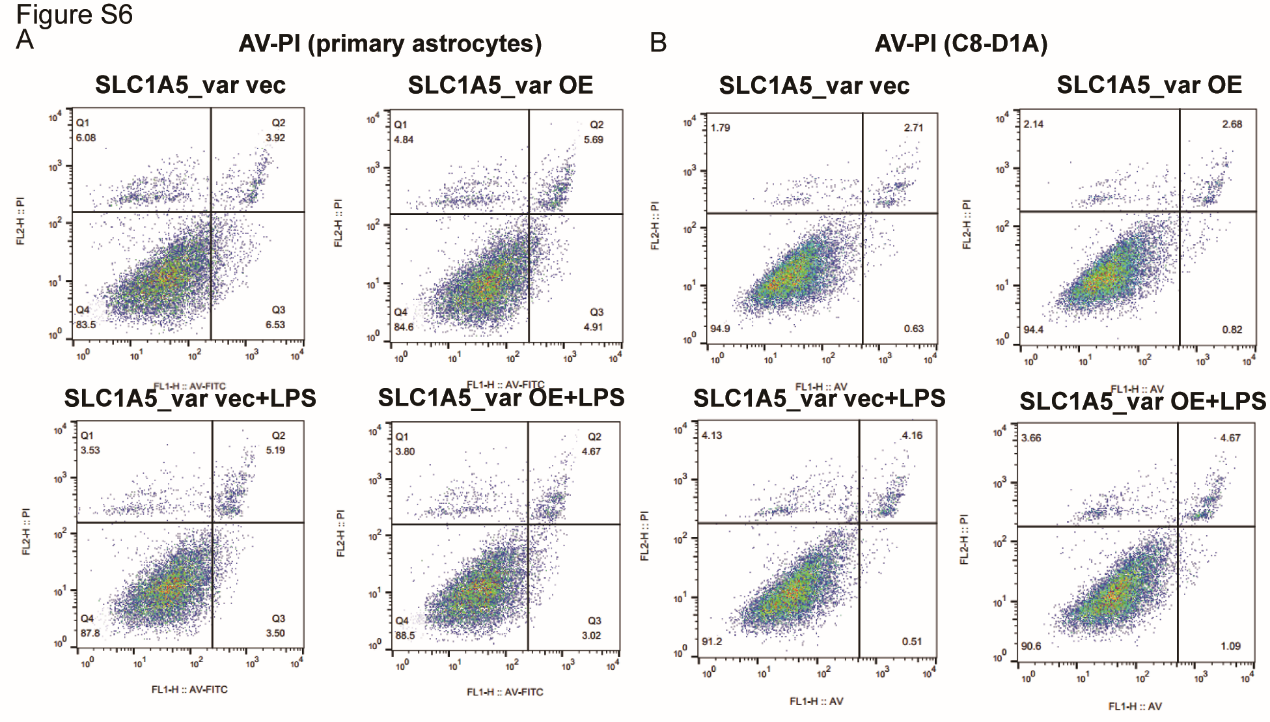


**Fig.S6 SLC1A5_var has no effect on the apoptosis of astrocytes.** Primary astrocytes (A) and astrocyte C8-D1A astrocyte cell line (B) were detected the apoptosis by flow cytometry while SLC1A5_var was overexpressed and then stimulated with LPS.


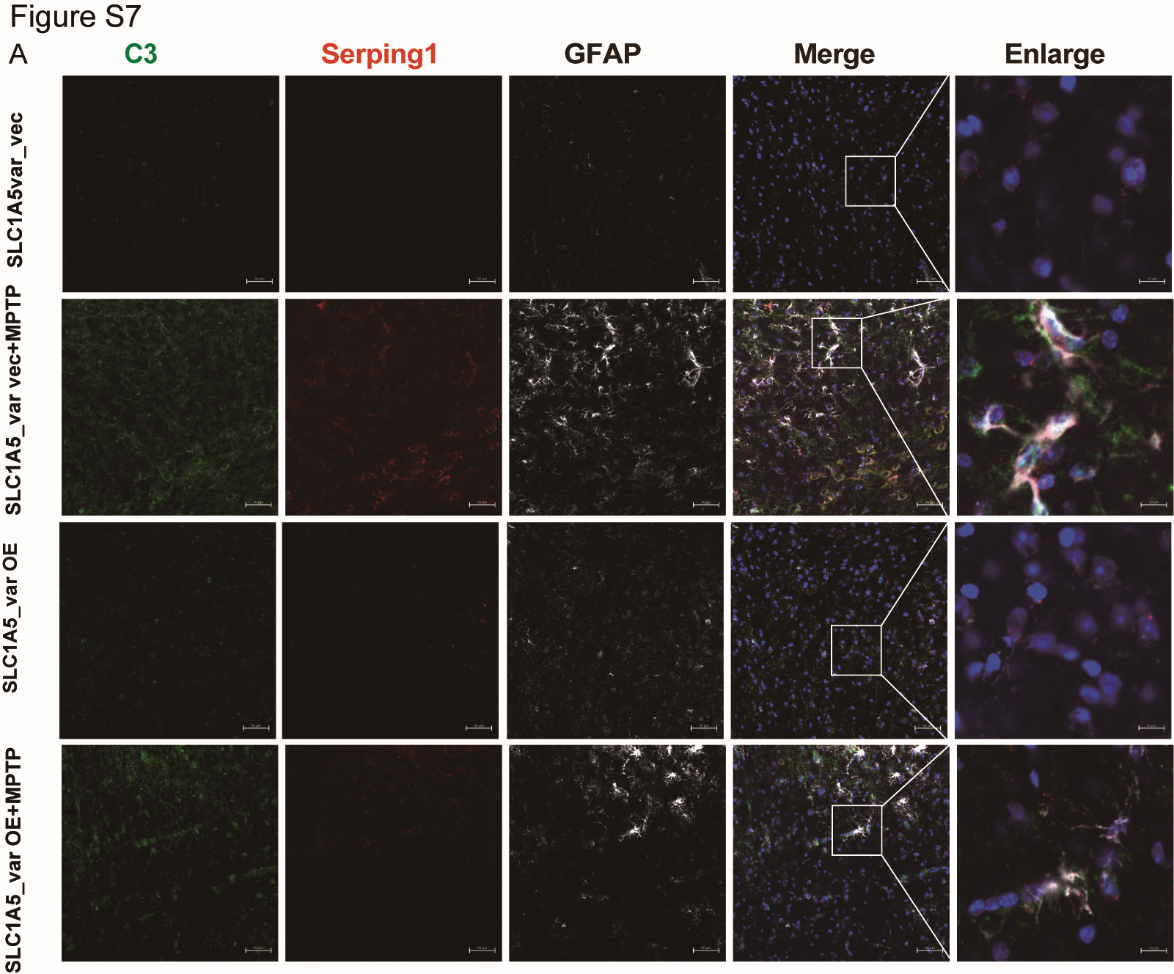


**Fig.S7 SLC1A5_var suppresses astrocyte reactivity in the MPTP mice model.** Immunofluorescent staining of C3 (green), serping1 (red), and GFAP (white) in the midbrain after LV injection with Saline and MPTP. Scale bars, 50 μm. Enlarged vision: scale bars, 10 μm.


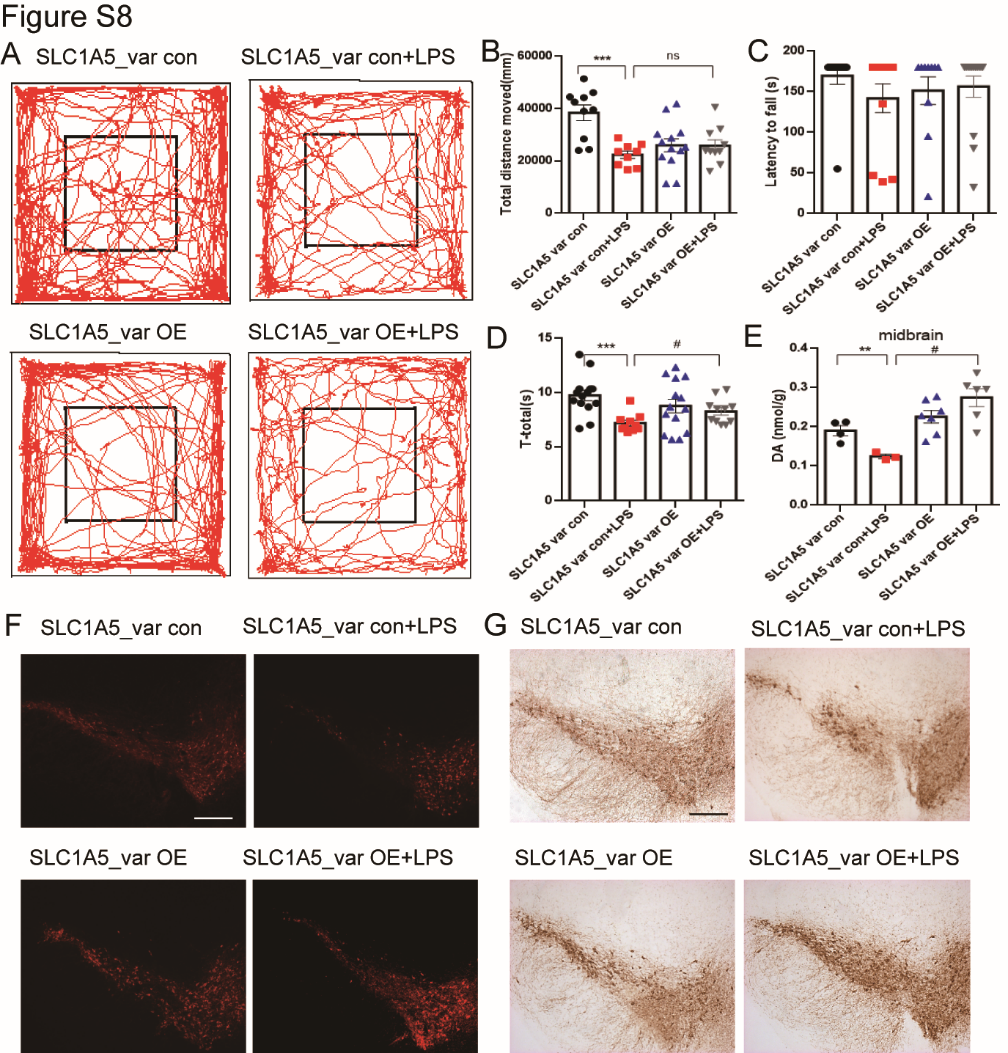


**Fig.S8 SLC1A5_var exhibited neuroprotective effects in the LPS-induced PD mice model.** (A-C) Moving routes (A-B) in the open field, (C) Latency to fall in the rotarod test and (D) time taken for mice residence in the bar (T-TLA) were detected (n=8-10). (E) The level of dopamine in the midbrain homogenate was detected by HPLC analysis (n=6-8). Immunofluorescence and immunohistochemical images of TH^+^ DA neurons in SNpc with quantification (F-G). Scale bars, 200 μm. Data are shown as the mean ± S.E.M. ***P* < 0.01 and ****P* < 0.001vs saline group. ns, *P* >0.05, ^##^*P* < 0.01, ^###^*P* < 0.001 vs LPS group. Two-way ANOVA with Tukey’s post hoc test.

**Supplementary Tables**

**Table S1 Primers used in the study.**

| Primer name | Sequence |
| --- | --- |
| *Tnf-α* | Forward: CCCTCCTTCAGACACCCT |
|  | Reverse: GGTTGCCAGCACTTCACT |
| *Il-1β* | Forward: TTGAGTCTGCCCAGTTCC |
|  | Reverse: TTTCTGCTTGAGAGGTGCT |
| *Il-6* | Forward: CAATAACCACCCCTGACC |
|  | Reverse: GCGCAGAATGAGATGAGTT |
| *Il-18* | Forward: TTGTCTCCCAGTGCATTTT |
|  | Reverse: GGTTCCTTTCCTCTTCCC |
| *Slc1a5* | Forward: CTGCCTGTGAAGGACATCTCCT |
|  | Reverse: CTCGGCATCTTGGTTCGATCCA |
| *Slc1a5_var* | Forward: TTCTTTTCTGAAGCTTCTCTGTGAG |
|  | Reverse: CCCCACAGGAATCGATAGGG |
| *Ucp2* | Forward: TAAAGGTCCGCTTCCAGGCTCA |
|  | Reverse: ACGGGCAACATTGGGAGAAGTC |
| *Agc1/Slc25a12* | Forward: GCGGAAATCCTTGCTGGAGGTT |
|  | Reverse: TGACTCTCGGTCCTGTGGTGAT |
| *Agc2/Slc25a13* | Forward: TGGCAACAGGAAAGACGTGGAG |
|  | Reverse: CCGCTCAATGTCTGCTAAGGTC |
| *β-actin* | Forward: CATTGCTGACAGGATGCAGAAGG |
|  | Reverse: TGCTGGAAGGTGGACAGTGAGG |
| *H2-T23* | Forward: GGACCGCGAATGACATAGC |
|  | Reverse: GCACCTCAGGGTGACTTCAT |
| *Ggta1* | Forward: GTGAACAGCATGAGGGGTTT |
|  | Reverse: GTTTTGTTGCCTCTGGGTGT |
| *H2-D1* | Forward: TCCGAGATTGTAAAGCGTGAAGA |
|  | Reverse: ACAGGGCAGTGCAGGGATAG |
| *Gbp2* | Forward: GGGGTCACTGTCTGACCACT |
|  | Reverse: GGGAAACCTGGGATGAGATT |
| *Ligp1* | Forward: GGGGCAATAGCTCATTGGTA |
|  | Reverse: ACCTCGAAGACATCCCCTTT |
| *Serping1* | Forward: ACAGCCCCCTCTGAATTCTT |
|  | Reverse: GGATGCTCTCCAAGTTGCTC |
| *Fbln5* | Forward: CTTCAGATGCAAGCAACAA |
|  | Reverse: CCTATGGGTCACTTGCCACT |
| *Ugt1a* | Forward: CCTATGGGTCACTTGCCACT |
|  | Reverse: AAAACCATGTTGGGCATGAT |
| *Fkbp5* | Forward: TATGCTTATGGCTCGGCTGG |
|  | Reverse: CAGCCTTCCAGGTGGACTTT |
| *Psmb8* | Forward: CAGTCCTGAAGAGGCCTACG |
|  | Reverse: CACTTTCACCCAACCGTCTT |
| *Srgn* | Forward: GCAAGGTTATCCTGCTCGGA |
|  | Reverse: TGGGAGGGCCGATGTTATTG |
| *Amigo2* | Forward: GAGGCGACCATAATGTCGTT |
|  | Reverse: GCATCCAACAGTCCGATTCT |
| *C3* | Forward: AAAAGGGGCGCAACAAGTTC |
|  | Reverse: GATGCCTTCCGGGTTCTCAA |
| *Clcf1* | Forward: CTTCAATCCTCCTCGACTGG |
|  | Reverse: TACGTCGGAGTTCAGCTGTG |
| *Ptx3* | Forward: AACAAGCTCTGTTGCCCATT |
|  | Reverse: TCCCAAATGGAACATTGGAT |
| *S100a10* | Forward: CCTCTGGCTGTGGACAAAAT |
|  | Reverse: CTGCTCACAAGAAGCAGTGG |
| *Sphk1* | Forward: GATGCATGAGGTGGTGAATG |
|  | Reverse: TGCTCGTACCCAGCATAGTG |
| *Cd109* | Forward: CACAGTCGGGAGCCCTAAAG |
|  | Reverse: GCAGCGATTTCGATGTCCAC |
| *Ptgs2* | Forward: GCTGTACAAGCAGTGGCAAA |
|  | Reverse: CCCCAAAGATAGCATCTGGA |
| *Emp1* | Forward: GAGACACTGGCCAGAAAAGC |
|  | Reverse: GCAGCGATTTCGATGTCCAC |
| *Slc10a6* | Forward: GCTTCGGTGGTATGATGCTT |
|  | Reverse: CCACAGGCTTTTCTGGTGAT |
| *Tm4sf1* | Forward: GCCCAAGCATATTGTGGAGT |
|  | Reverse: AGGGTAGGATGTGGCACAAG |
| *B3gnt5* | Forward: CGTGGGGCAATGAGAACTAT |
|  | Reverse: CCCAGCTGAACTGAAGAAGG |
| *Cd14* | Forward: GGACTGATCTCAGCCCTCTG |
|  | Reverse: GCTTCAGCCCAGTGAAAGAC |
| *Slc1a5_var* siRNA | Forward: GCUGCCCUCCCACUAUGUATT |
|  | Reverse: UACAUAGUGGGAGGGCAGCTT |

**Table S2 Antibodies used in the study.**

| Antibody | Source | Catalog |
| --- | --- | --- |
| p-AMPKα (Thr172) (D4D6D) | CST | #50081 |
| AMPKα (D5A2) | CST | #5831 |
| p-NF-kB p65(Ser536) | CST | #3033 |
| NF-kB p65 (D14E12) | CST | #8242 |
| p-IKKβ (Ser176/180) | CST | #2694 |
| IKKβ (L570) | CST | #2678 |
| p-p38 MAPK (Thr180/Tyr182) (D3F9) | CST | #4511 |
| p38 MAPK (D13E1) | CST | #8690 |
| p-SAPK/JNK (Thr183/ Tyr185) | CST | #9251 |
| SAPK/JNK | CST | #9252 |
| p-AKT (Ser473) (D9E) | CST | #4060 |
| AKT | CST | #9272 |
| β-actin | CST | #3700 |
| TLR4 | Santa Cruz | sc-293072 |
| ASCT2 (D7C12) | CST | #8057 |
| goat anti-rabbit IgG | CST | #074-1516 |
| goat anti-mouse IgG | CST | #074-1806 |
| Tom20 | Abcam | ab186735 |
| COX4 | Abcam | ab110261 |
| Complement C3 | Thermo | PA5-21349 |
| Complement C3 (11H9) | Novus | NB200-540 |
| serping1 | Proteintech | 12259-1-AP |
| ASCT2 | Novus | NBP1-89327 |
| Tyrosine Hydrolase | Millipore | #SAB2103892 |
| Tyrosine Hydrolase (F-11) | Santa Cruz | sc-25269 |
| GFAP | Millipore | #MAB360 |
| GFAP | Abcam | ab7206 |
| Goat anti-Mouse IgG (Alexa Fluor® 555 conjugate) | Invitrogen | #A21422 |
| Goat anti-Rabbit IgG (Alexa Fluor® 488 conjugate) | Invitrogen | #A11008 |
| Goat anti- Rabbit IgG (Alexa Fluor® 555 conjugate) | Invitrogen | #A31572 |
| Goat anti- Mouse IgG (Alexa Fluor® 488 conjugate) | Invitrogen | #A21202 |
| Goat anti-Rat IgG (Alexa Fluor® 488 conjugate) | Invitrogen | #A11006 |
