# Supplementary material for: Mitochondrial glutamine transporter SLC1A5_var, a potential target to suppress astrocyte reactivity in Parkinson’s Disease
Source: Cell Death Dis. 2022 Nov 9;13(11):946. doi: 10.1038/s41419-022-05399-z (PMC9646772; doi:10.1038/s41419-022-05399-z)

SLC1A5_var WB gel

Figure 1H

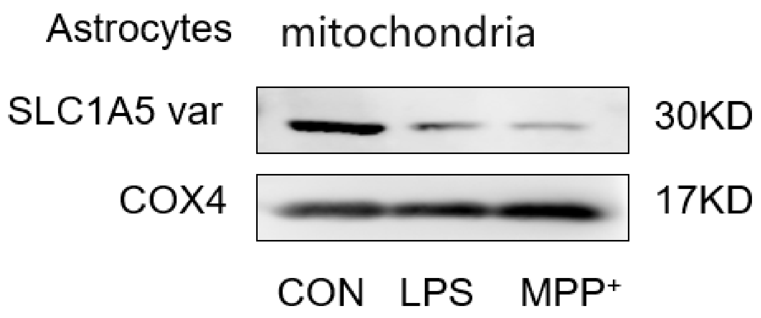

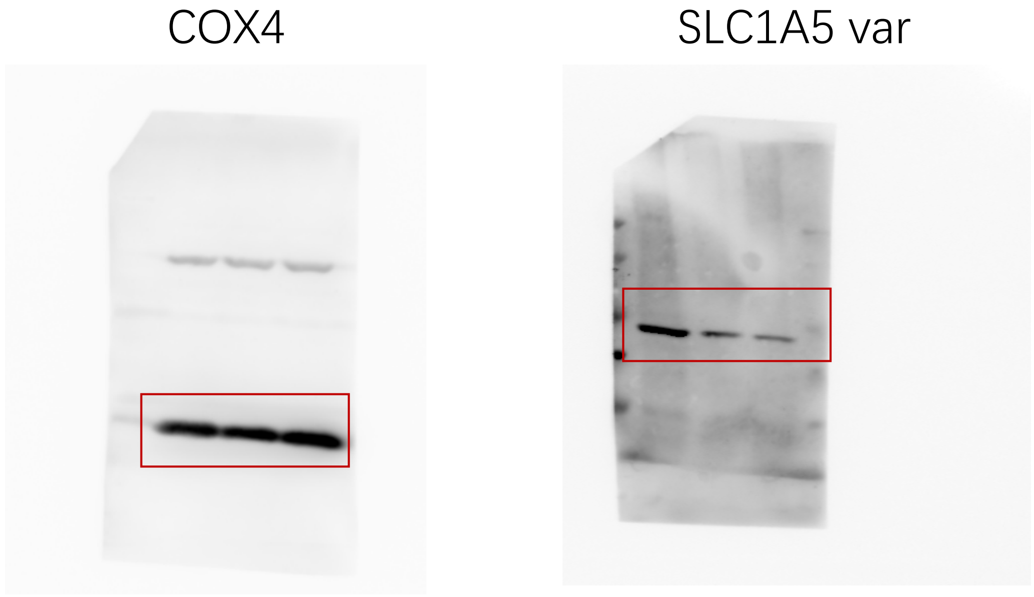

Figure 1I

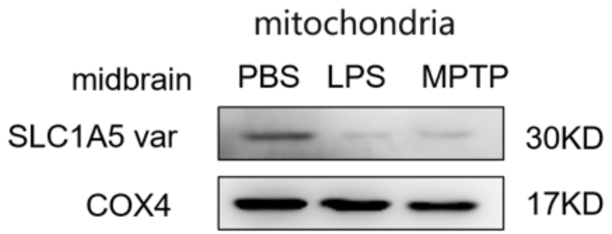

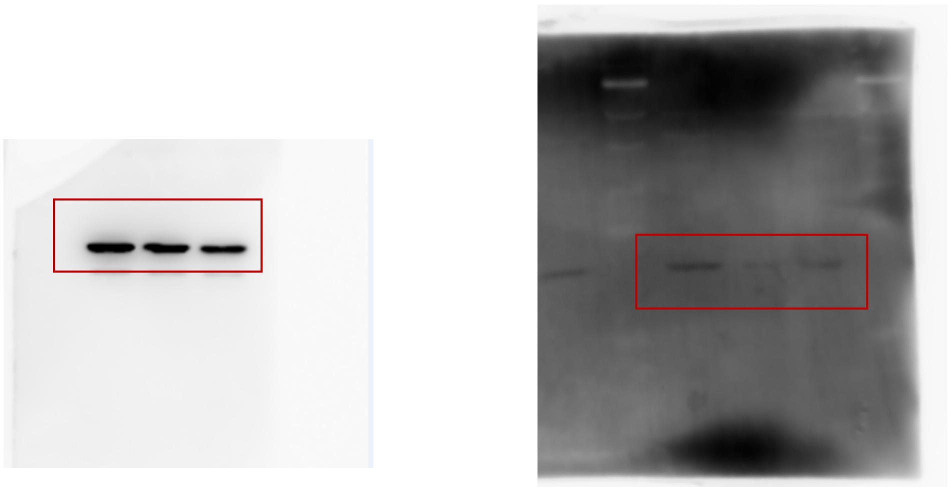

Figure 1N

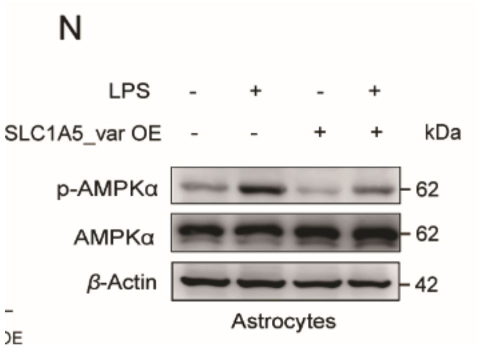

P-AMPK

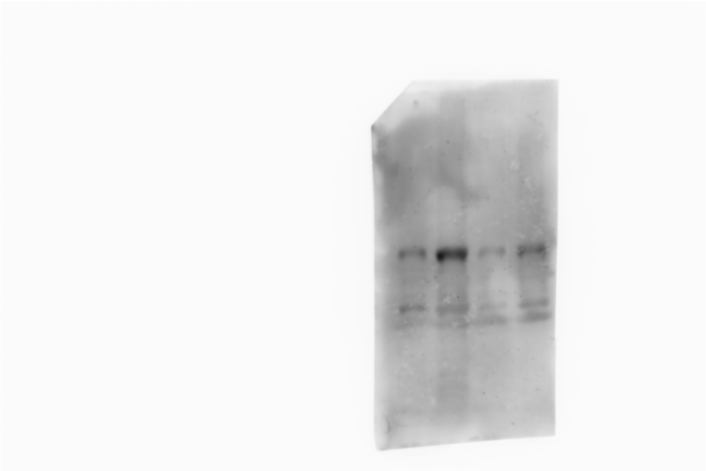

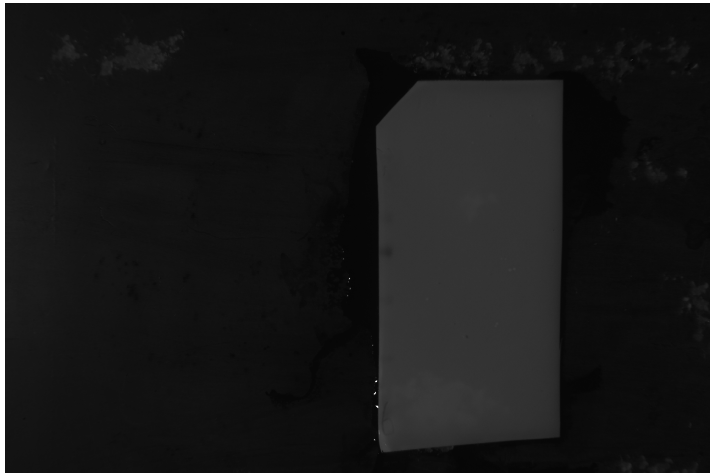

P-AMPK

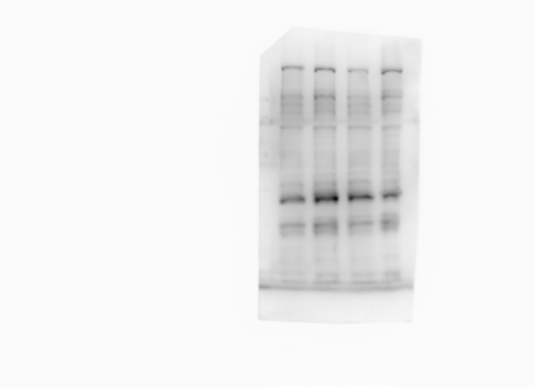

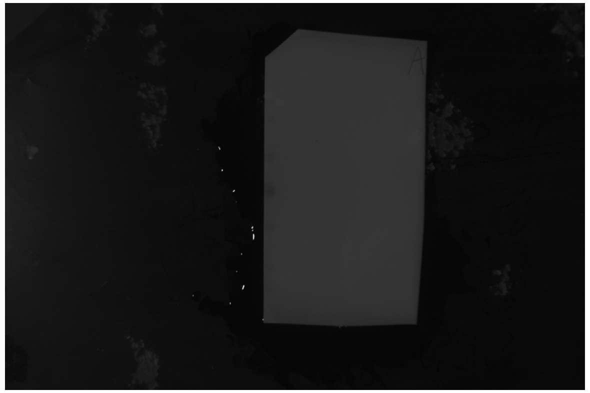

P-AMPK

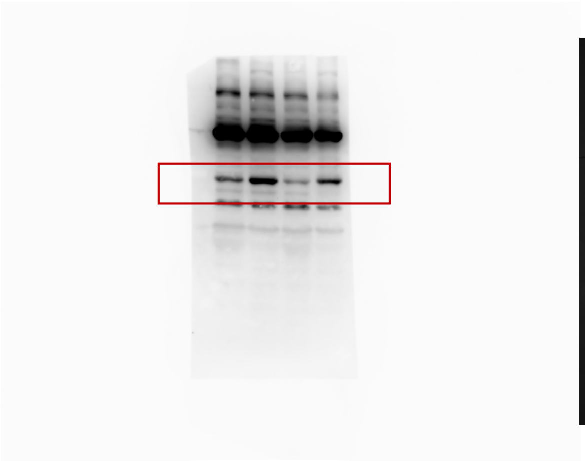

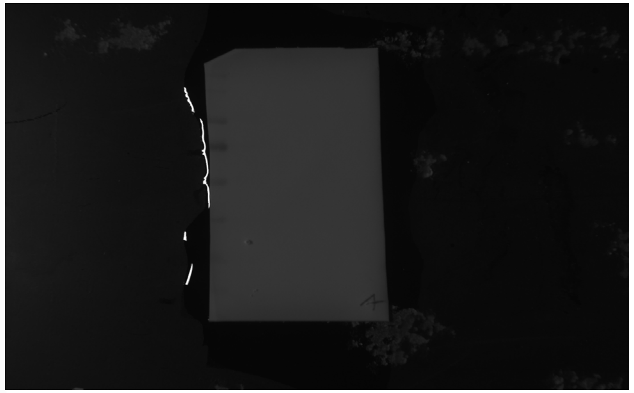

AMPK

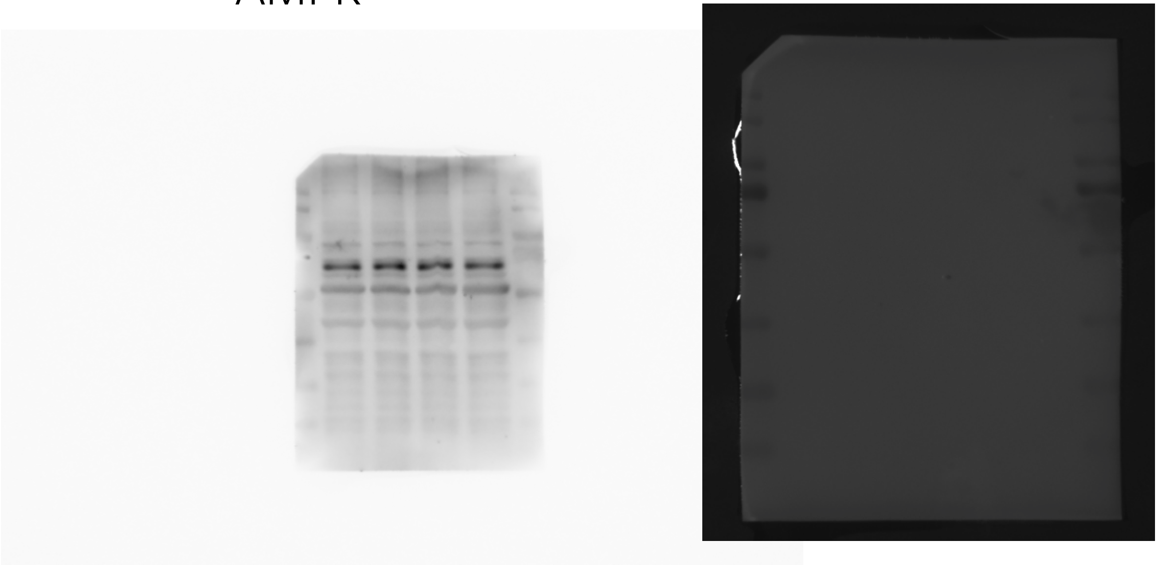

AMPK

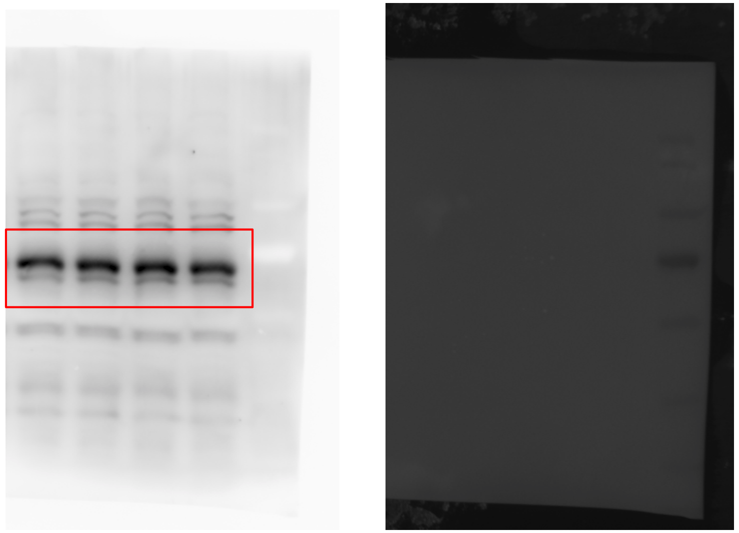

AMPK

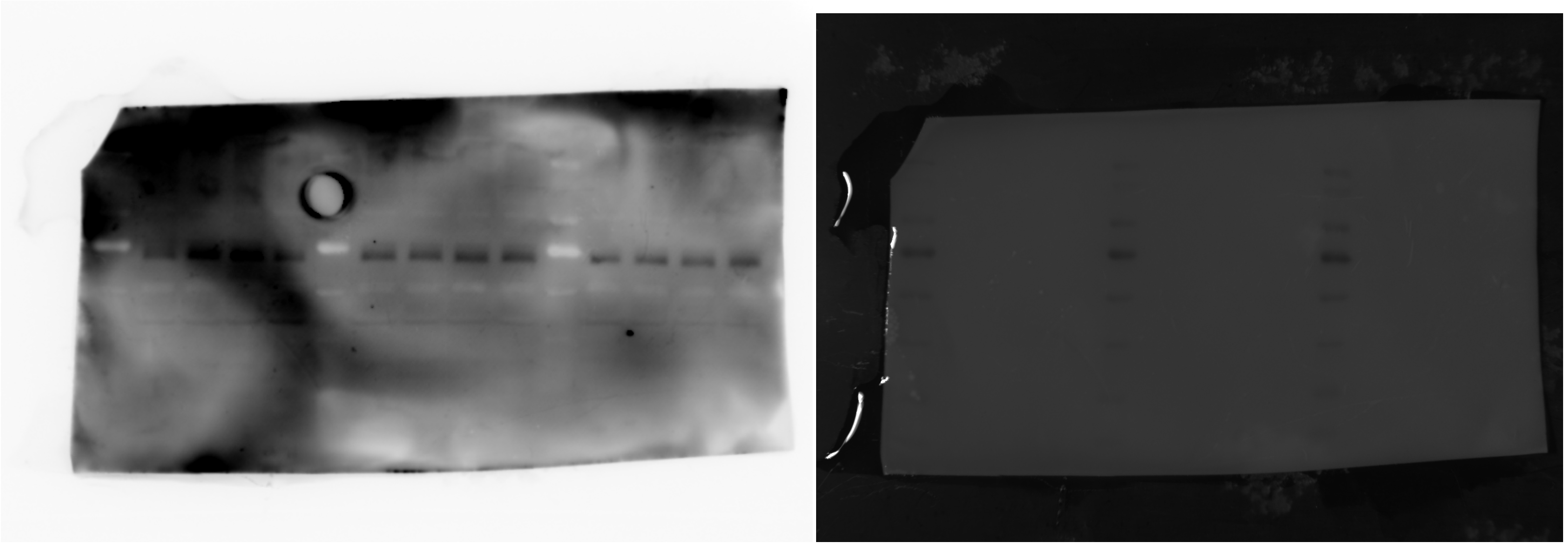

actin

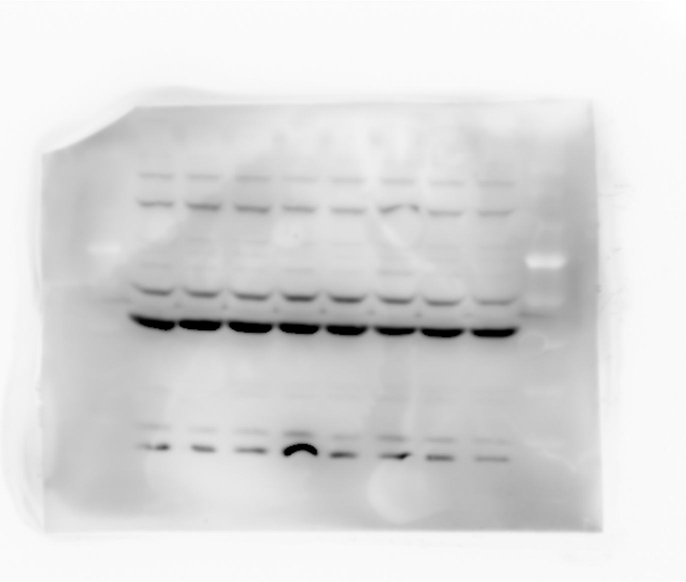

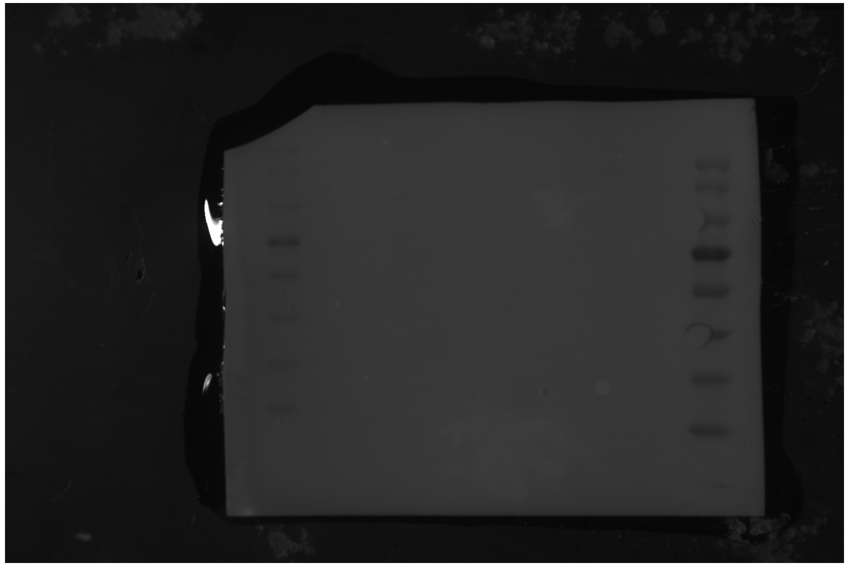

actin

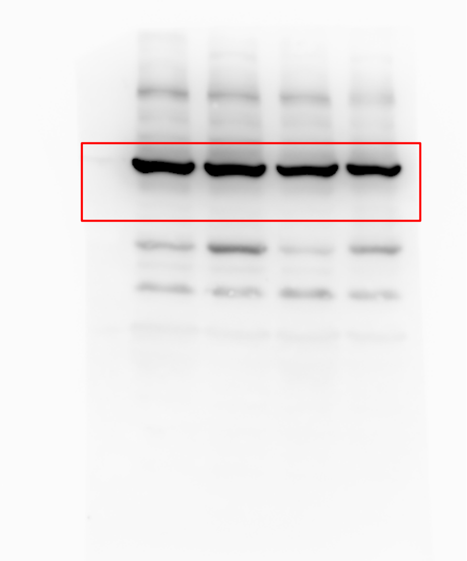

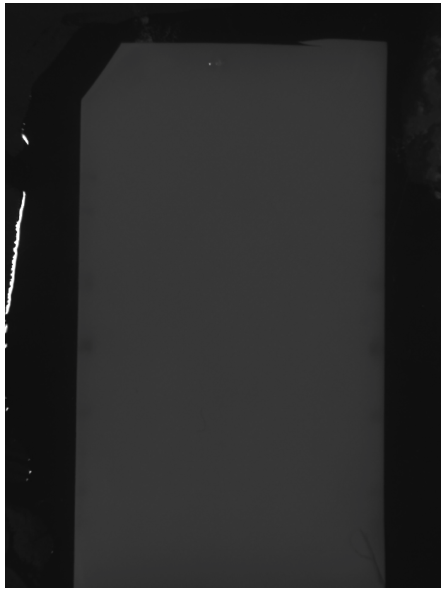

Figure 1P

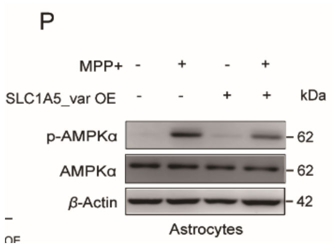

p-AMPK

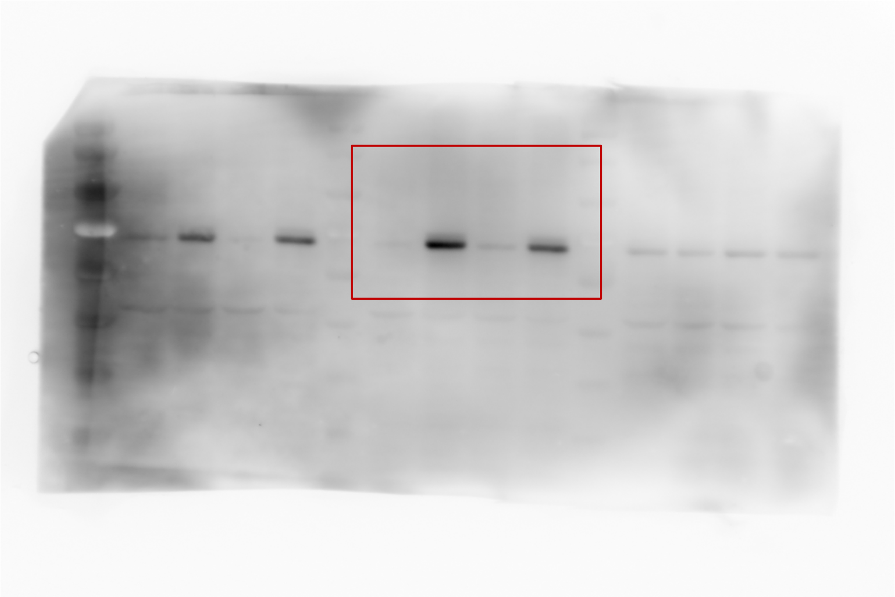

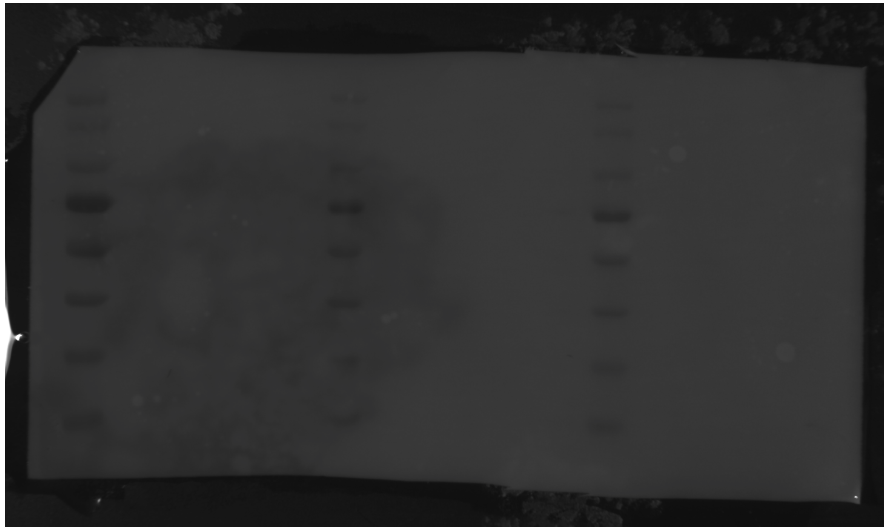

Actin

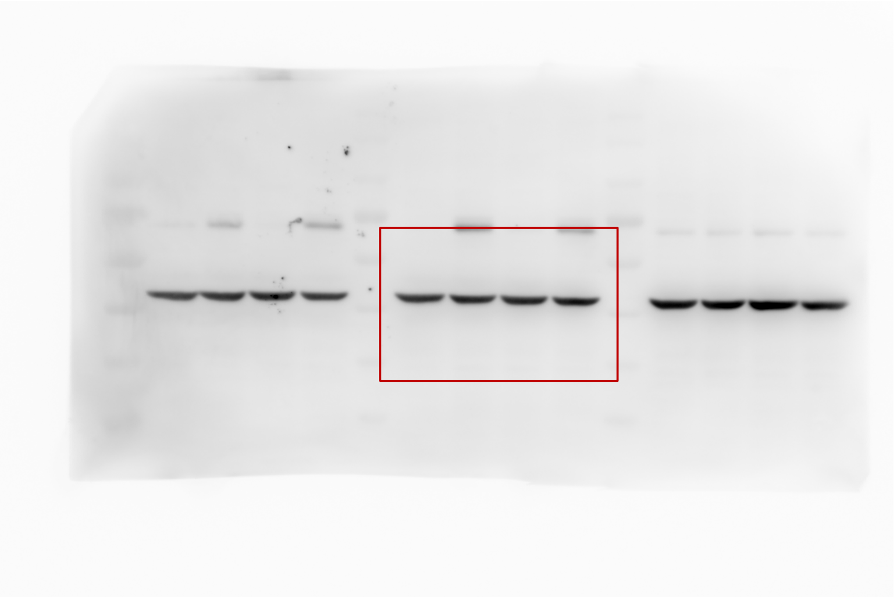

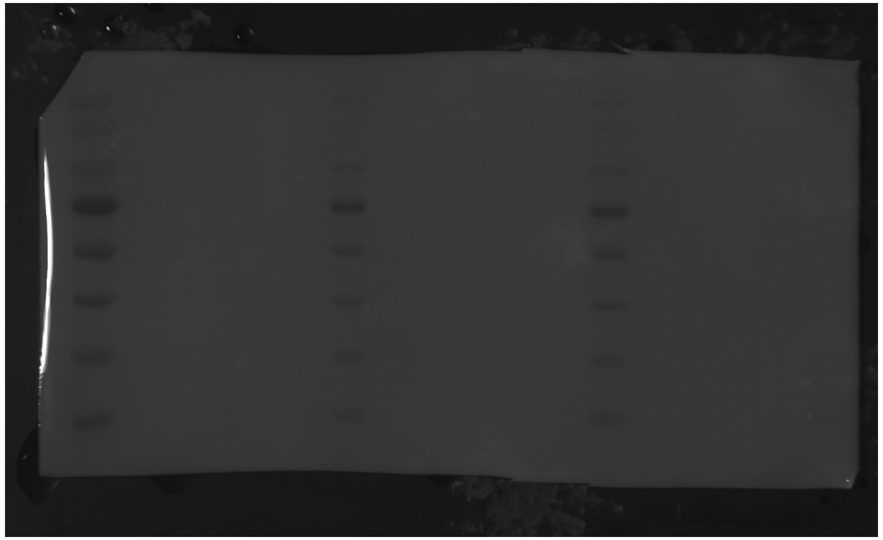

Figure 1P

p-AMPK

actin

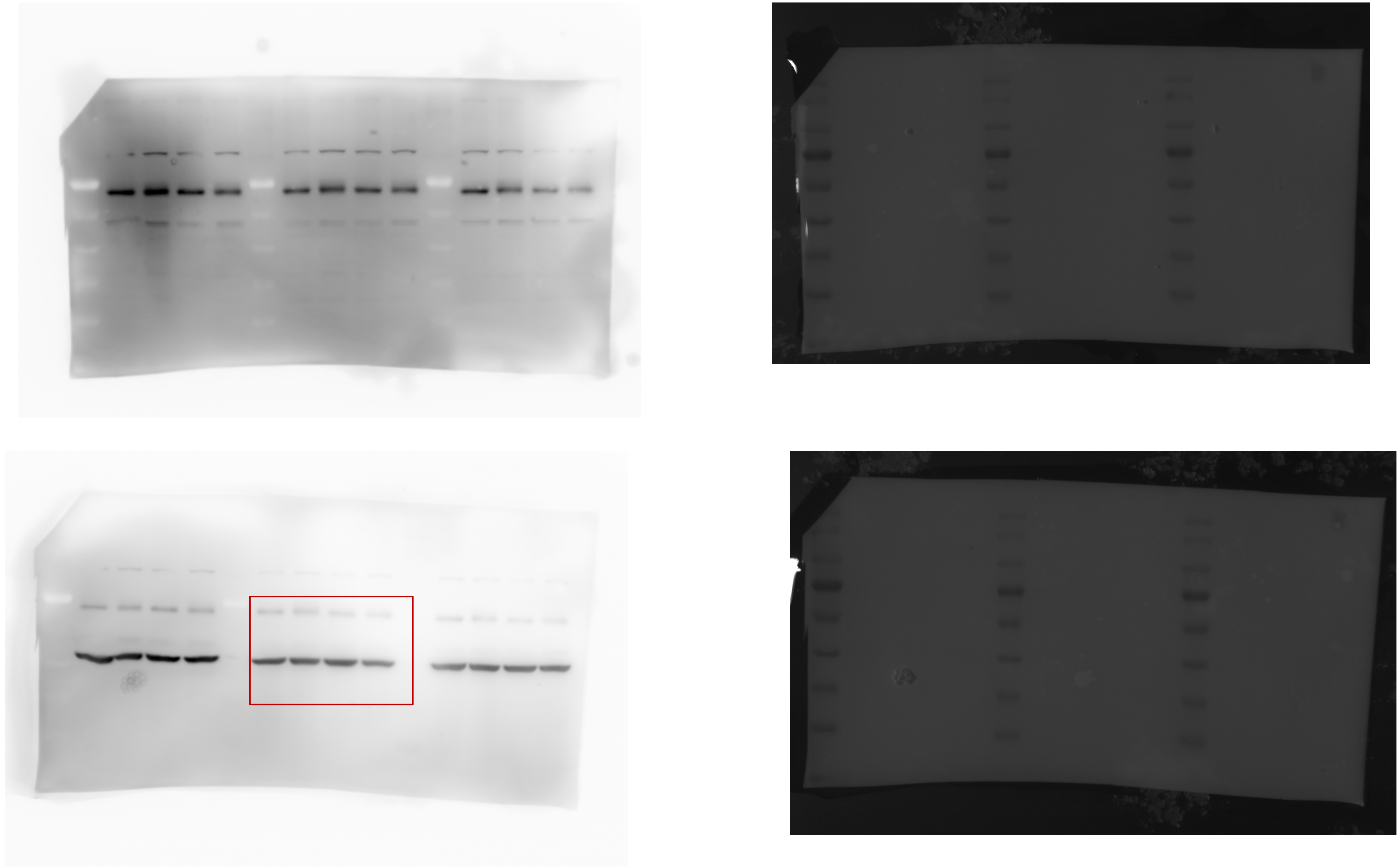

AMPK

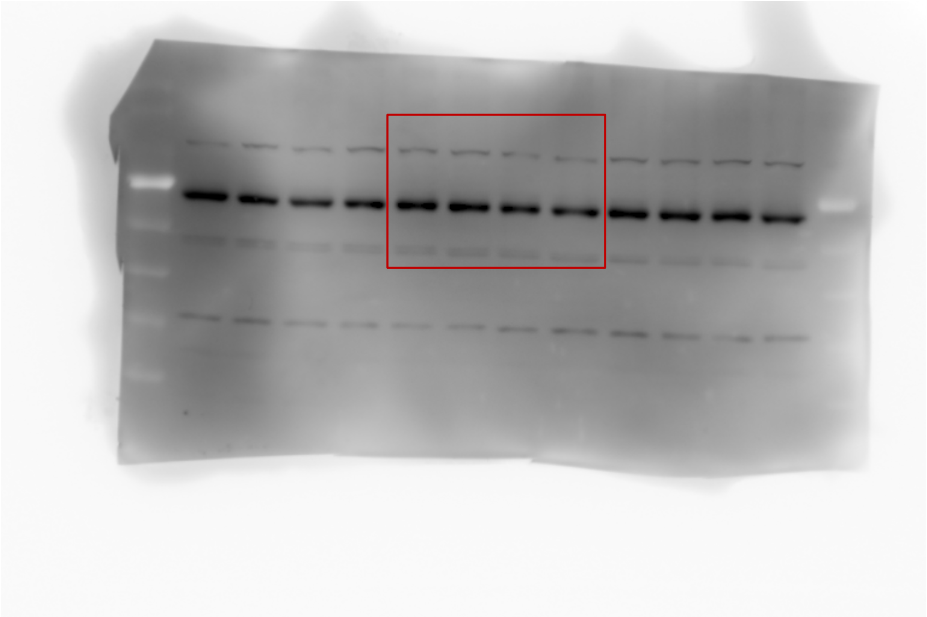

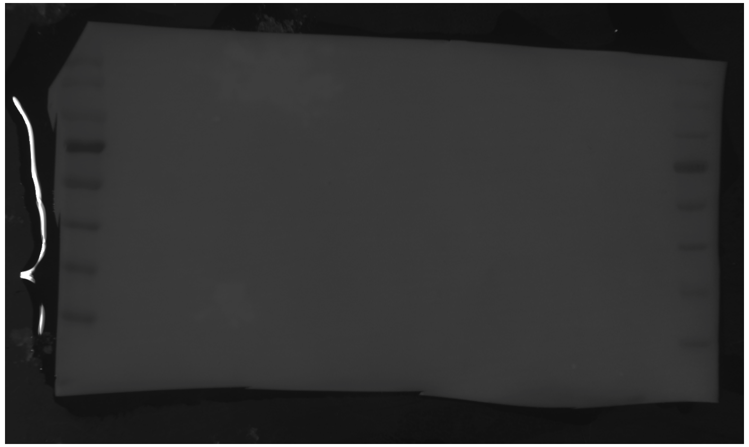

actin

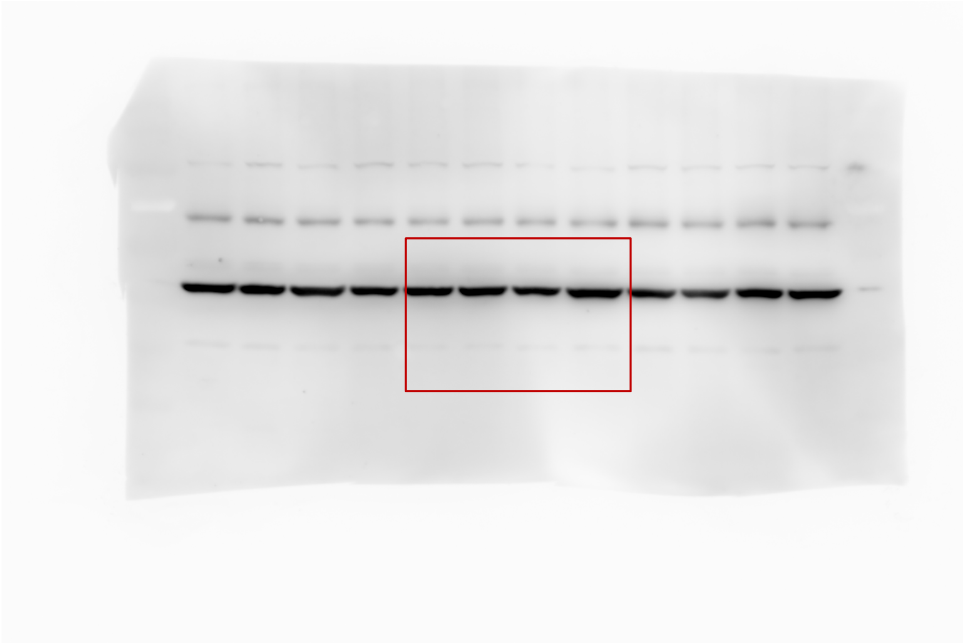

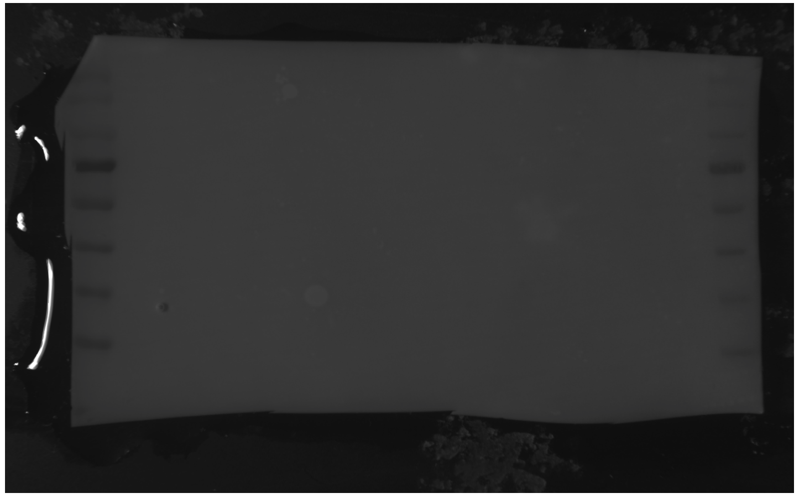

Figure S2B

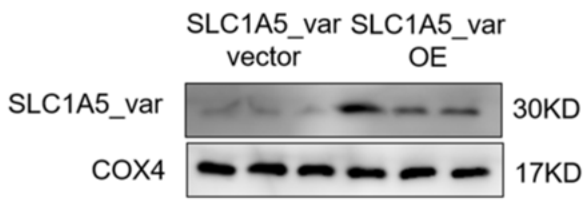
SLC1A5_var vector
SLC1A5_var OE
SLC1A5_var
30KD
COX4
17KD

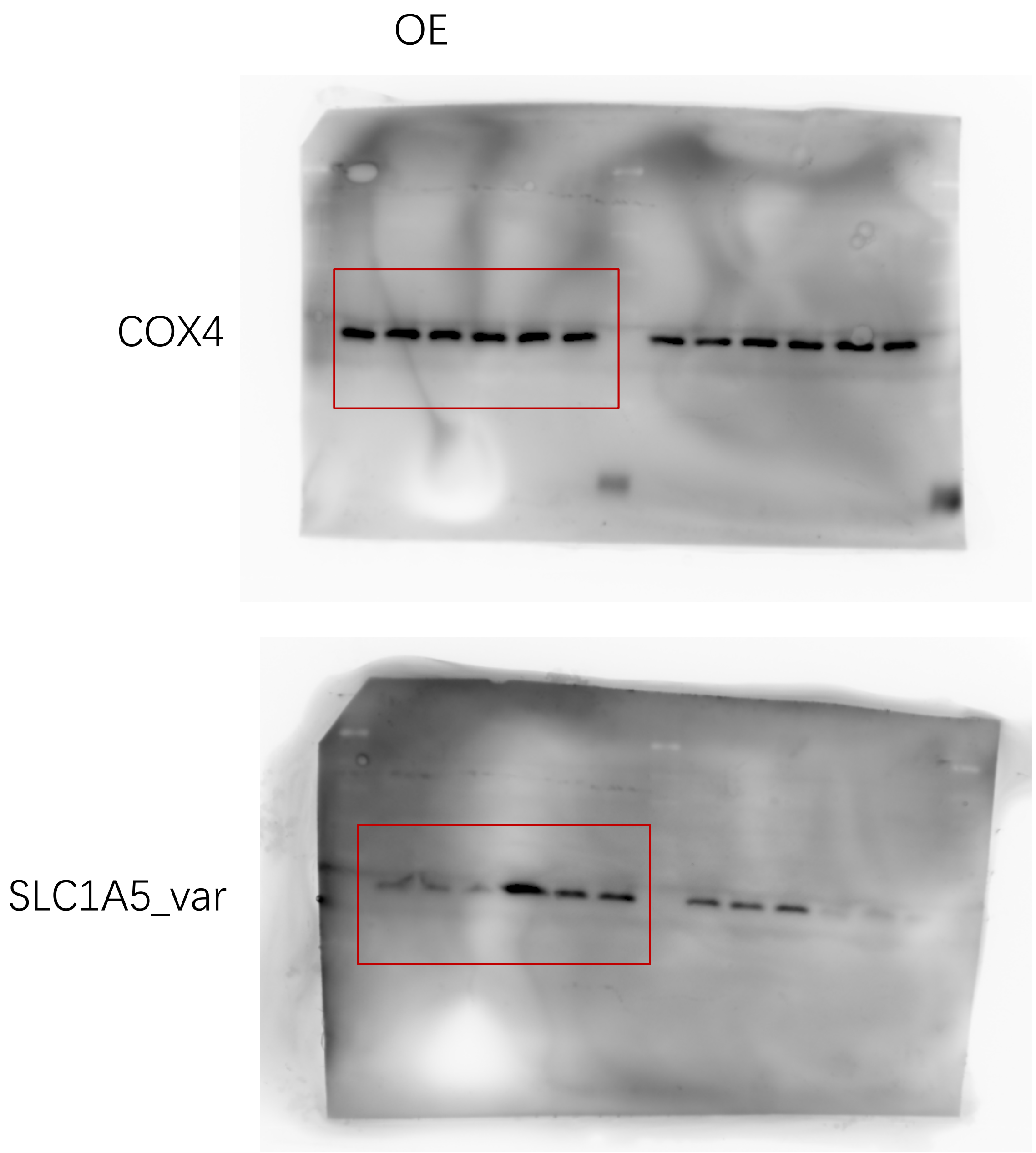
OE
COX4
SLC1A5_var

Figure
S3F

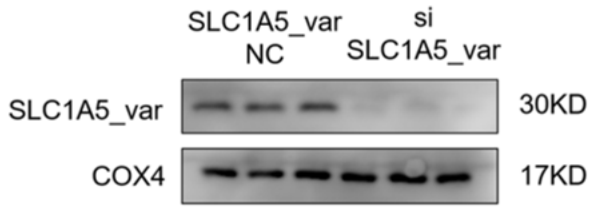

SLC1A5_var
NC
si
SLC1A5_var
SLC1A5_var
30KD
COX4
17KD

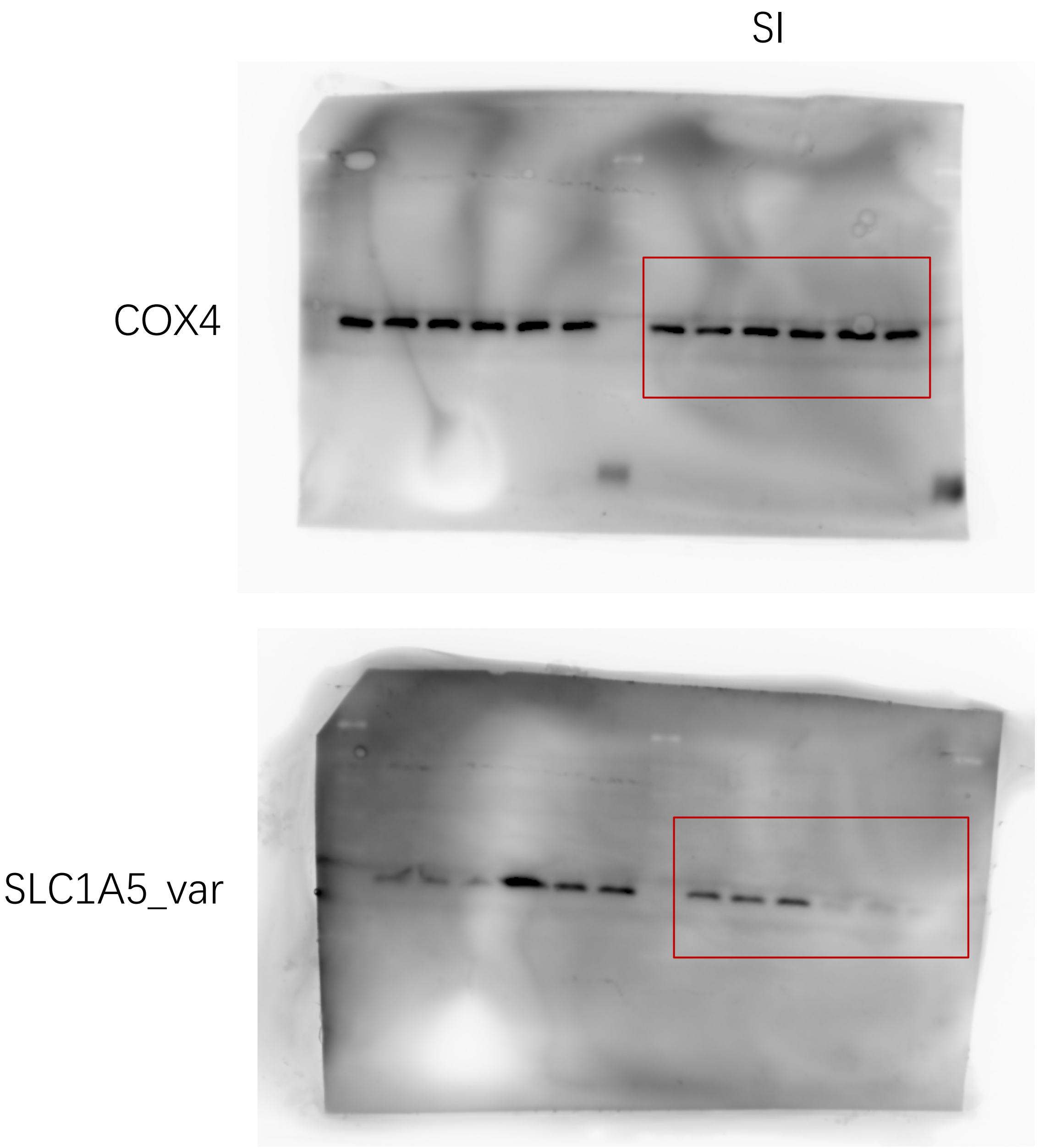

SI
COX4
SLC1A5_var

Figure 4

IKK P-IKK

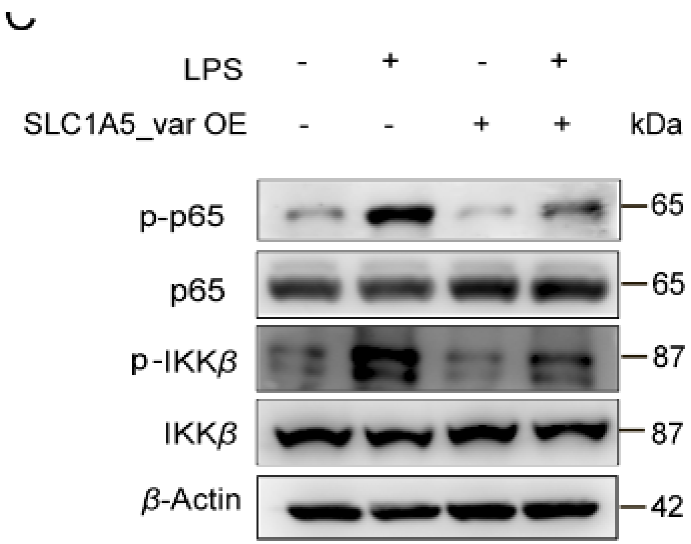

p65

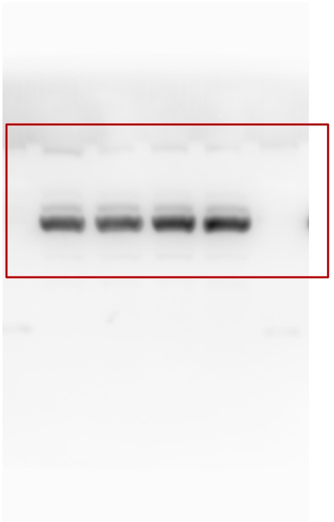

p65

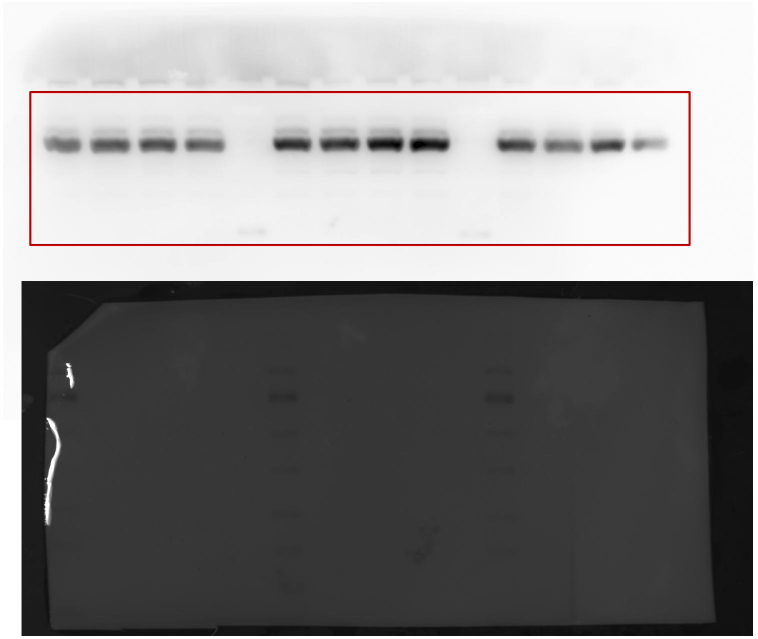

p-p65

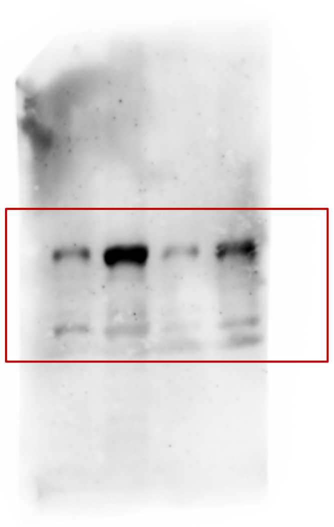

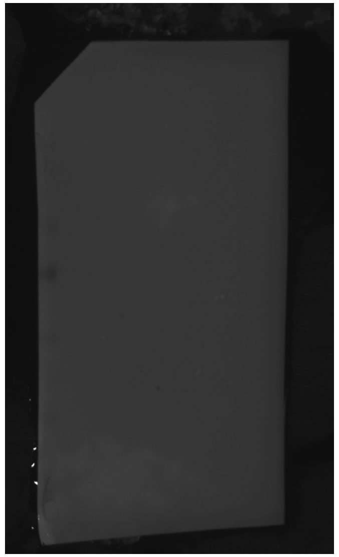

p-p65

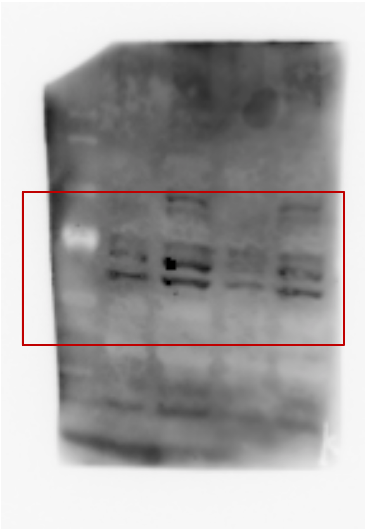

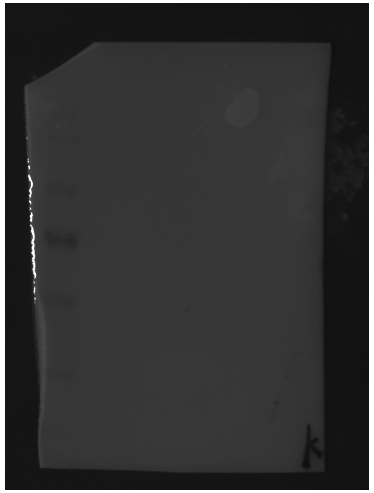

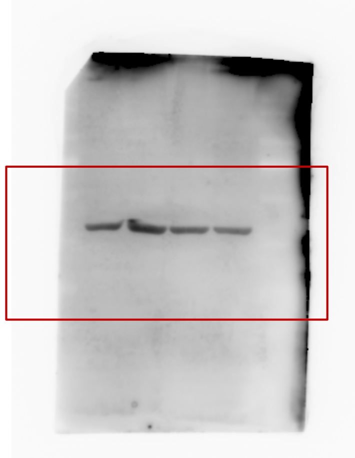

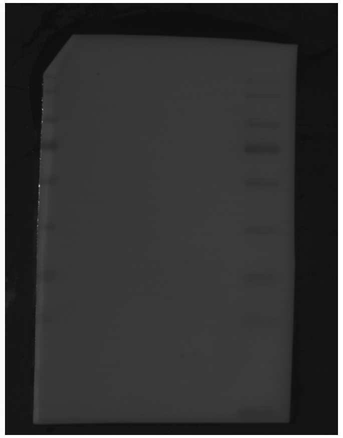

F

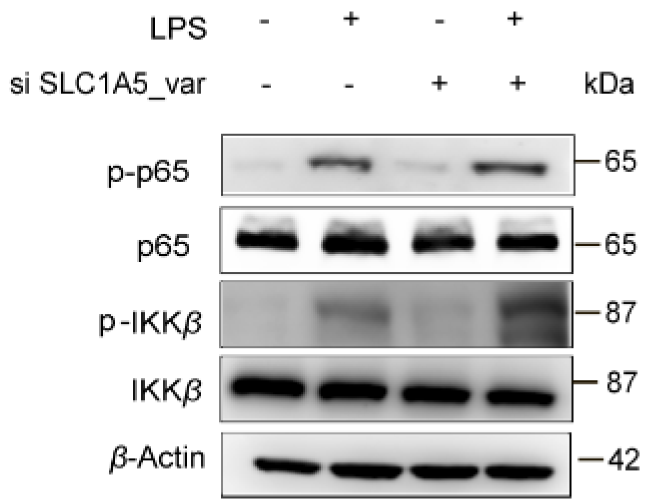

p65

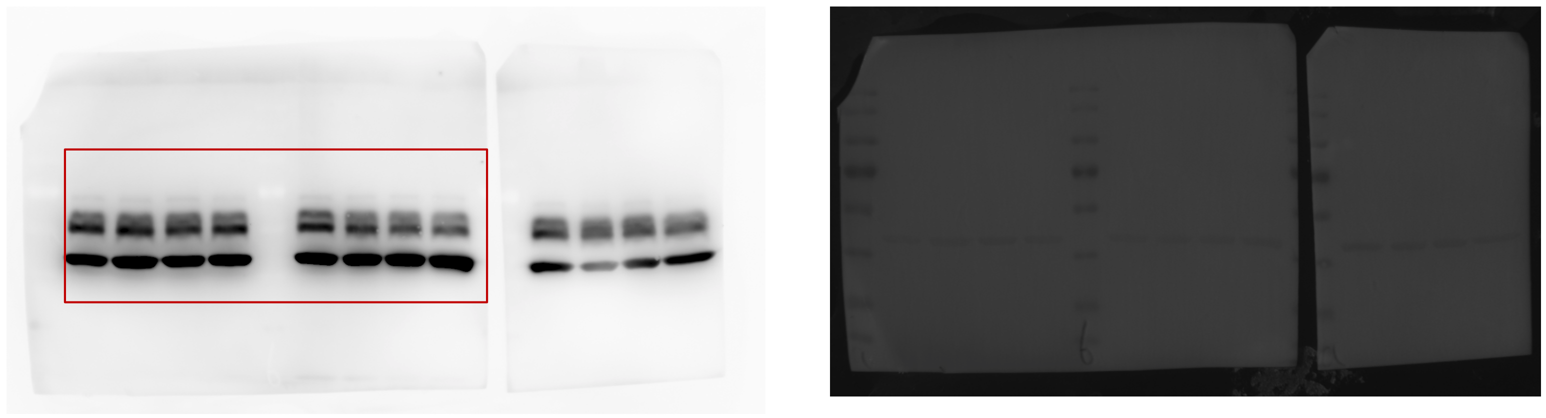

p-p65

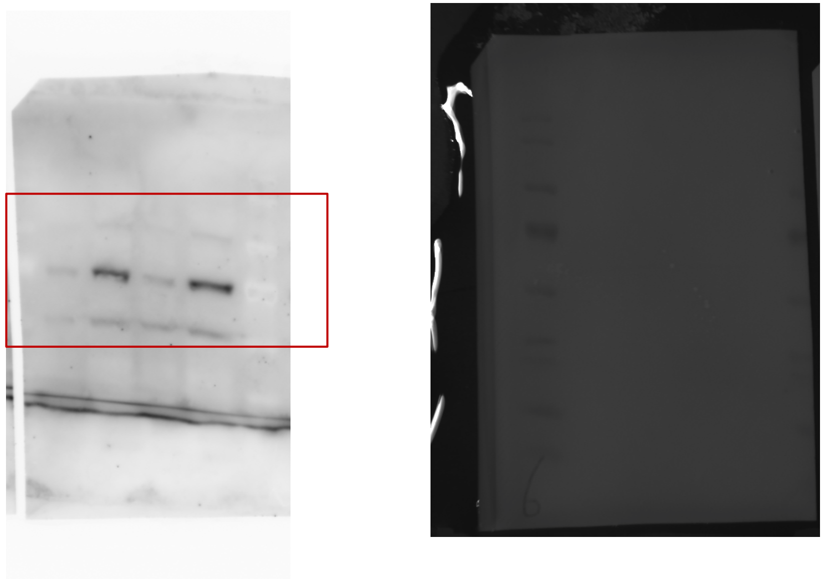

p-p65

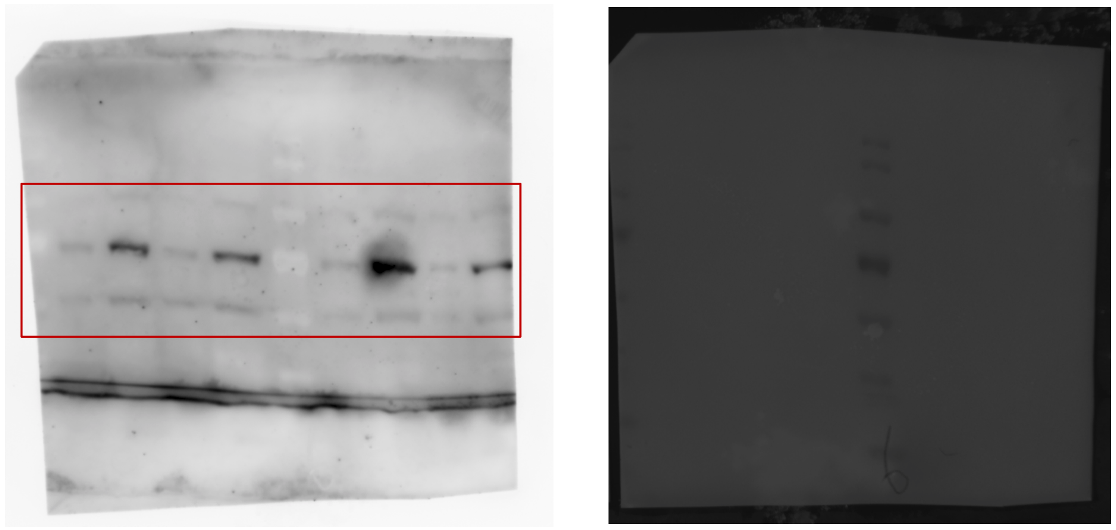

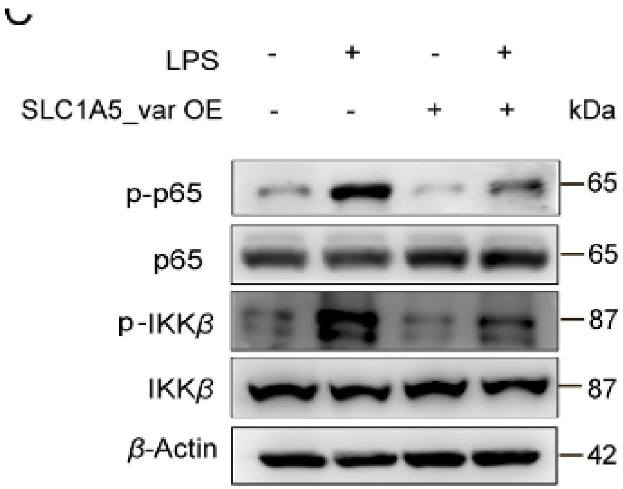
c
LPS - + - +
SLC1A5_var OE - - + + kDa
p-p65 65
p65 65
p-IKKβ 87
IKKβ 87
β-Actin 42

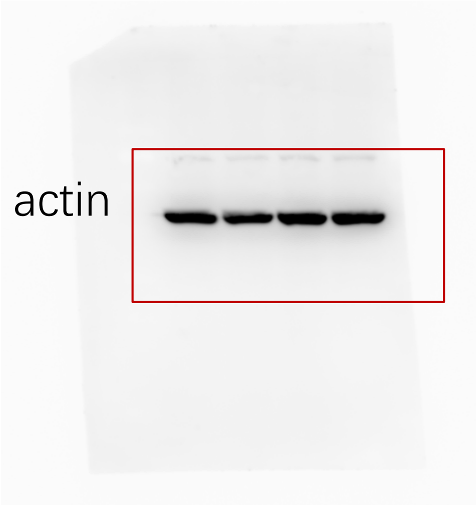
actin

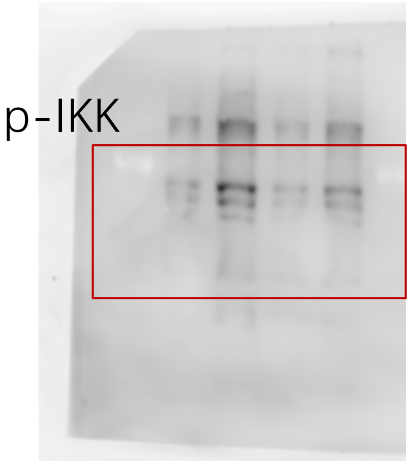
p-IKK

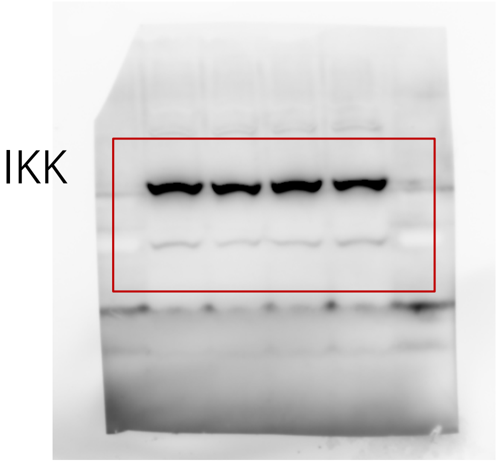
IKK

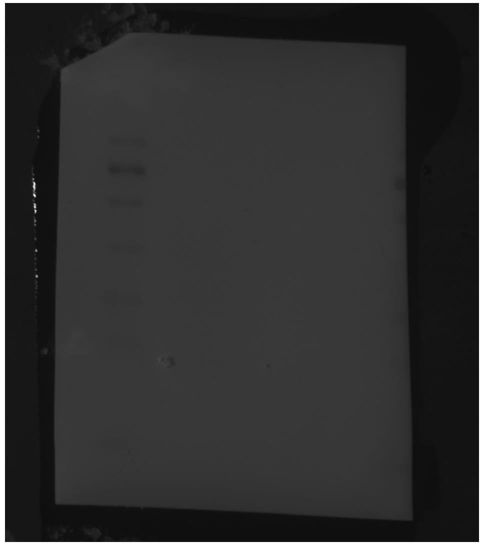

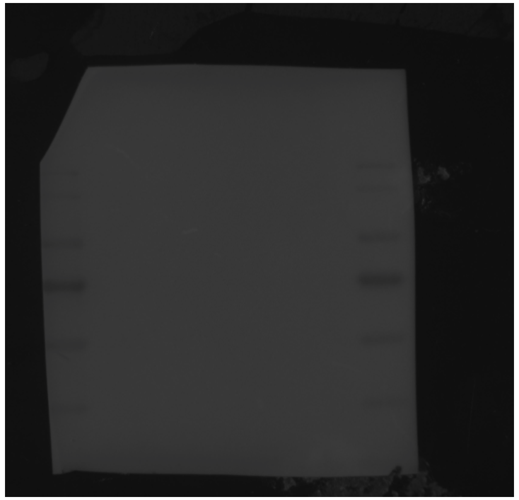

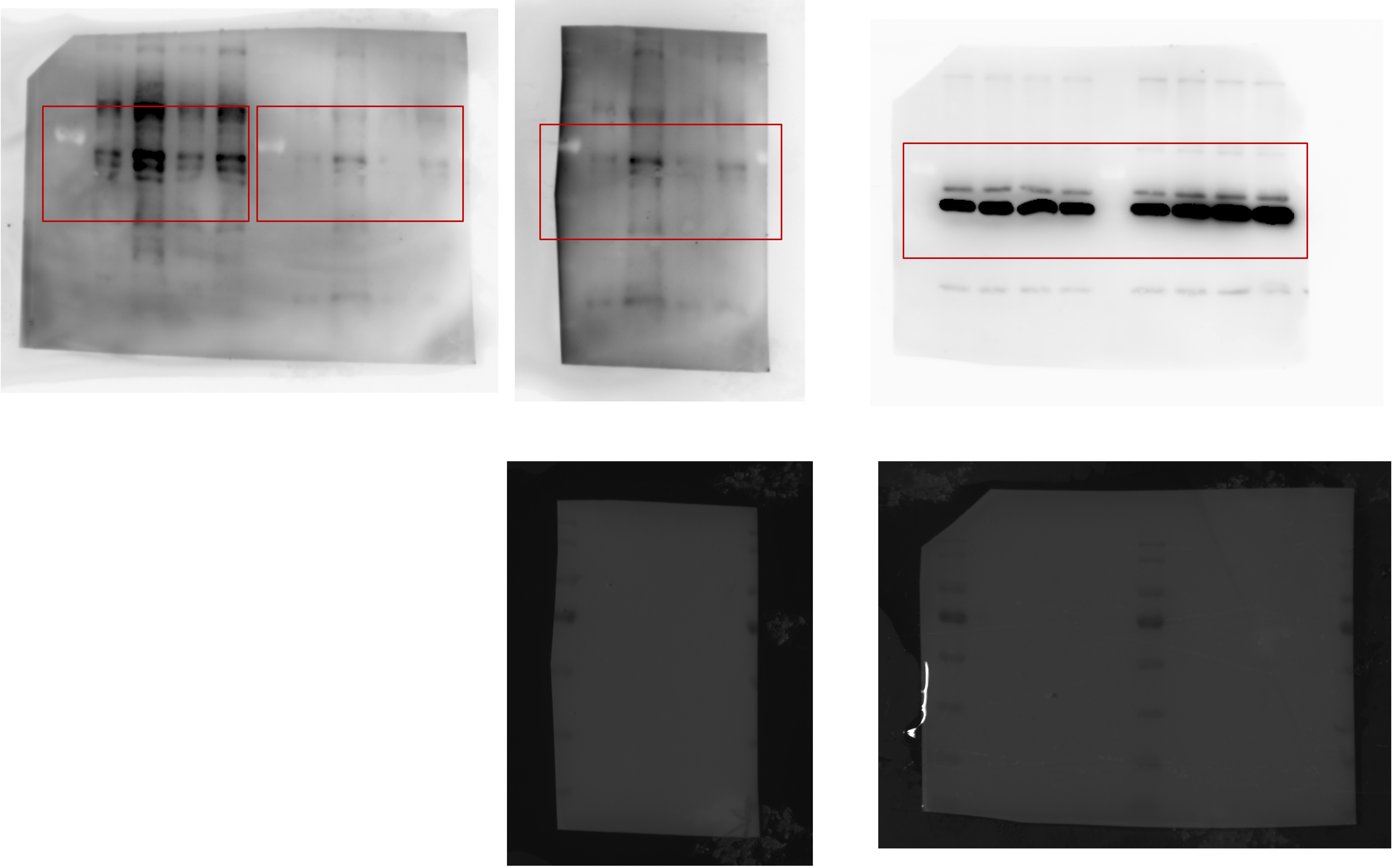
p-IKK
IKK

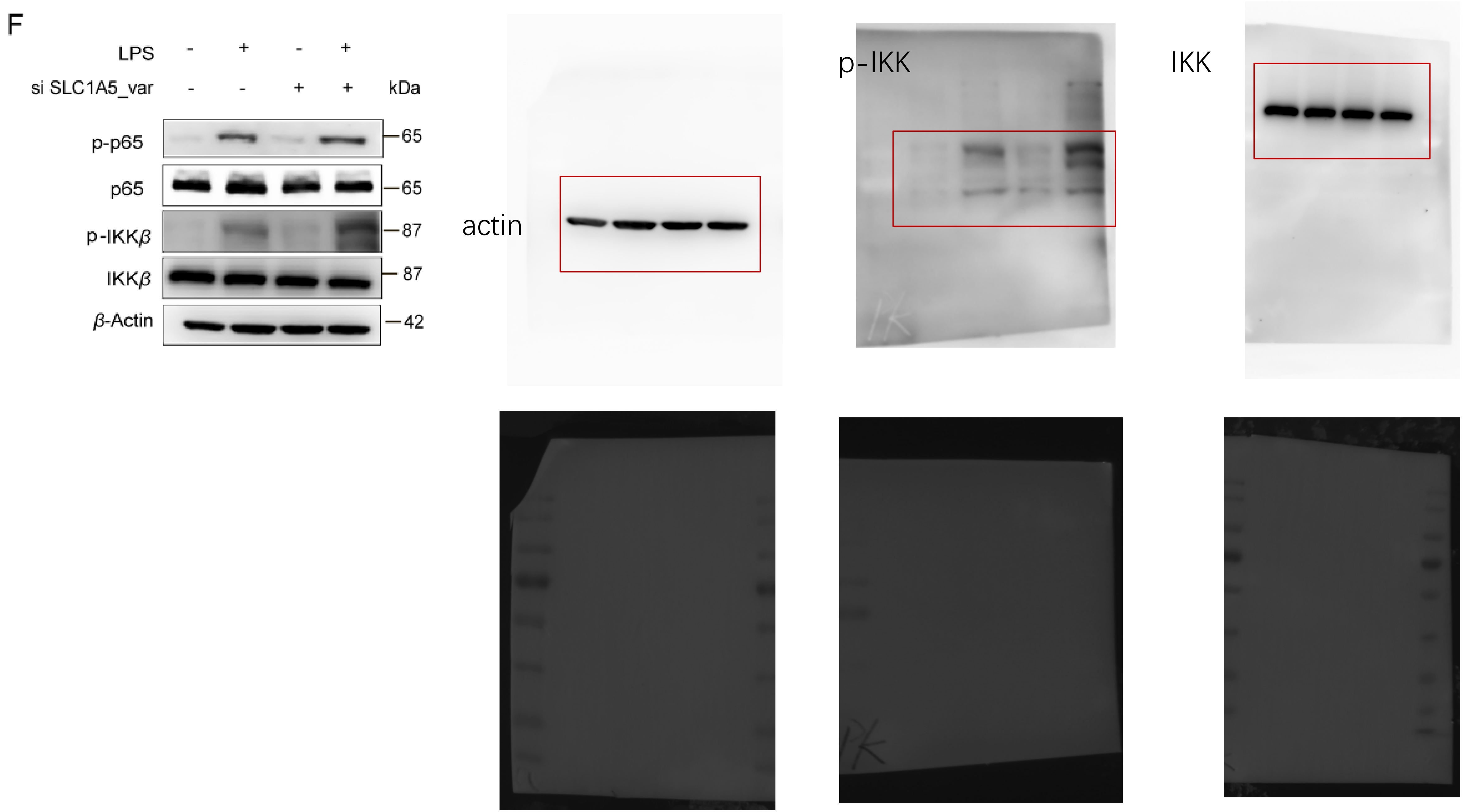
F
LPS - + - +
si SLC1A5_var - - + +
kDa
p-p65
65
p65
65
p-IKKβ
87
IKKβ
87
β-Actin
42
actin
p-IKK
IKK

p-IKK

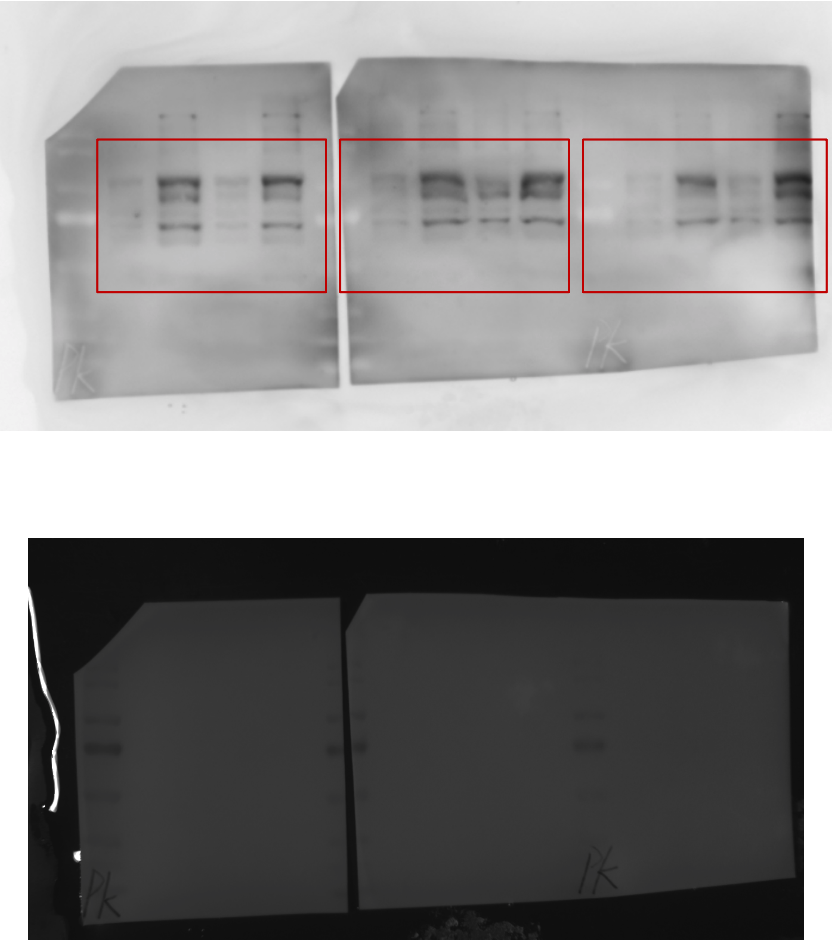

IKK

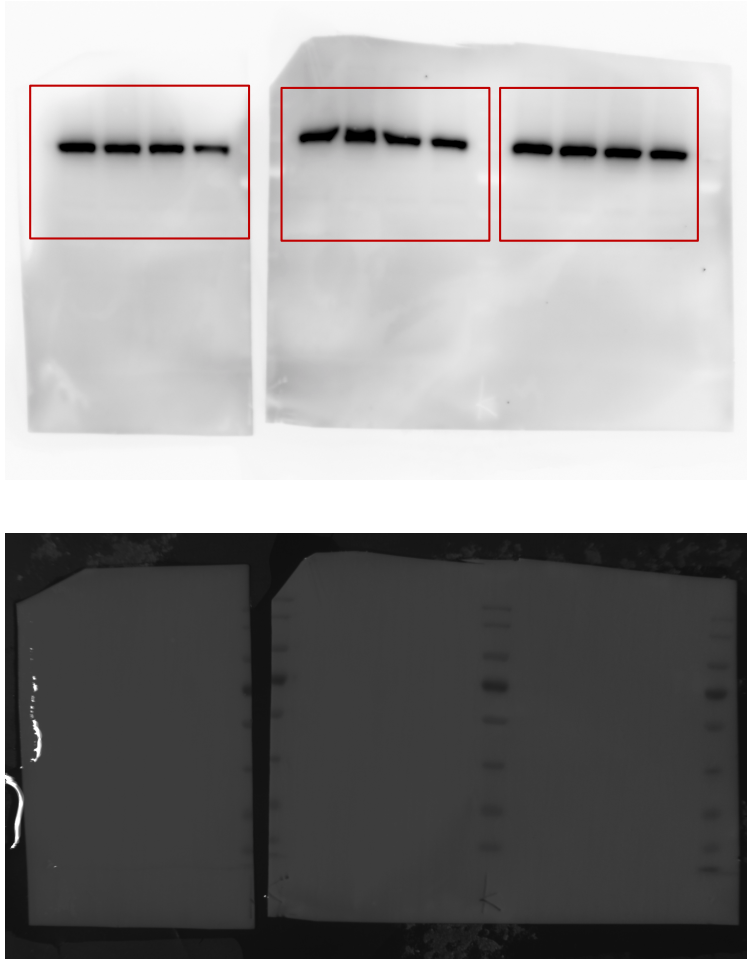

actin

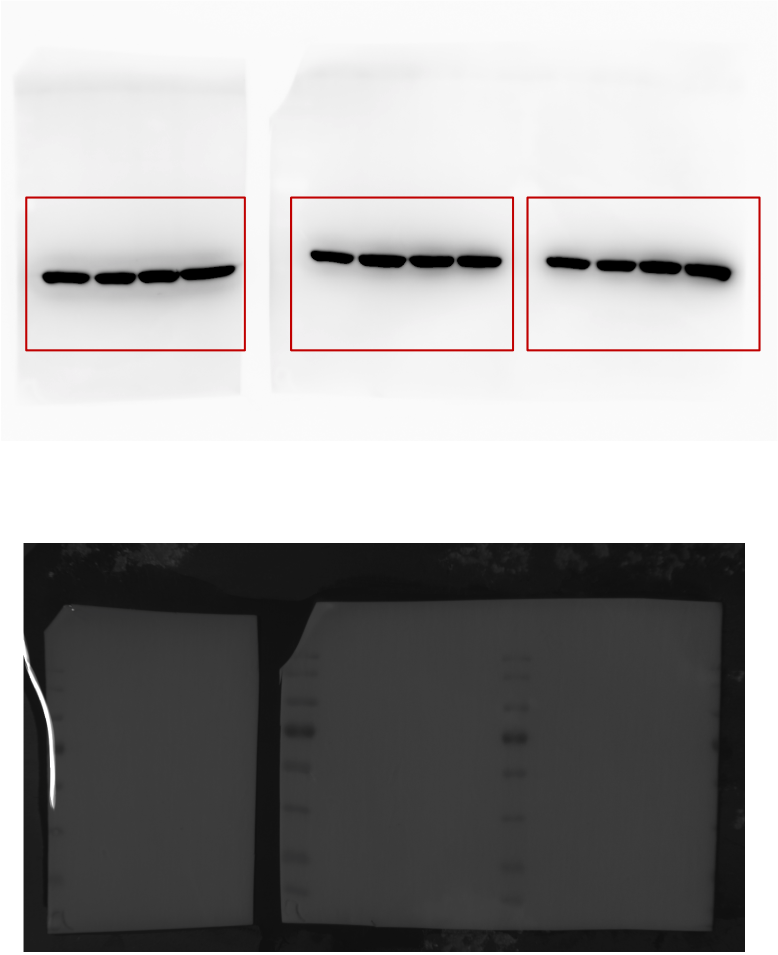

Figure 5

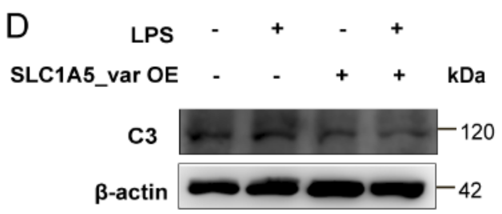

actin

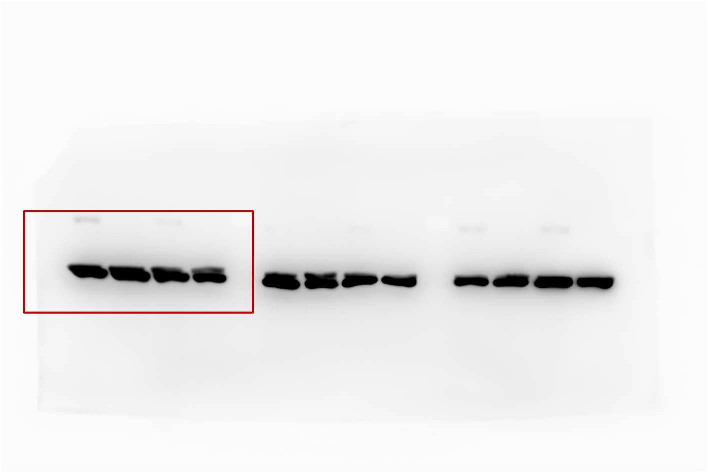

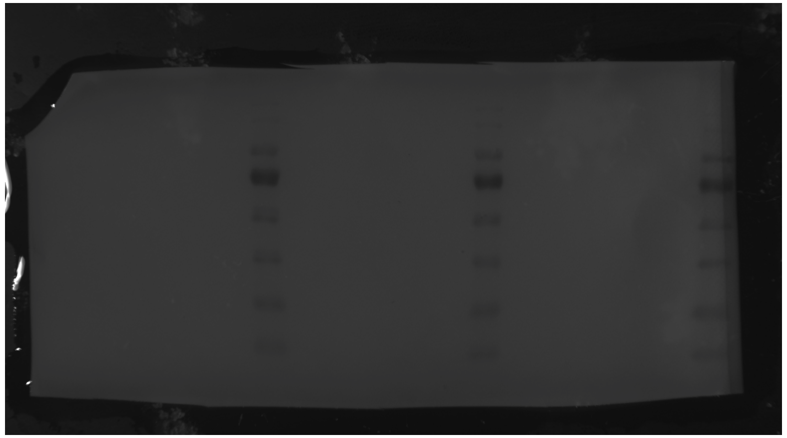

C3

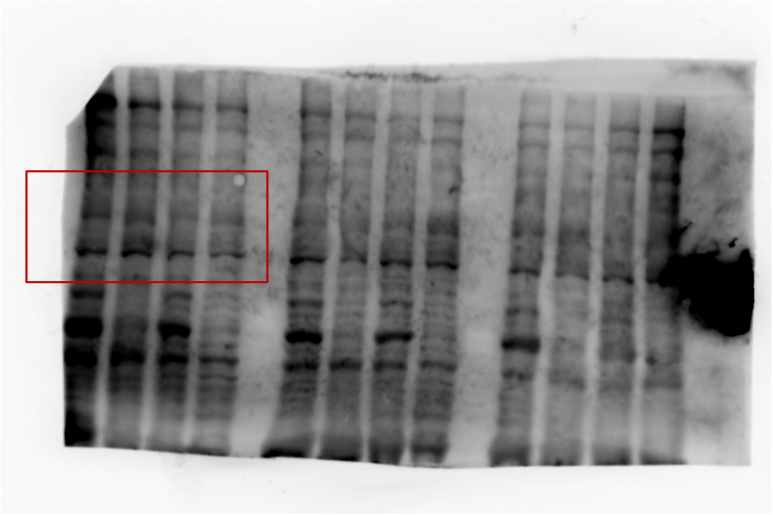

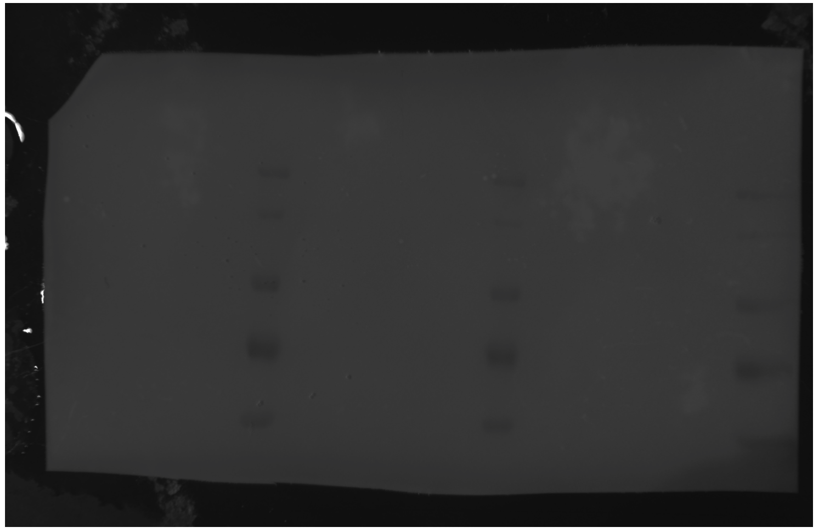

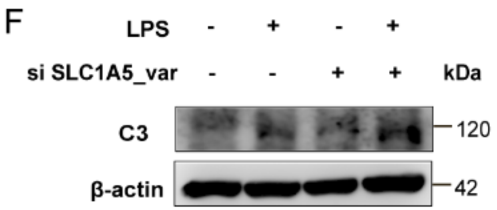

actin

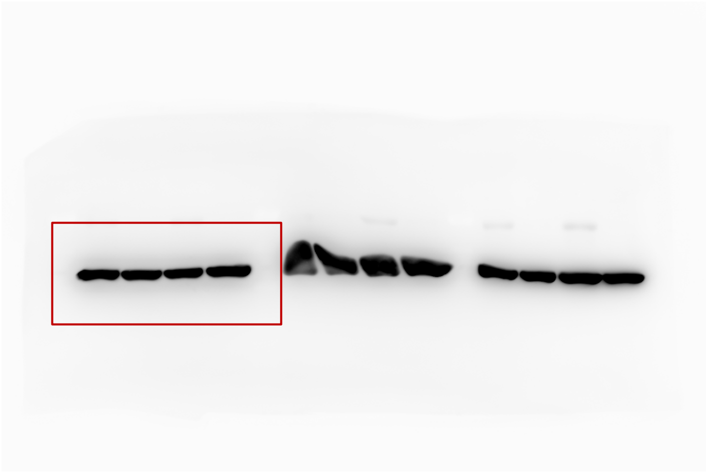

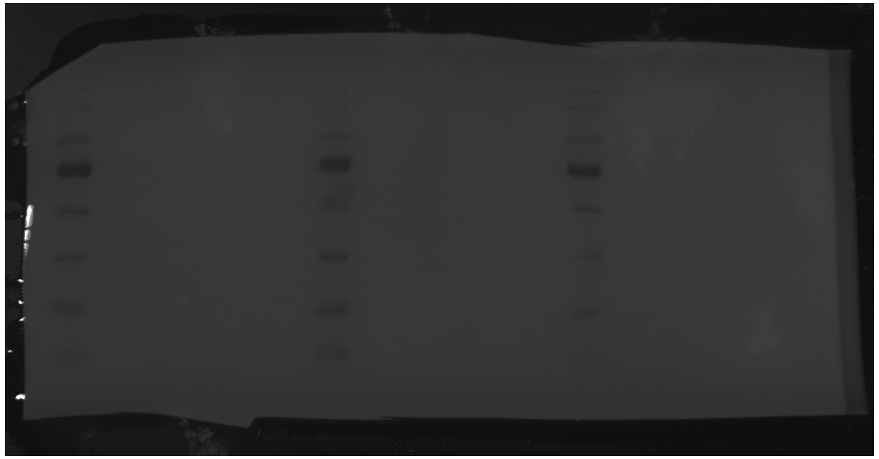

C3

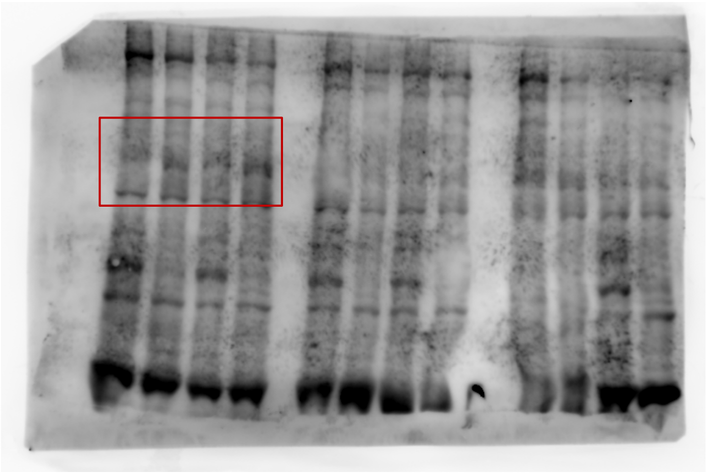

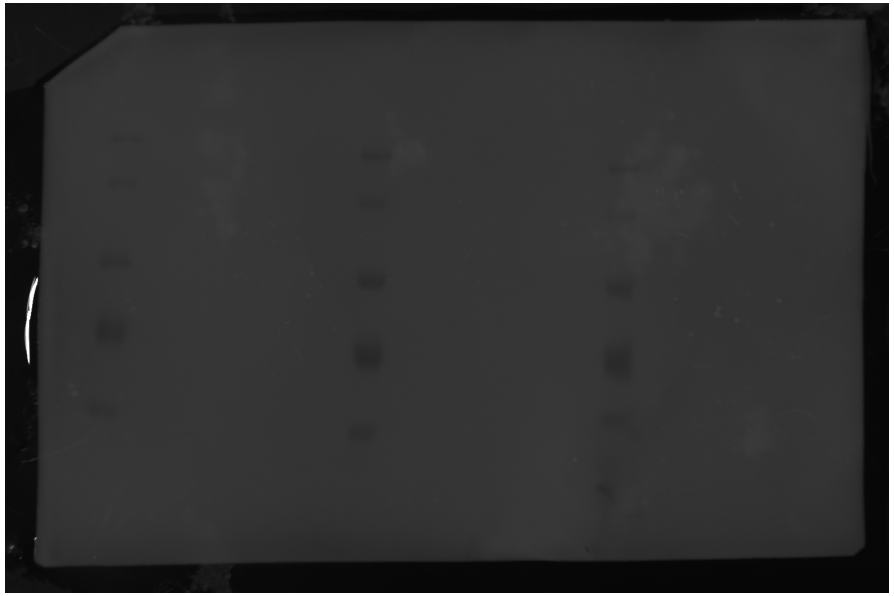

Figure 6

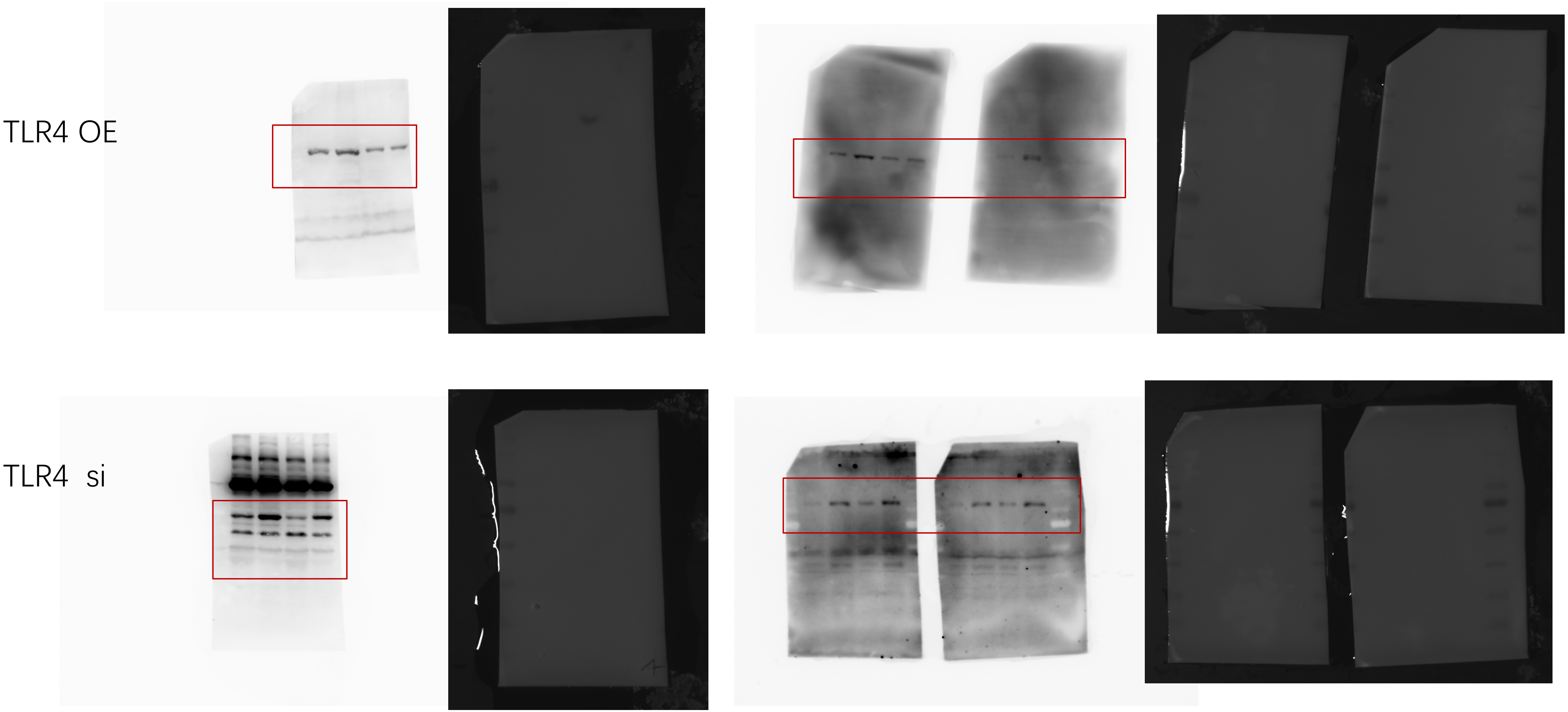

Figure 6

N=1

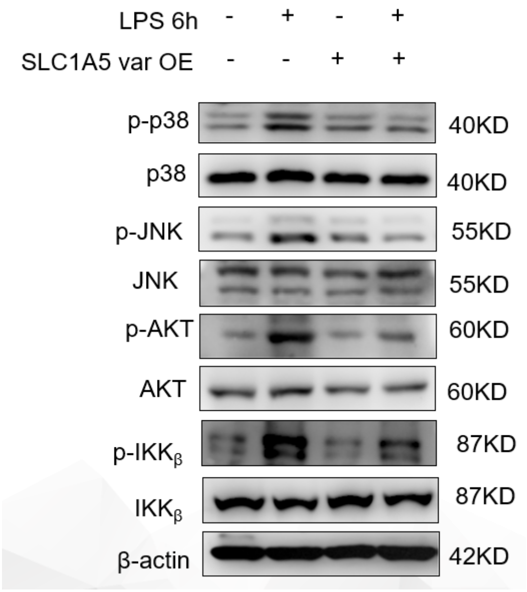

actin

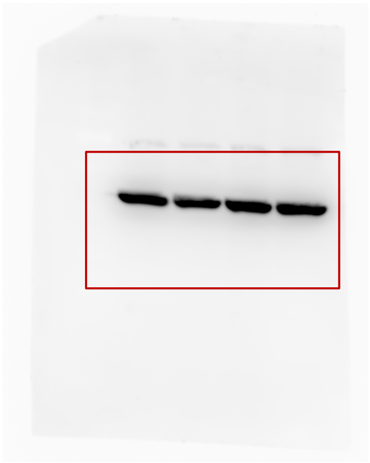

AKT

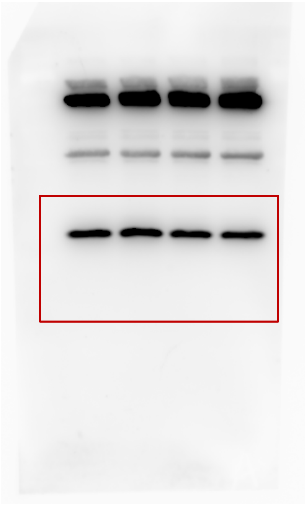

p-AKT

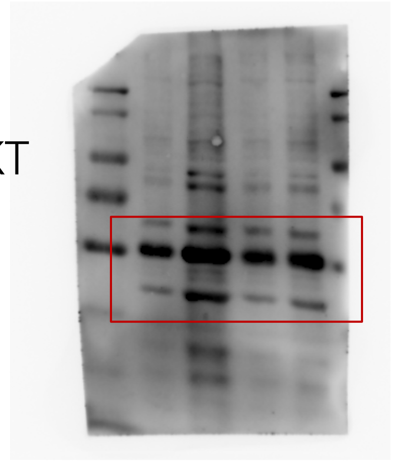

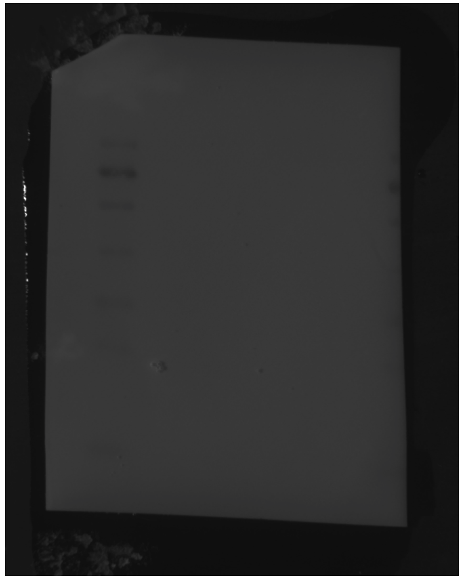

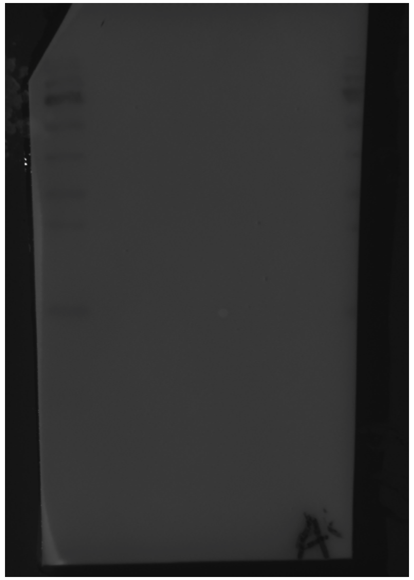

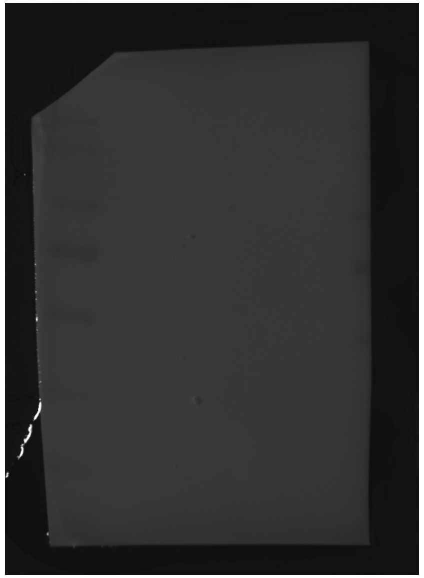

JNK

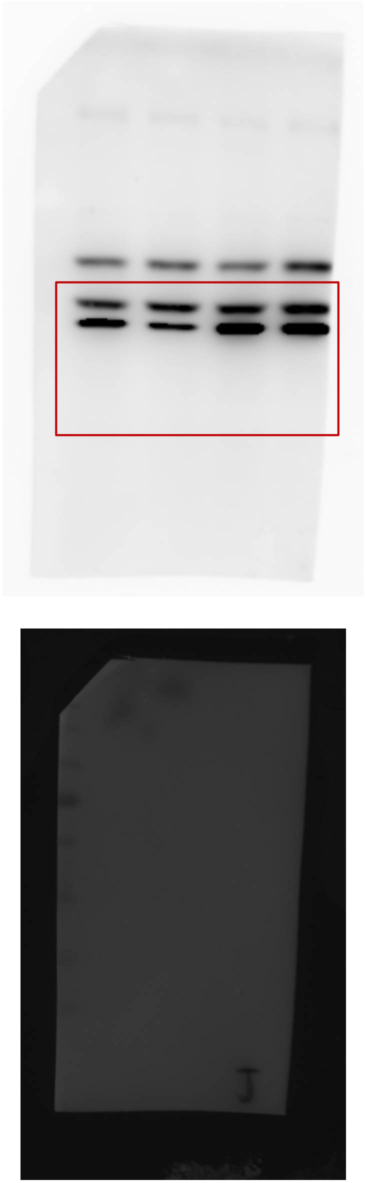

p-JNK

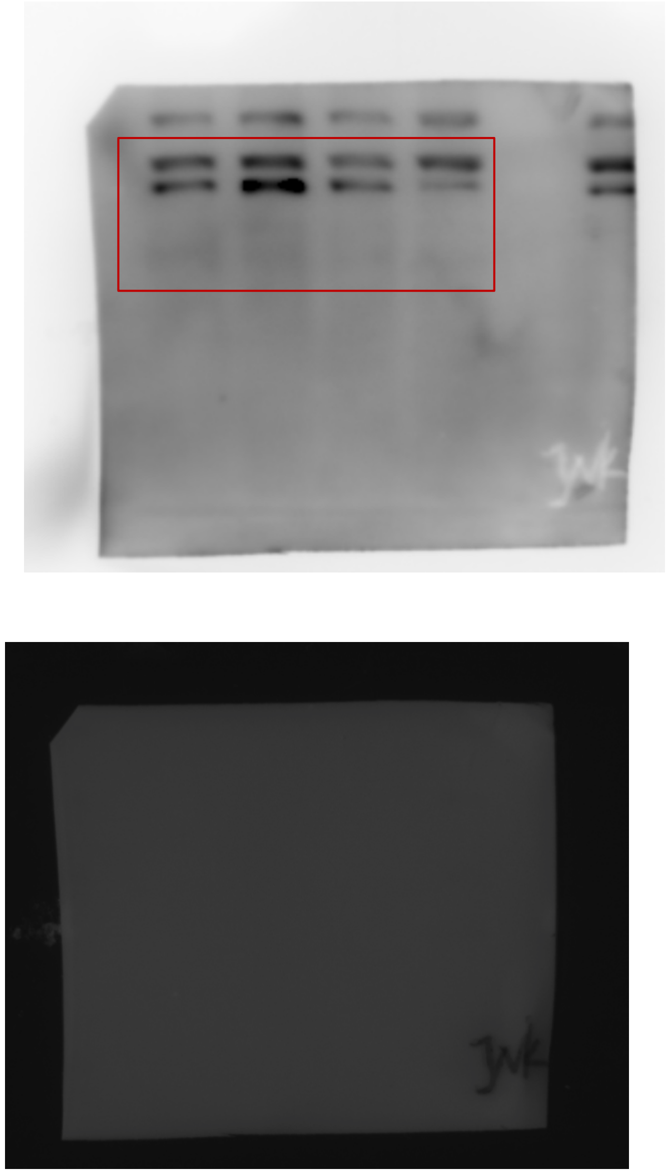

p38

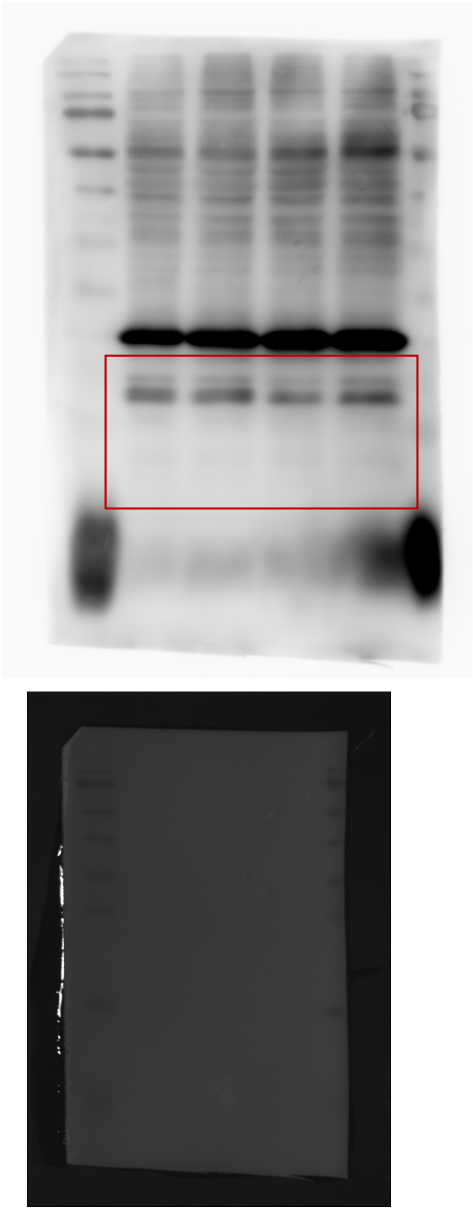

p-p38

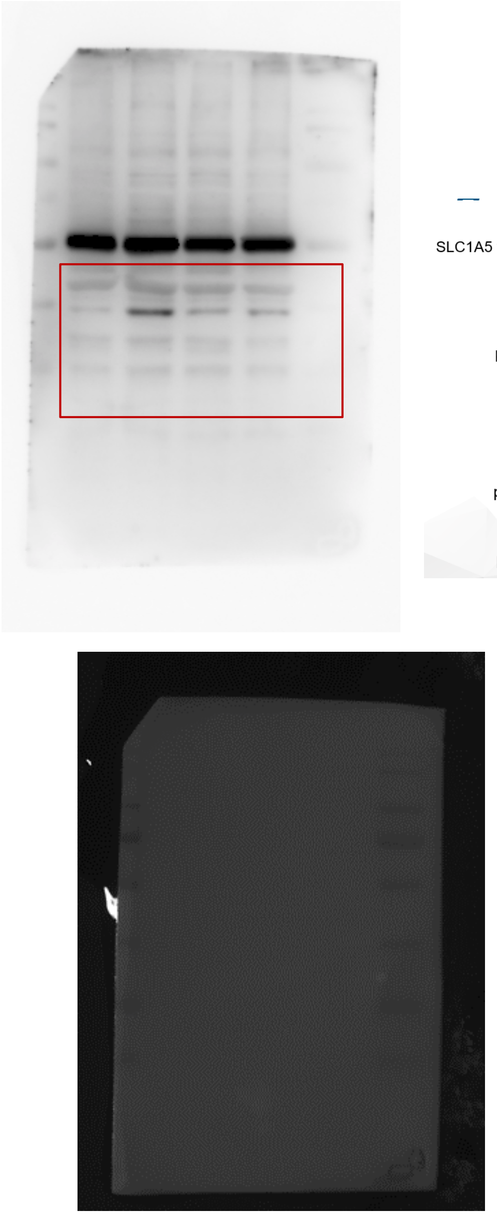

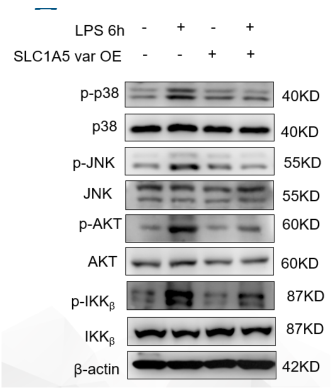

AKT

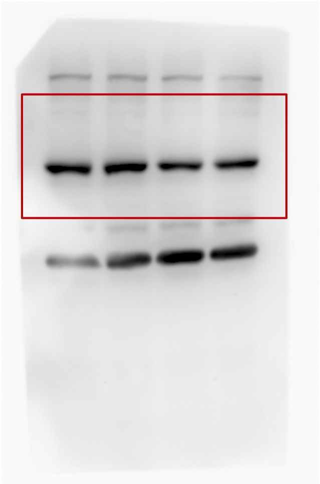

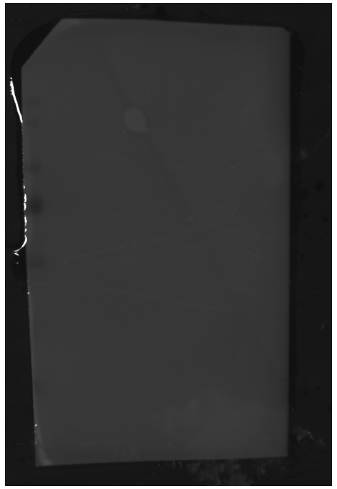

p-AKT（p-p38 strip）

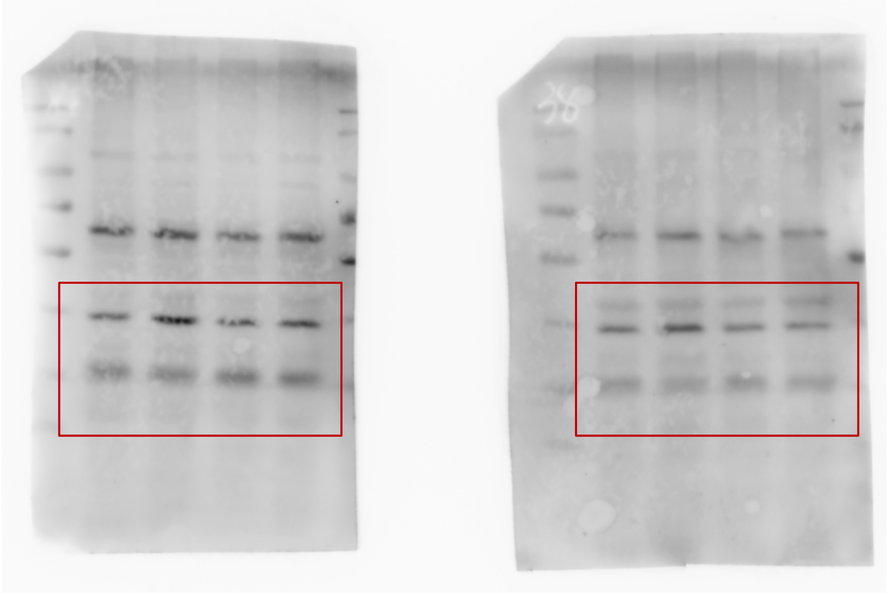

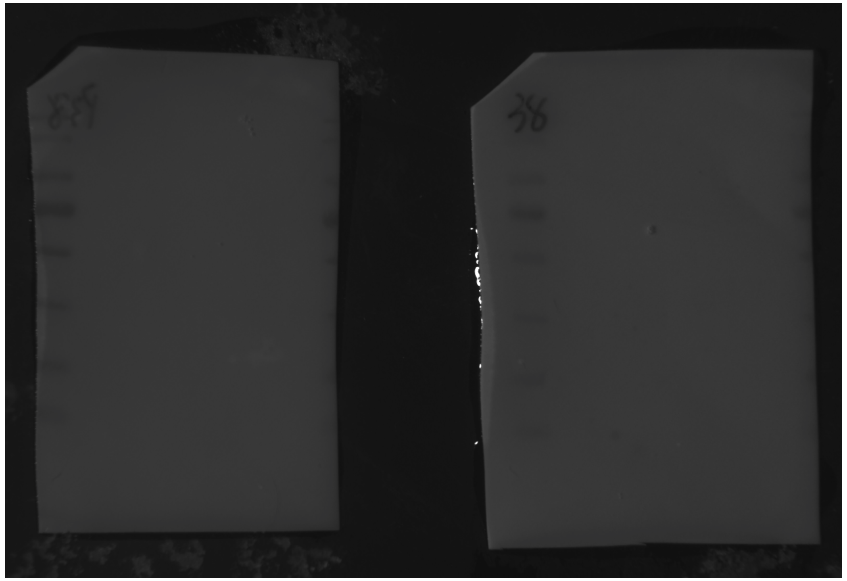

JNK

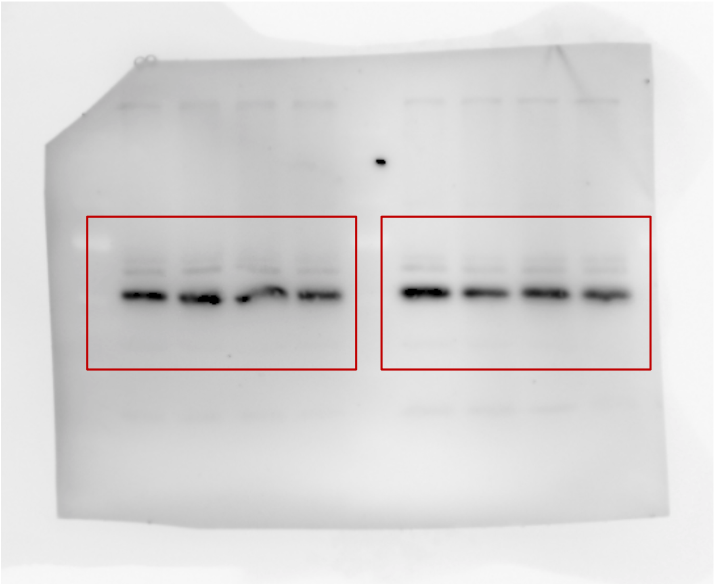

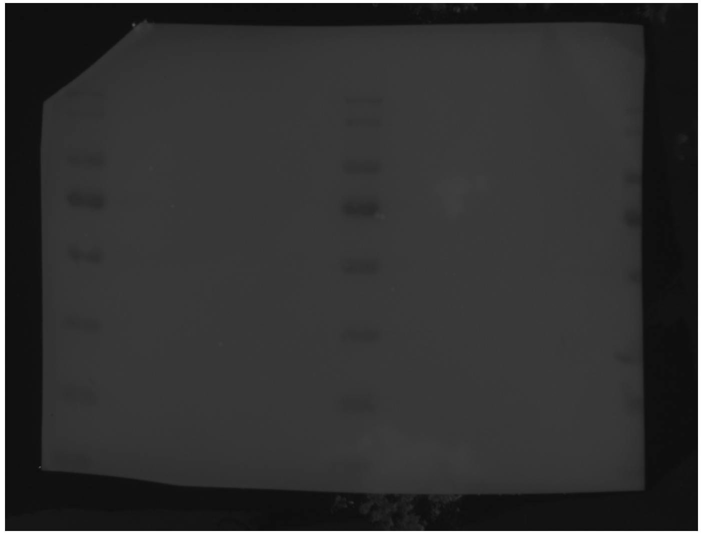

p-JNK

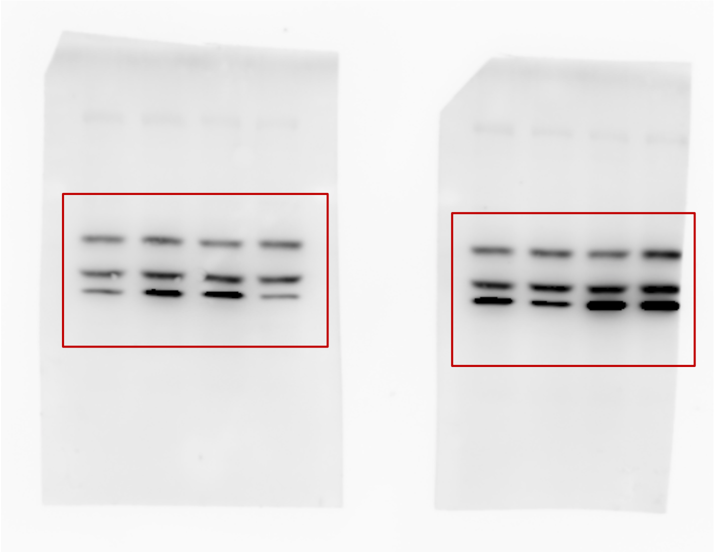

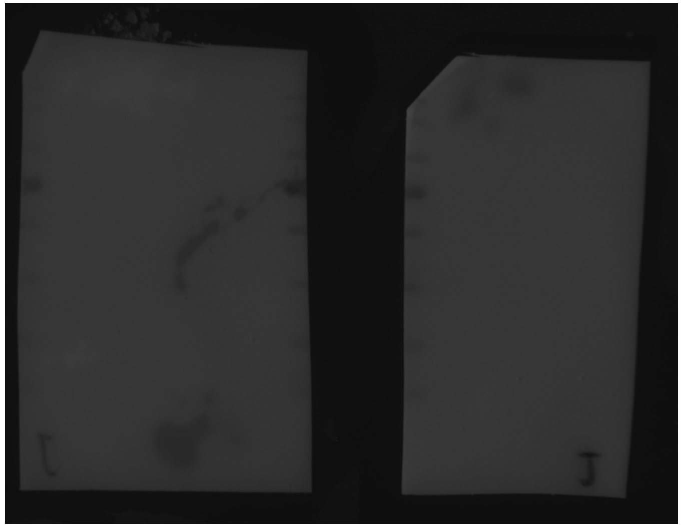

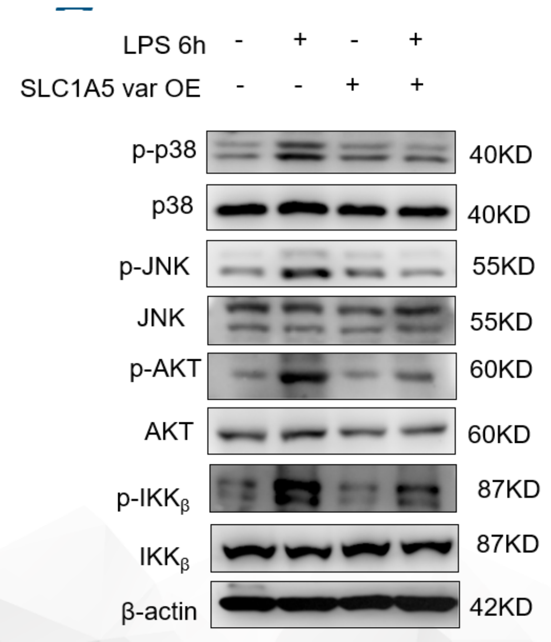
LPS 6h - + - +
SLC1A5 var OE - - + +
p-p38 40KD
p38 40KD
p-JNK 55KD
JNK 55KD
p-AKT 60KD
AKT 60KD
p-IKKβ 87KD
IKKβ 87KD
β-actin 42KD

p38

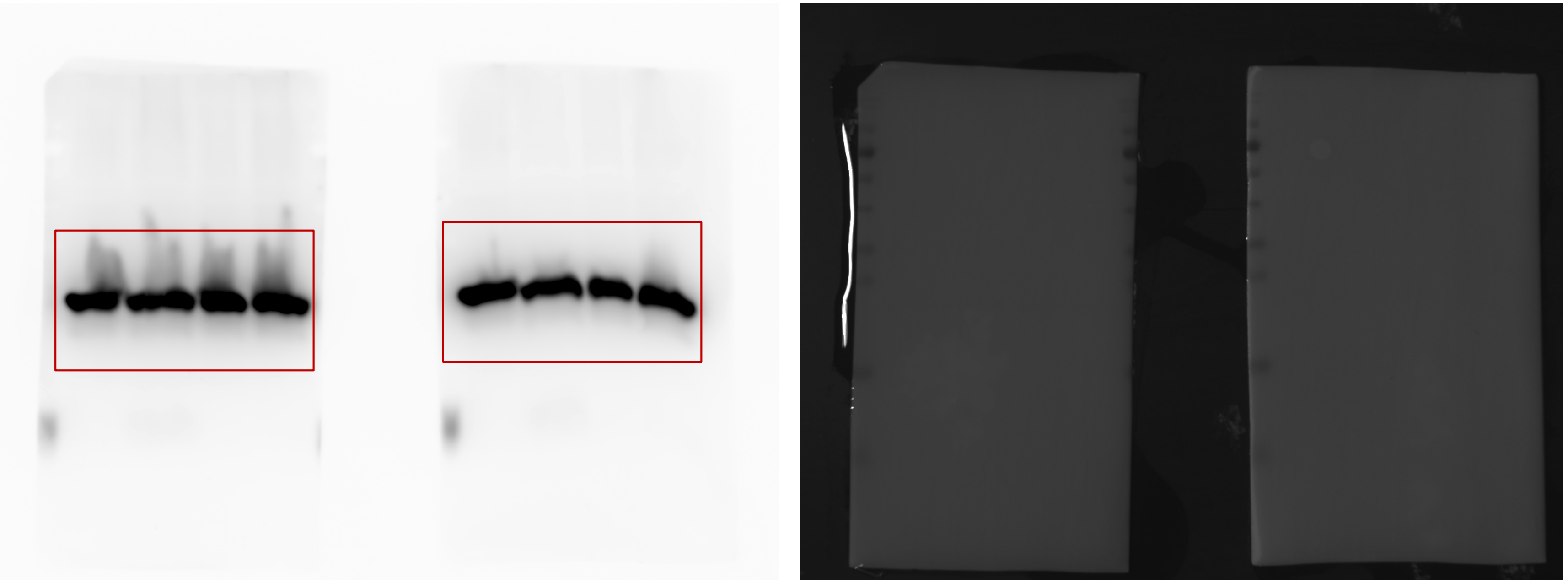

p-p38

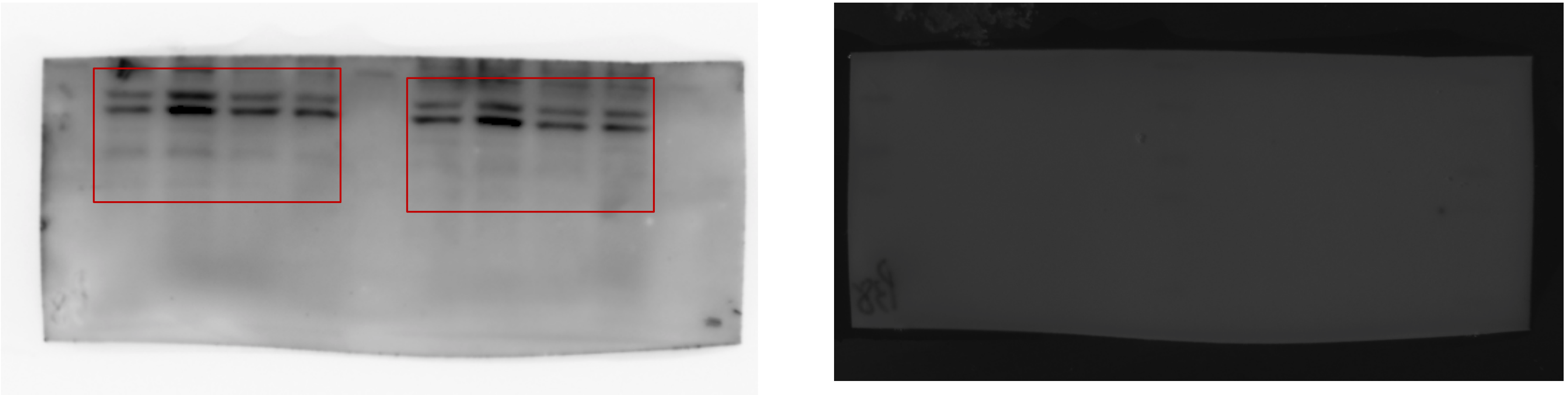

Figure 6

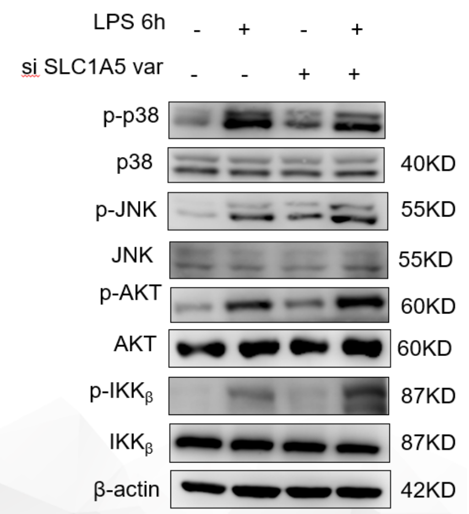

actin

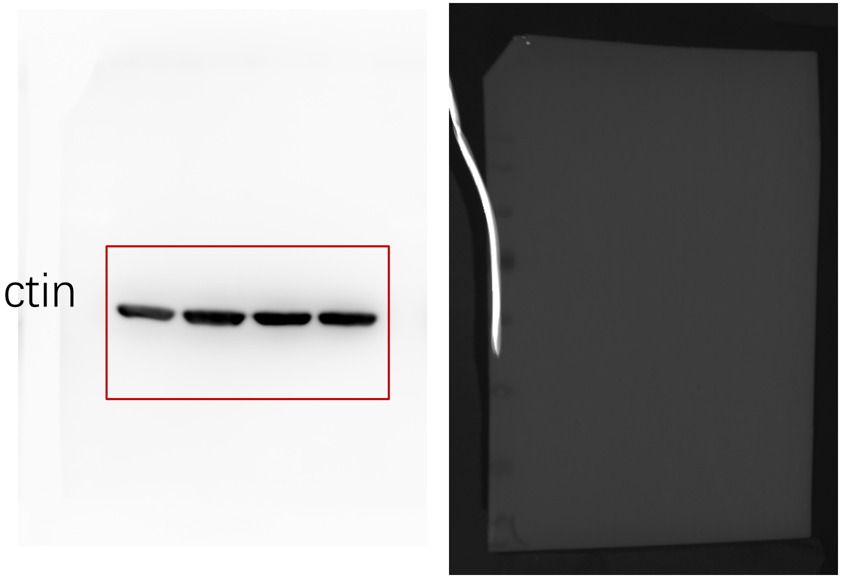

AKT

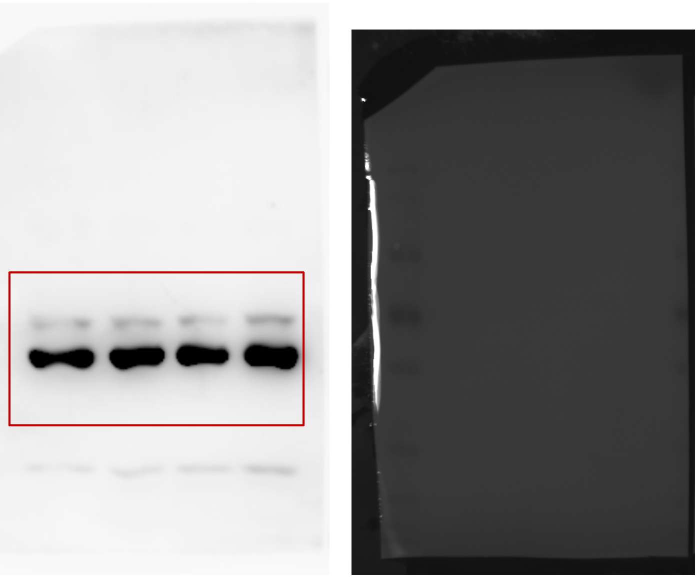

p-AKT

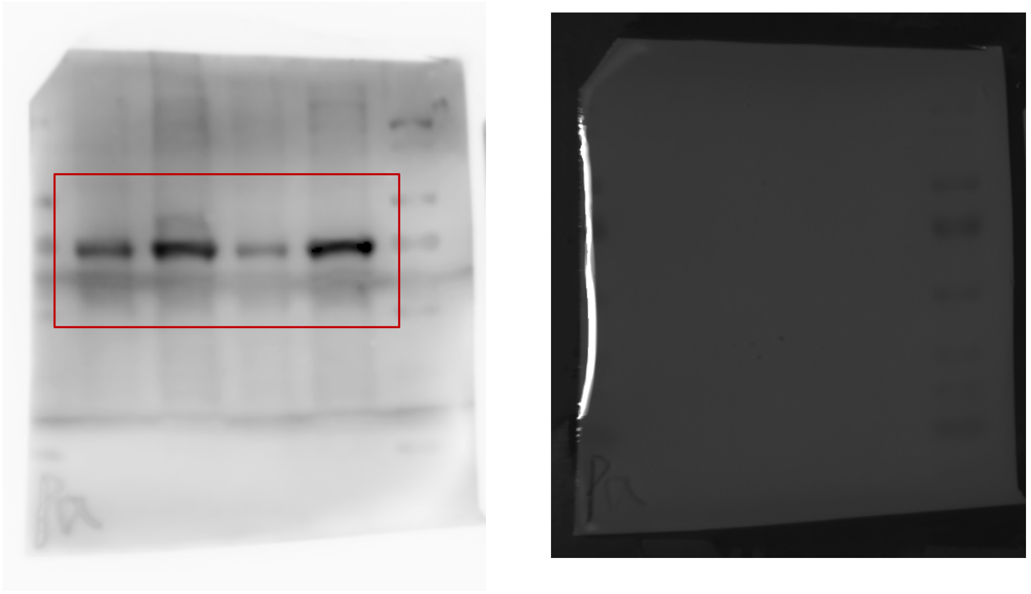

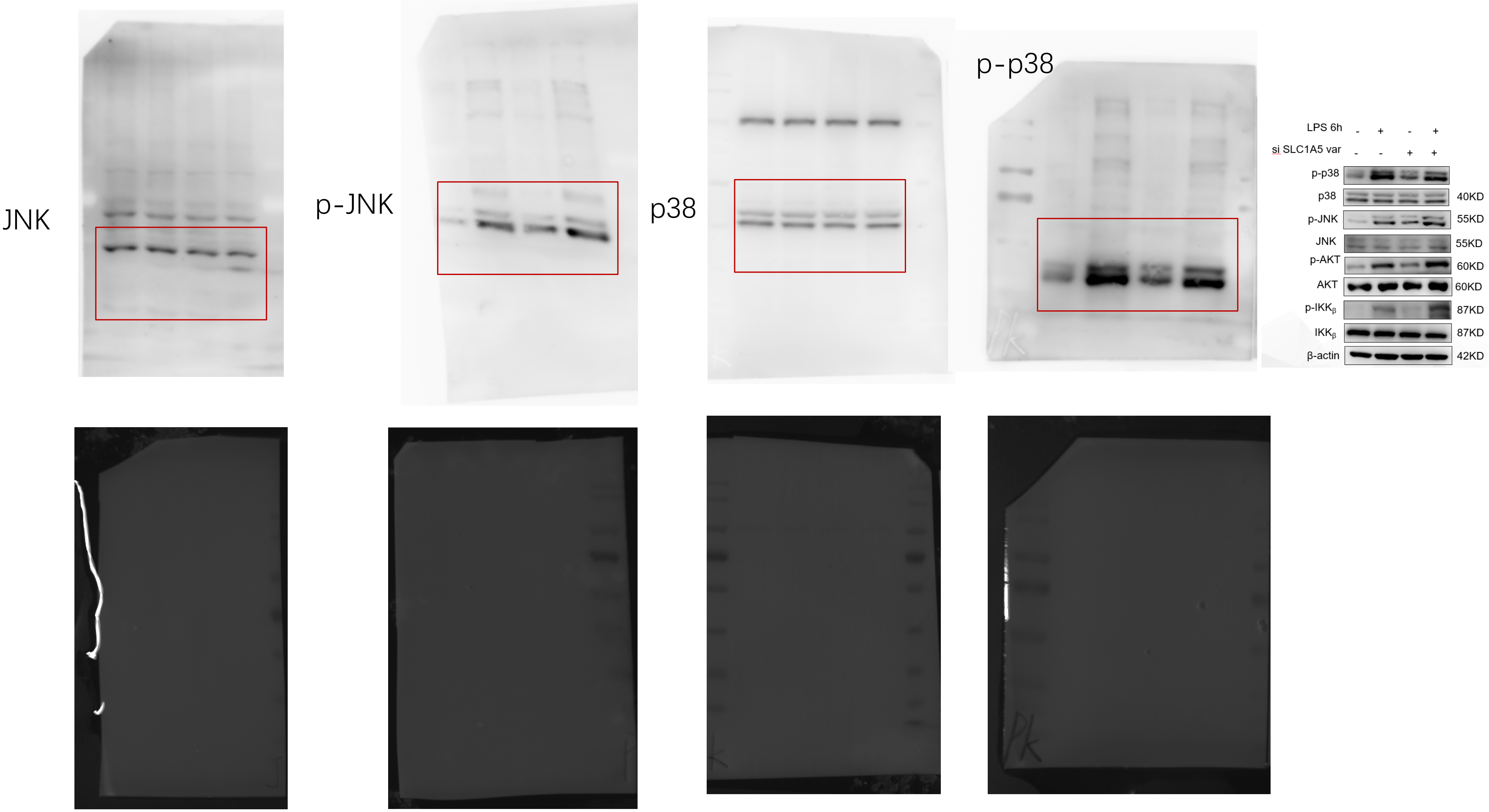
JNK
p-JNK
p38
p-p38
LPS 6h - + - +
si SLC1A5 var - - + +
p-p38
p38 40KD
p-JNK 55KD
JNK 55KD
p-AKT 60KD
AKT 60KD
p-IKKβ 87KD
IKKβ 87KD
β-actin 42KD

actin

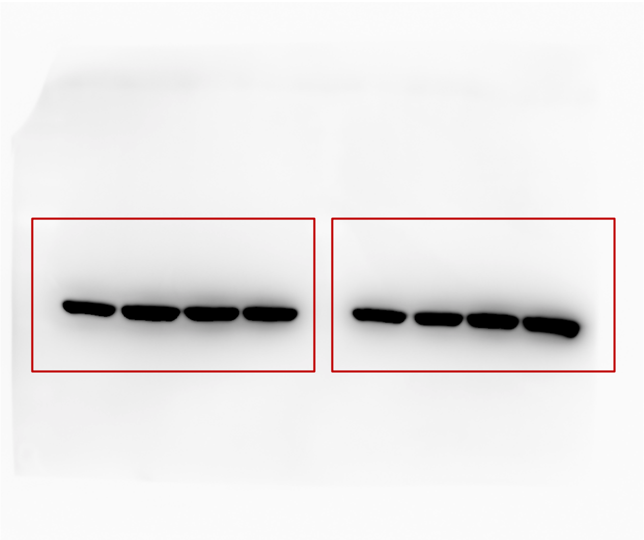

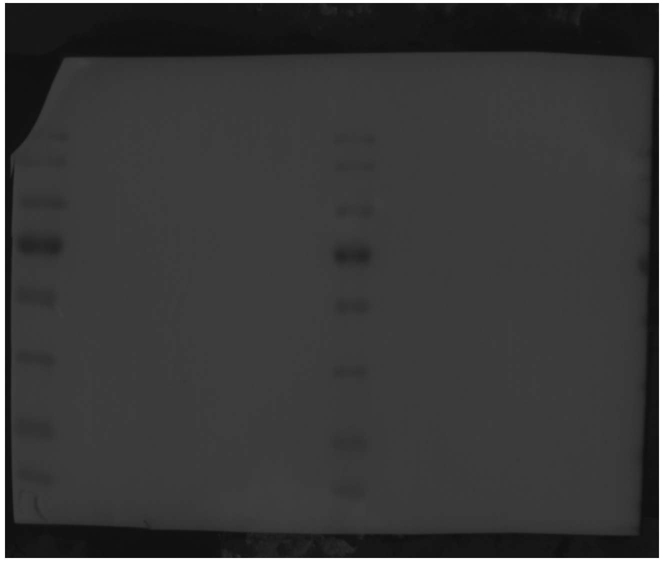

AKT

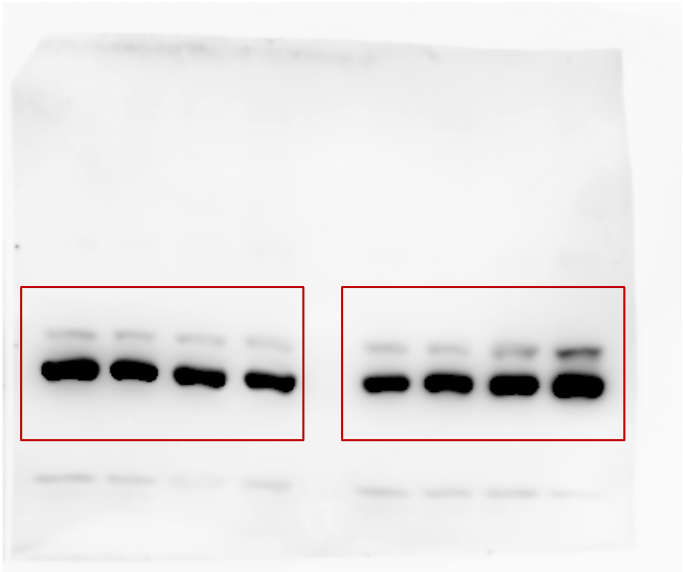

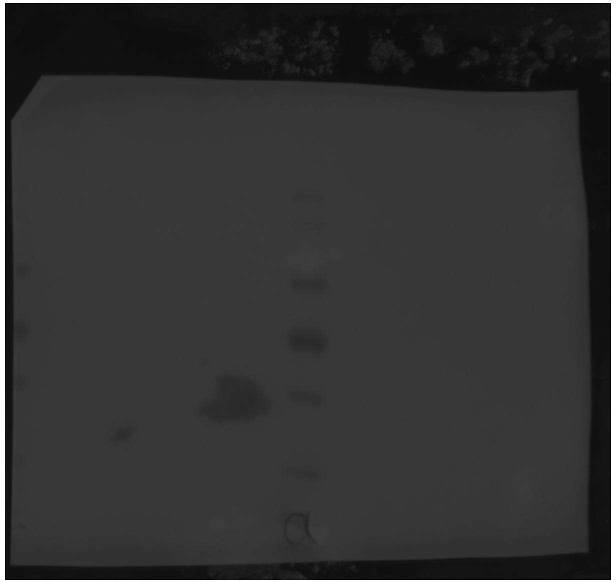

p-AKT

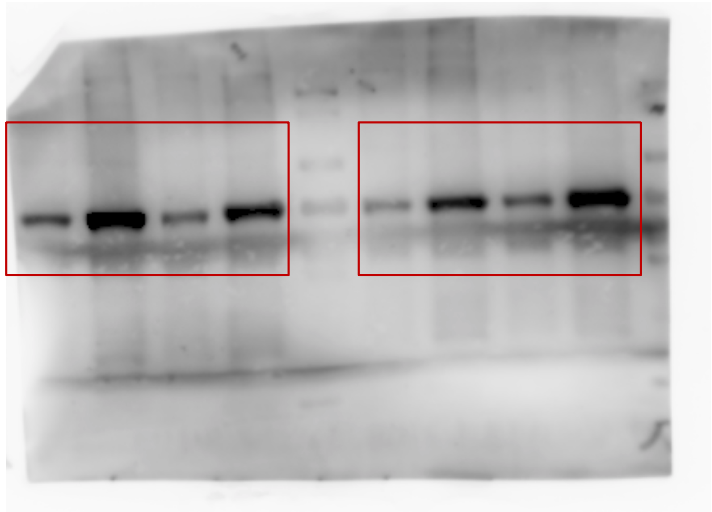

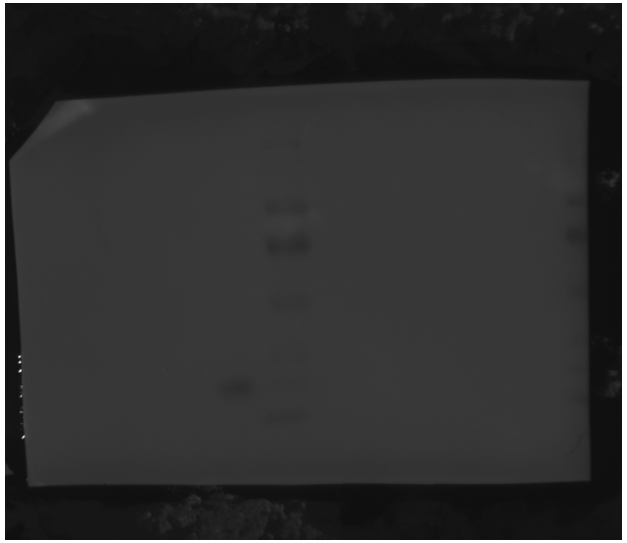

JNK

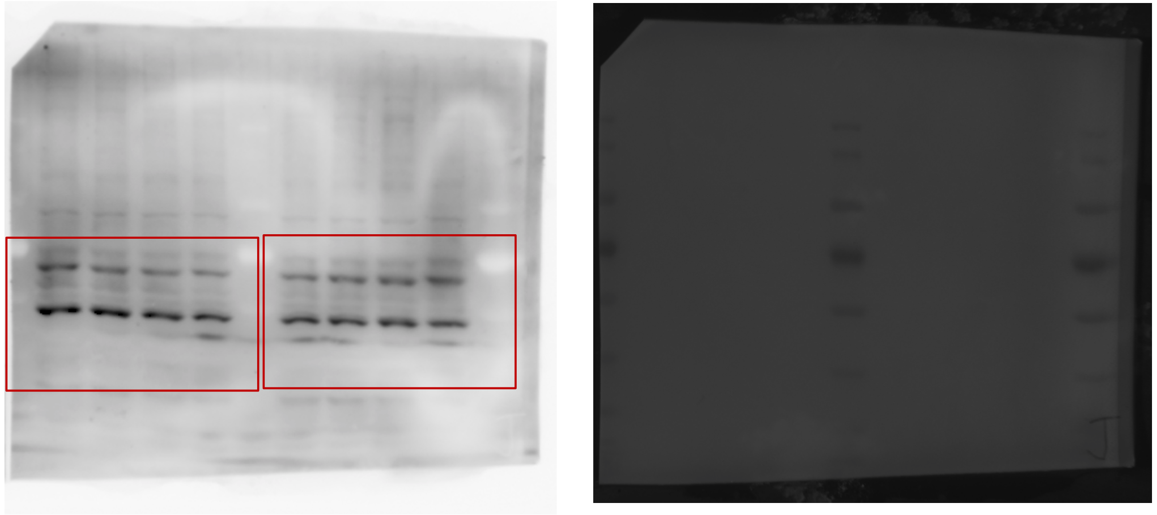

p38

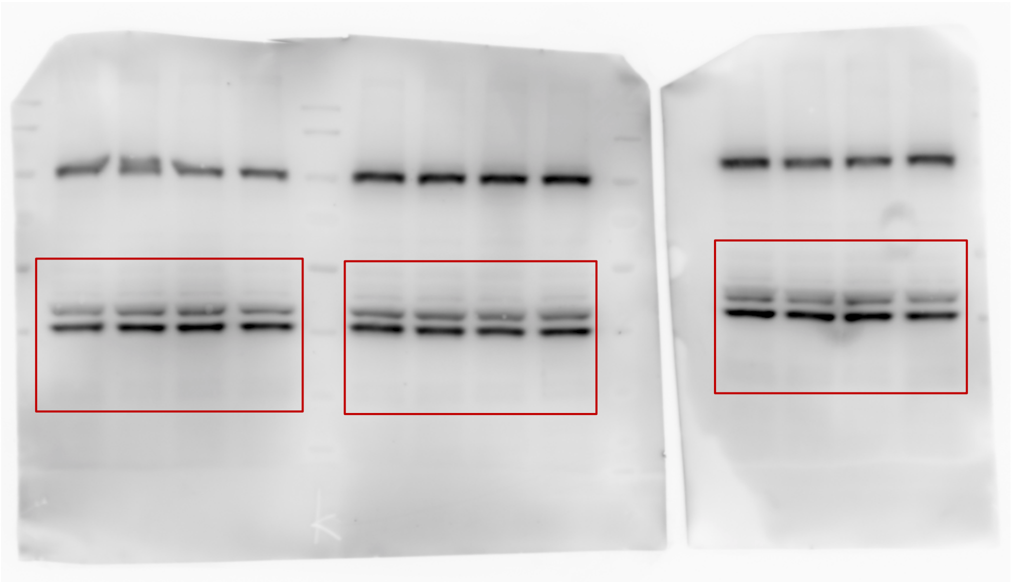

p-JNK

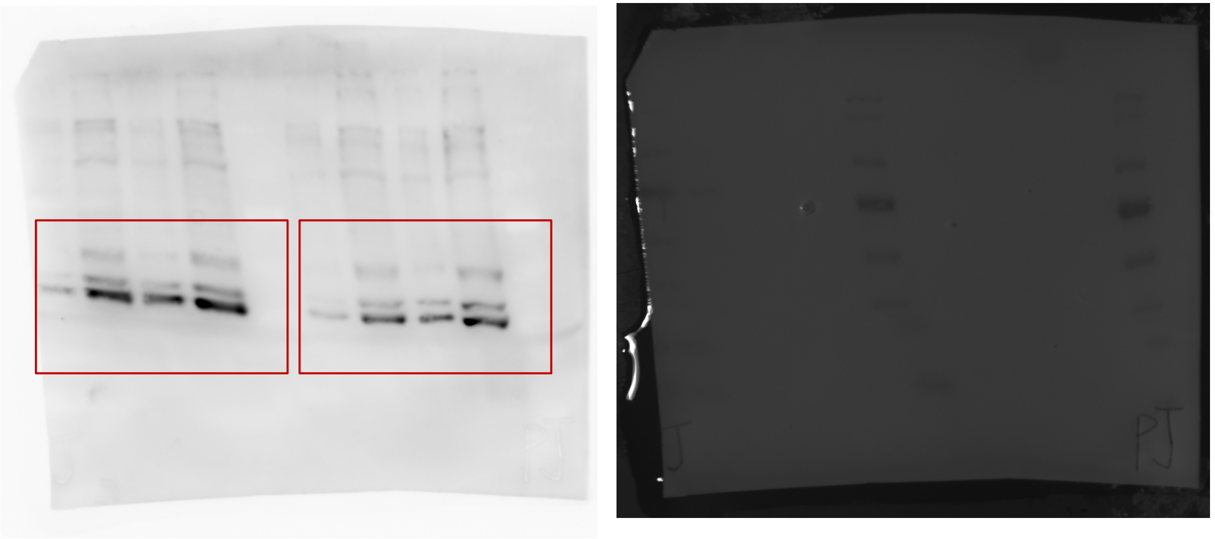

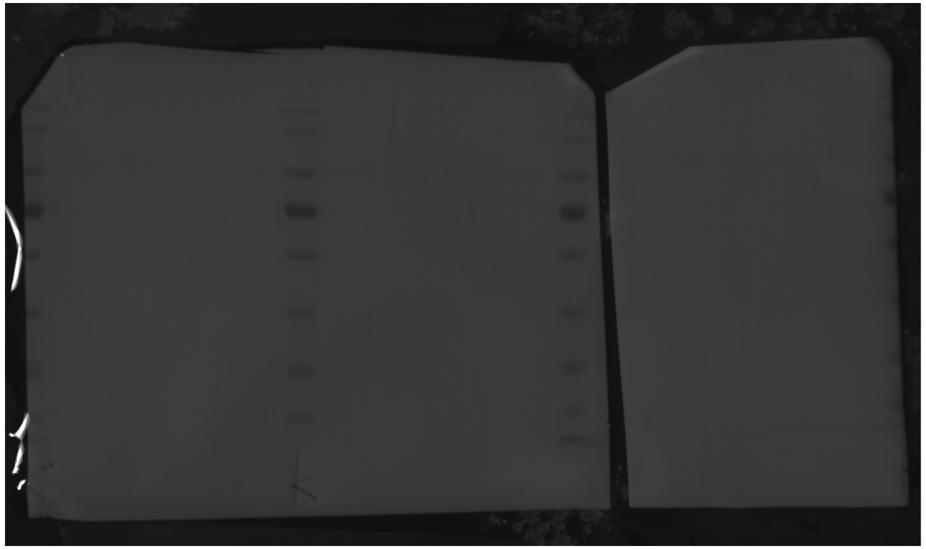

p-p38

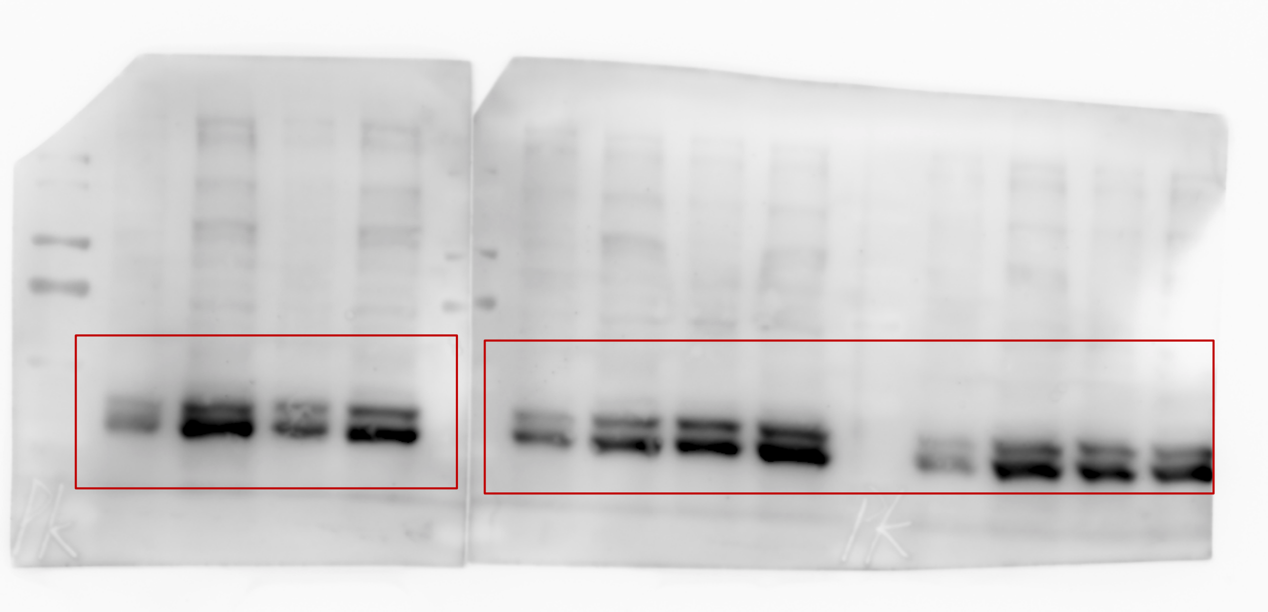

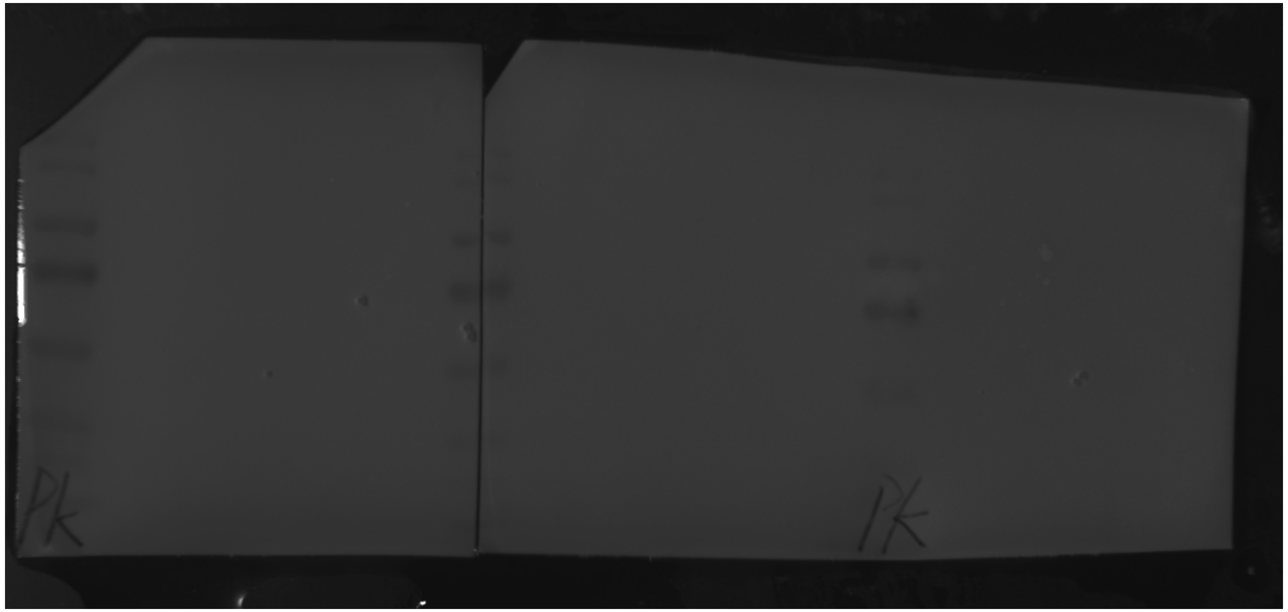

Figure 7

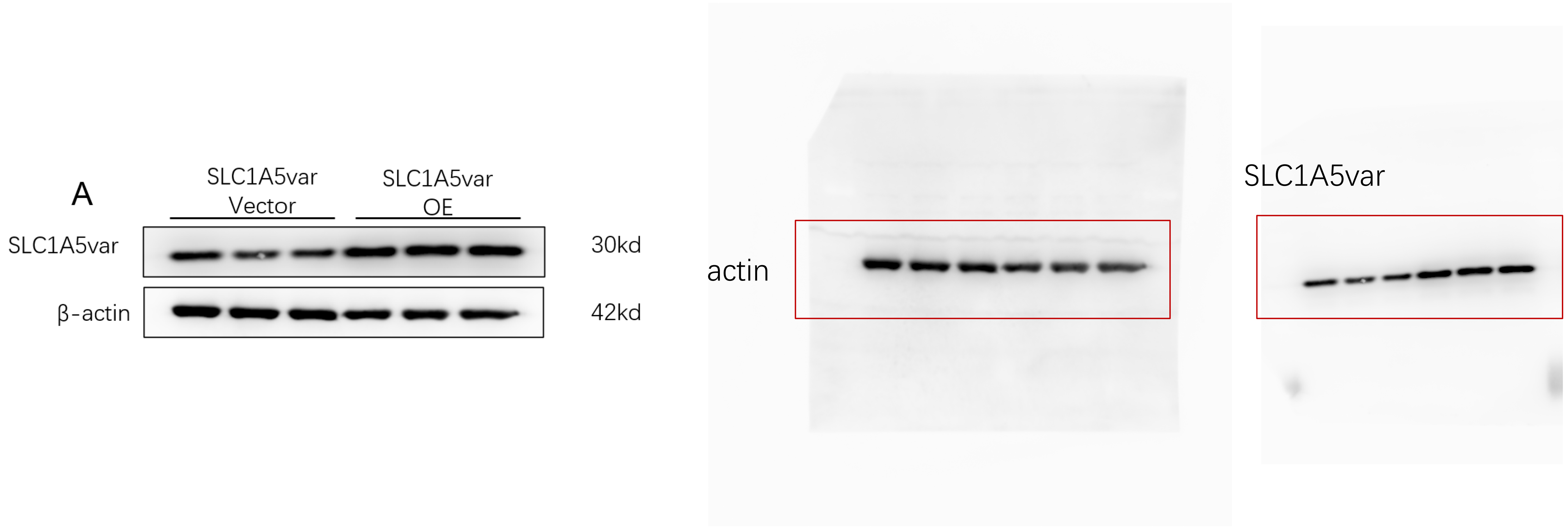
A
SLC1A5var Vector
SLC1A5var OE
SLC1A5var
β-actin
30kd
42kd
actin
SLC1A5var

Figure 8

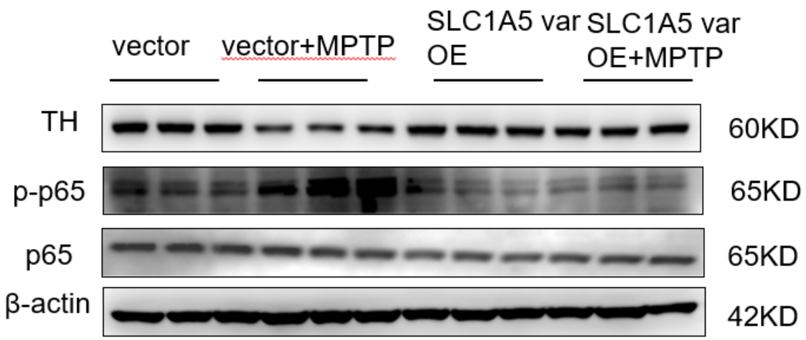

actin

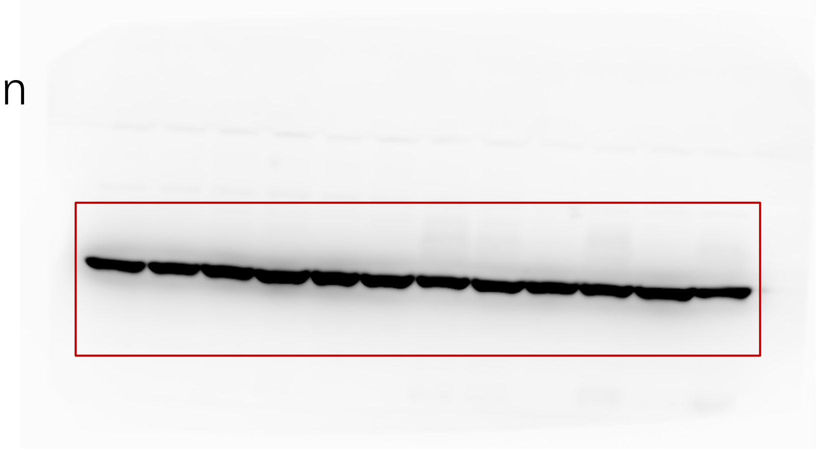

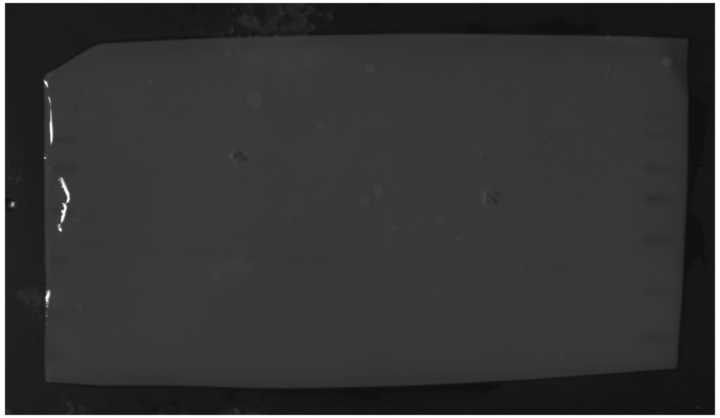

TH

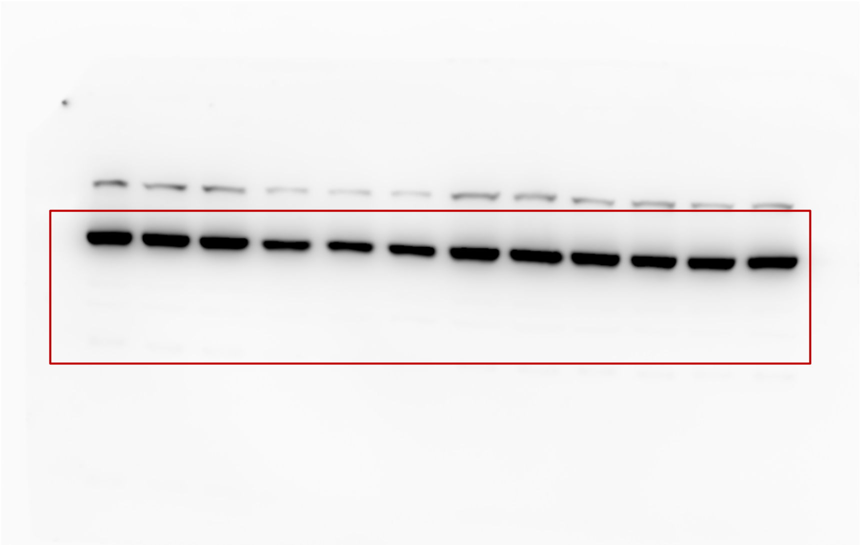

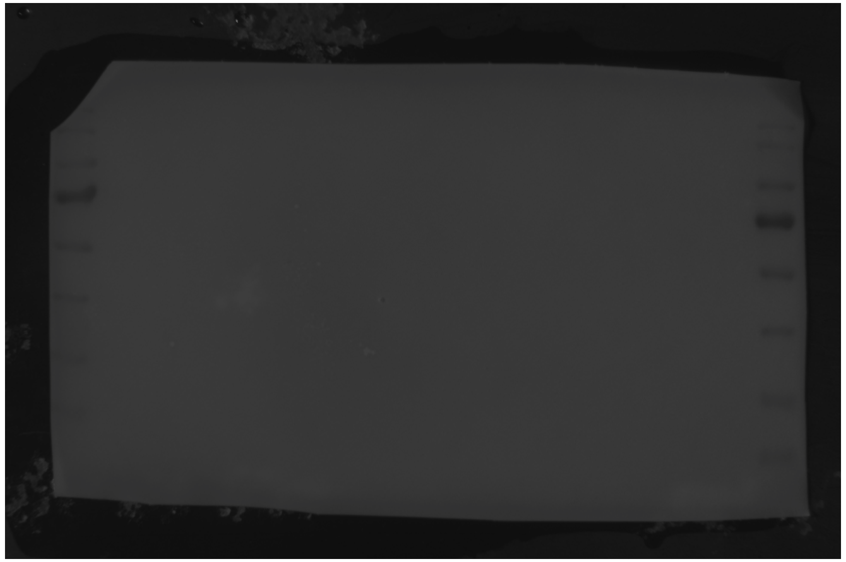

p65

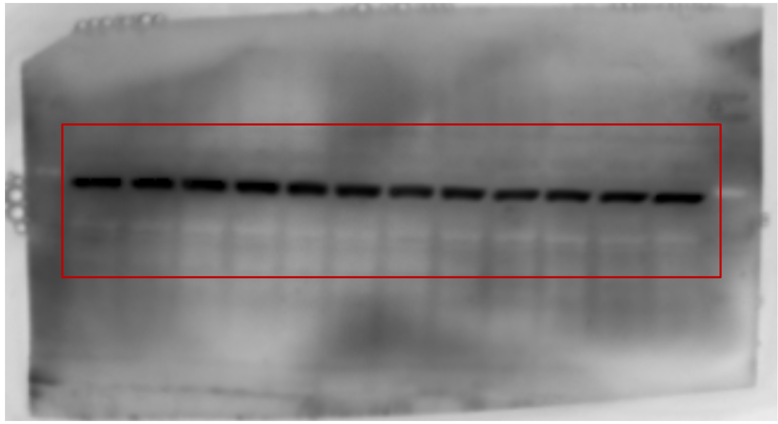

p-p65

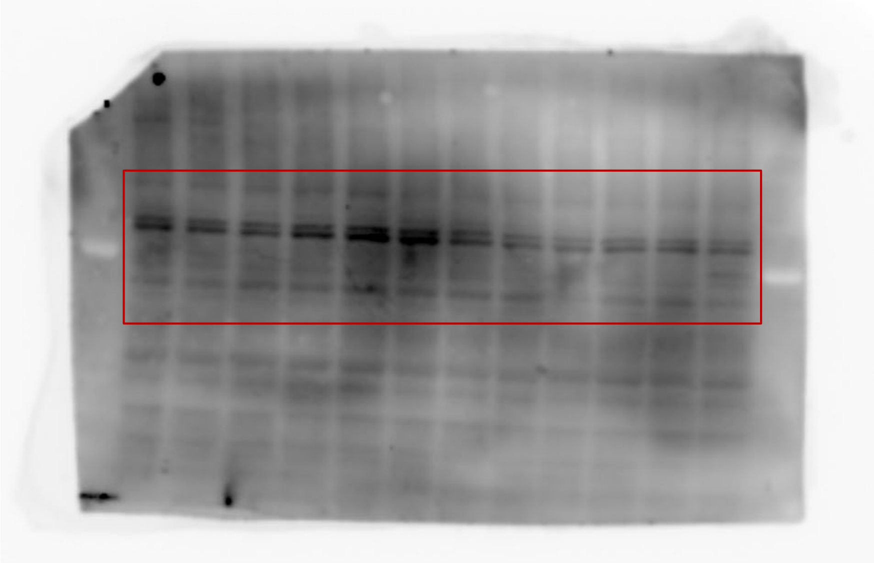

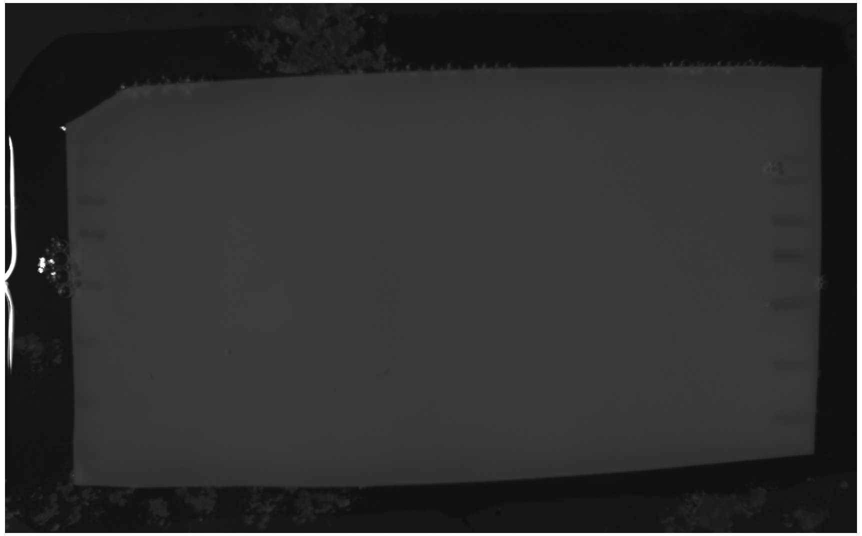

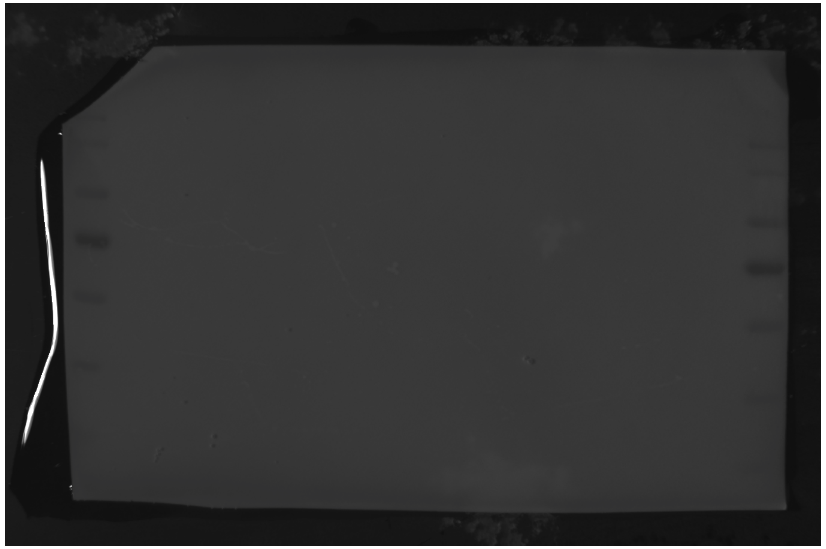

Supplement: Supplementary file 4 — SLC1A5 var WB gel [file 41419_2022_5399_MOESM4_ESM.pdf]
